# Cyclophilin *ana*Cyp40 regulates photosystem assembly and phycobilisome association in a cyanobacterium

**DOI:** 10.1038/s41467-022-29211-w

**Published:** 2022-03-30

**Authors:** Shivam Yadav, Martin Centola, Mathilda Glaesmann, Denys Pogoryelov, Roman Ladig, Mike Heilemann, L. C. Rai, Özkan Yildiz, Enrico Schleiff

**Affiliations:** 1 Institute for Molecular Biosciences, Goethe University Frankfurt, Max von Laue Str. 9, 60438 Frankfurt, Germany; 2 Centre of Advanced Study in Botany, Institute of Science, Banaras Hindu University, Varanasi, Uttar Pradesh 221005 India; 3 Max-Planck-Institute of Biophysics, Max-von-Laue-Straße 3, 60438 Frankfurt, Germany; 4 Institute of Physical and Theoretical Chemistry, Goethe University, Max-von-Laue-Strasse 7, 60438 Frankfurt am Main, Germany; 5 Institute for Biochemsitry, Goethe University Frankfurt, Max von Laue Str. 9, 60438 Frankfurt, Germany; 6 Frankfurt Institute for Advanced Studies, D-60438 Frankfurt, Germany; 7 Present Address: Department of Botany, T.P.S. College, Patna, Bihar 800001 India; 8 Present Address: Institute of Pharmaceutical Chemistry, Goethe University Frankfurt, Max von Laue Str. 9, 60438 Frankfurt, Germany; 9 Present Address: ZoBio BV, Leiden Bioscience Park, J.H. Oortweg 19, 2333CH Leiden, The Netherlands

**Keywords:** Cellular microbiology, Bacterial structural biology, Photosynthesis, Chaperones

## Abstract

Cyclophilins, or immunophilins, are proteins found in many organisms including bacteria, plants and humans. Most of them display peptidyl-prolyl cis-trans isomerase activity, and play roles as chaperones or in signal transduction. Here, we show that cyclophilin *ana*Cyp40 from the cyanobacterium *Anabaena* sp. PCC 7120 is enzymatically active, and seems to be involved in general stress responses and in assembly of photosynthetic complexes. The protein is associated with the thylakoid membrane and interacts with phycobilisome and photosystem components. Knockdown of ana*cyp40* leads to growth defects under high-salt and high-light conditions, and reduced energy transfer from phycobilisomes to photosystems. Elucidation of the *ana*Cyp40 crystal structure at 1.2-Å resolution reveals an N-terminal helical domain with similarity to PsbQ components of plant photosystem II, and a C-terminal cyclophilin domain with a substrate-binding site. The *ana*Cyp40 structure is distinct from that of other multi-domain cyclophilins (such as *Arabidopsis thaliana* Cyp38), and presents features that are absent in single-domain cyclophilins.

## Abstract (web summary)

Cyclophilins are proteins found in many organisms, where they can play roles as chaperones, in signal transduction, or other functions. Here, Yadav et al. show that a cyanobacterial cyclophilin is involved in stress responses and in assembly of photosynthetic complexes, and displays unique structural features.

## Introduction

Cyclophilins constitute a sub-family of immunophilins, next to FK-506-binding proteins (FKBPs) and parvulin-like proteins^1,2^. They can be found in all subcellular organelles and almost all forms of life, ranging from archaea to animals and plants^1–3^. Immunophilins were originally recognized as receptors for immunosuppressive drugs like the cyclic undecapeptide cyclosporin A, FK506, the macrocyclic lactones rapamycin and juglones, which hold only clinical relevance^4^. Despite of their low sequence and structural similarity, most of them possess peptidyl-prolyl cis-trans isomerase activity that catalyzes the rotation of X-Pro peptide bonds from cis to trans^1–4^. This is a rate-limiting step in protein folding and of prime importance, as 90% of all proteins comprise trans prolyl imide bonds^5,6^. Cyclophilins have been categorized into (i) single catalytic cyclophilin domain containing proteins and (ii) multidomain cyclophilins, which contain additional functional domains like WD40, RRM, TPR, PsbQ like domains, Zinc Finger domains and others^2^.

Immunophilins have diverse functions in protein folding, trafficking, maturation and scaffolding, in RNA processing, in spliceosome and in RISC assembly, stabilization and signaling of receptor complexes and in cell cycle regulation^7–12^. In plants, they further function in assembly and maintenance of photosystems, and stress responses in general^13–16^. In contrast, cyanobacterial cyclophilins remained largely unexplored. Cyanobacteria comprise a group of photoautotrophic prokaryotes and represent the ancestors of modern plastids, thereby they were of great impact in development of eukaryotic algae and plants^17^. Furthermore, diazotrophic cyanobacteria are dominant nitrogen fixers in oceans and rice fields^18^. Proteomic studies uncovered an increase of cyclophilin protein abundance in the diazotrophic *Anabaena* sp. PCC 7120 (abbreviated as *Anabaena* sp. hereafter) under salt, herbicide and UV-B stress^19–22^. This suggests an impact of cyanobacterial cyclophilins on stress response.

Cyanobacterial cyclophilins might function in photosynthetic regulation in addition to stress management. In spinach, a 40 kDa cyclophilin-type peptidyl-prolyl cis-trans isomerase (PPIase) was found to reside in the thylakoid lumen and was annotated as thylakoid lumen PPIase of 40 kDa (TLP40). The protein contains a PPIase domain and a phosphatase binding module, and negatively regulates the thylakoid protein phosphatase^13–15^. The ortholog in *Arabidopsis thaliana*, *at*Cyp38 (cyclophilin of 38 kDa) shares 82% sequence identity to the spinach protein and participates in early biogenesis of photosynthetic protein complexes^14,23^. It is assumed that *at*Cyp38 assists in D1 and possibly in CP43 folding during assembly of the oxygen evolving complex (OEC)^14^. However, the direct targets and the mode of action remain unclear. The presence of a similar cyclophilin family protein (Alr5059) in *Anabaena* sp. is remarkable, as the regulation of OEC assembly is different in cyanobacteria, because the D1 turnover is not dependent on D1 phosphorylation state^24^.

Apart from minor differences in subunit composition, the PSII-core is conserved from cyanobacteria to plants. The PSII-complex normally functions as a dimer^25,26^, whereas functional monomeric complexes have also been isolated. Those are considered as intermediates from assembly or the damage–repair cycle^27^. PSII assembly and turnover requires molecular chaperones and other auxiliary proteins which are transiently involved in the biogenesis, maintenance and stabilization of the dimers of PSII. However, the functions of many of these proteins remain poorly understood^28,29^.

PSI-complexes of cyanobacteria and plants are remarkably similar as well^25^, whereas differences exist in the occurrence of extrinsic protein subunits: (i) subunits PsaG and PsaH are present exclusively in plants, whereas PsaX and PsaM are found in cyanobacteria only, and (ii) subunit PsaL is structurally different in plants and cyanobacteria^25^. These differences cause alterations in supramolecular organization of the PSI complex between cyanobacteria and plants. The cyanobacterial PSI complex can be isolated as a monomer, trimer (most species) or tetramer^30^. In eukaryotes (algae and higher plants), PSI exists as monomer which can be part of a supercomplex with the light-harvesting Chl complex of PSI (LHCI)^26^. PsaM and PsaL are considered responsible for trimer formation in cyanobacteria^30^, whereas the PsaG subunit found in plants PSI provides an anchoring point for Lhca subunits, leading to the formation of a PSI-LHC supercomplex^31^. Moreover, the small subunits PsaF, PsaJ, PsaK, and PsaX located at the distal side of the PSI complexes may be involved in the interaction of PSI with its external antenna systems, such as IsiA or phycobilisomes^32^.

Although the PS-core shows high conservation from cyanobacteria to plants, antenna complexes are diverse. In cyanobacteria and red algae, phycobilisomes (PBS) are peripheral water-soluble antenna complexes that harvest light energy and transfer it to the photosynthetic centres^33^. PBS constitute supercomplexes, which consist of the chromophore-bearing biliproteins phycocyanin (PC), allophycocyanin (APC) and phycoerythrin (PE). Those are connected by non chromophorylated linker (L) proteins. L-proteins also stabilize and regulate PBS quaternary structure, and by that optimize the energy transfer^33^. PBS transfer energy to PSII and to PSI in a megacomplex in cyanobacteria^34^, or to PSI as discussed in alternative models^35^. The coexistence of alternative complexes is seen as indication for high flexibility of the PBS-PS complex formation. In contrary to the cyanobacterial system, the higher plant light-harvesting complex of PSII (LHCII) (genes: *lhcb1-3*) constitutes about one-third of the total chloroplast protein content. LHCII trimers bind half of the total chlorophyll located in the thylakoid membrane, whereas the LHCI antenna (genes: *lhca1-4*) associate entirely with PSI^36^.

Taking recourse to above, we report the function of a multidomain cyclophilin (47% identical to *at*Cyp40) from *Anabaena* sp. through generation of *Anabaena* sp. single recombinant mutants, the in vivo analysis of the localization and the interactome, as well as by determination of the crystal structure of the protein.

## Results

### The gene encoded by *alr5059* is a cyclophilin family member

**Fig. 1:**
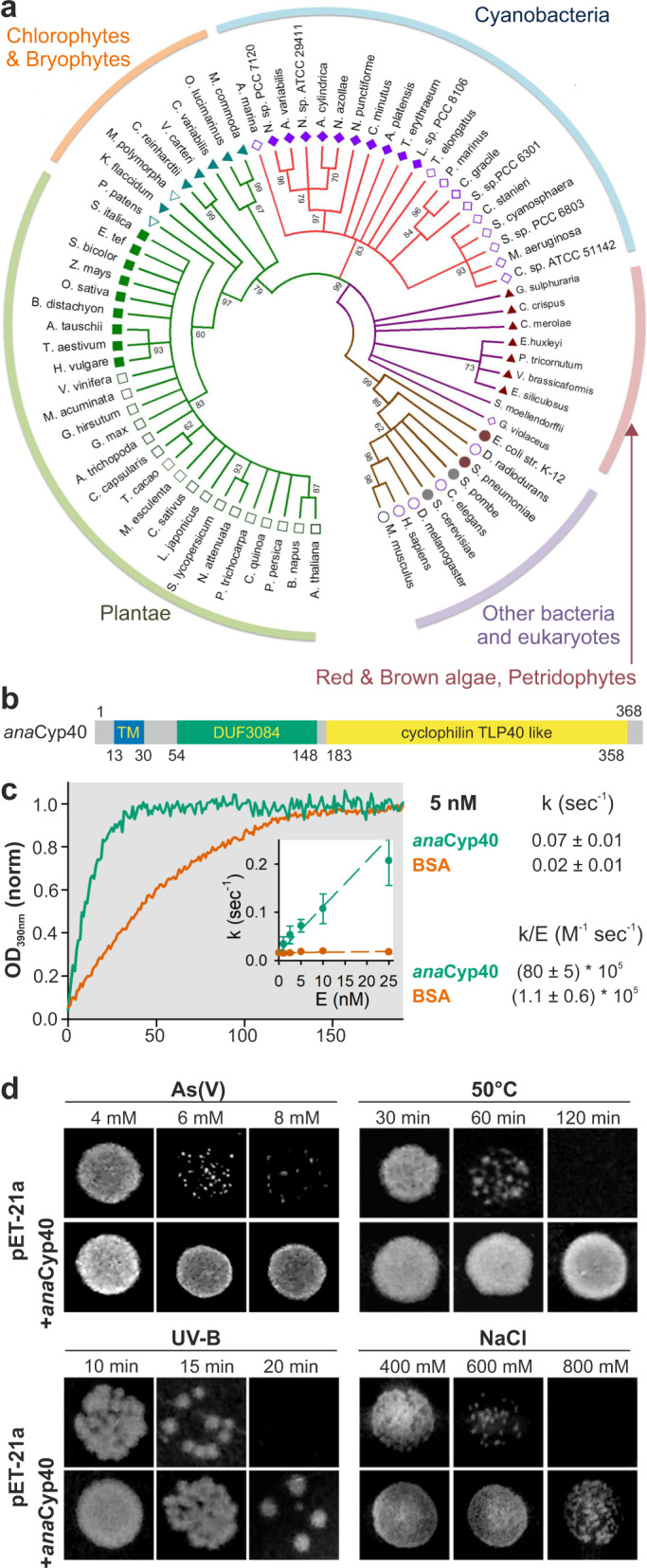
The protein encoded by alr5059 shows a sequence similarity to *at*Cyp38. **a** Shown is the phylogenetic analysis of the sequences of the plant and cyanobacterial cyclophilin family as described in Materials and Methods (Supplementary Table 1). **b** The predicted domains of *ana*Cyp40 are shown as bar diagram. The conserved domains were extracted from NCBI database (CDD)^91^ and additional regions for membrane anchoring or targeting predicted as described (Supplementary Table 2). TM: transmembrane region, DUF: domain of unknown function. **c** Purified *ana*Cyp40_ΔTM_-His (5 nM, blue) or BSA (5 nM, red) were incubated with 40 μM N-succinyl-ala-ala-pro-phe-p-nitroanilidine and the catalytic reaction monitored by the increase of absorption at 390 nm. Source data are provided as a Source data file. The values were normalized to the baseline and to the maximum. A representative experiment is shown. The average of the determine rate constant (*n* > 5 repetitions of the experiment) is presented and the standard deviation is shown as error bar. **d** *E. coli* BL21 (DE3) transformed with pET21a (upper panel) or pET21a-*ana*Cyp40 (lower panel) were spotted on agar plates or plates containing the indicated concentrations of arsenic (upper left) or sodium chloride (lower right). Bacteria on normal plates were exposed to 50 °C or to UV-B (2.9 mWm^−2^ nm^−1^) for the indicated time. Representative images of growth after 24 h are shown.

The 40 kDa protein encoded by *alr5059* from *Anabaena* sp. is most similar to bacterial cyclophilins or plant thylakoid lumen PPLases (TLP40, *at*Cyp38), therefore it is annotated as *ana*Cyp40. The computed phylogenetic relation shows a global conservation of *ana*Cyp40 in cyanobacteria, algae chlorophytes, bryophytes and plantae (Fig. 1a, Supplementary Table 1). Sequence analysis of *ana*Cyp40 shows the presence of a C-terminal cyclophilin domain and an N-terminal conserved domain of unknown function (Fig. 1b). The utilized transmembrane domain prediction programs (Supplementary Table 2) consistently predict a transmembrane domain (TM) between amino acids 13 and 30. A putative signal sequence (Sec/SPI) of about 36 amino acids length was predicted at the N-terminal region including the TM domain, though with a low likelihood of 0.51 (SignalP)^37^. Worth mentioning, the predicted cleavage site (Signal P) stands in contrast to the N-terminal peptide obtained by mass spectrometry, predicting a polypeptide starting at amino acid 15^38^. The cyanobacterial and plant proteins differ in their signal sequence, the region preceding the transmembrane domain and in the region annotated as domain of unknown function (DUF3084)^39^.

To test the catalytic activity of *ana*Cyp40, a recombinant version without the N-terminal transmembrane helix (aa 1–35) but with C-terminal His tag was expressed in *E. coli*. The purified protein was used to hydrolyze N-succinyl-ala-ala-pro-phe-p-nitroanilidine in order to determine a putative peptidyl prolyl cis-trans isomerase activity^40^. For *ana*Cyp40 an activity with a rate of 0.07 ± 0.01 sec^−1^ was observed, while a background rate of 0.02 ± 0.01 s^−1^ was determined for bovine serum albumin (Fig. 1c; Supplementary Fig. 1; BSA). The measured rate constant of purified *ana*Cyp40 is comparable to the activity reported for homologs from other organism (Supplementary Table 3).

Next, the protective capacity of *ana*Cyp40 against specific stresses was probed, to further confirm the assignment as cyclophilin. The recombinant protein was expressed in *E. coli*, which was subsequently exposed to arsenic, salt, UV-B or heat stress. At high doses of the stressors, *E. coli* transformed with the empty plasmid showed a clear growth defect, while the bacteria transformed with the protein coding plasmid were resistant to elevated stress levels (Fig. 1d). This result is consistent with the functional assignment of *ana*Cyp40 as cyclophilin.

### A functional *ana*Cyp40 is required for cyanobacterial salinity stress response

**Fig. 2:**
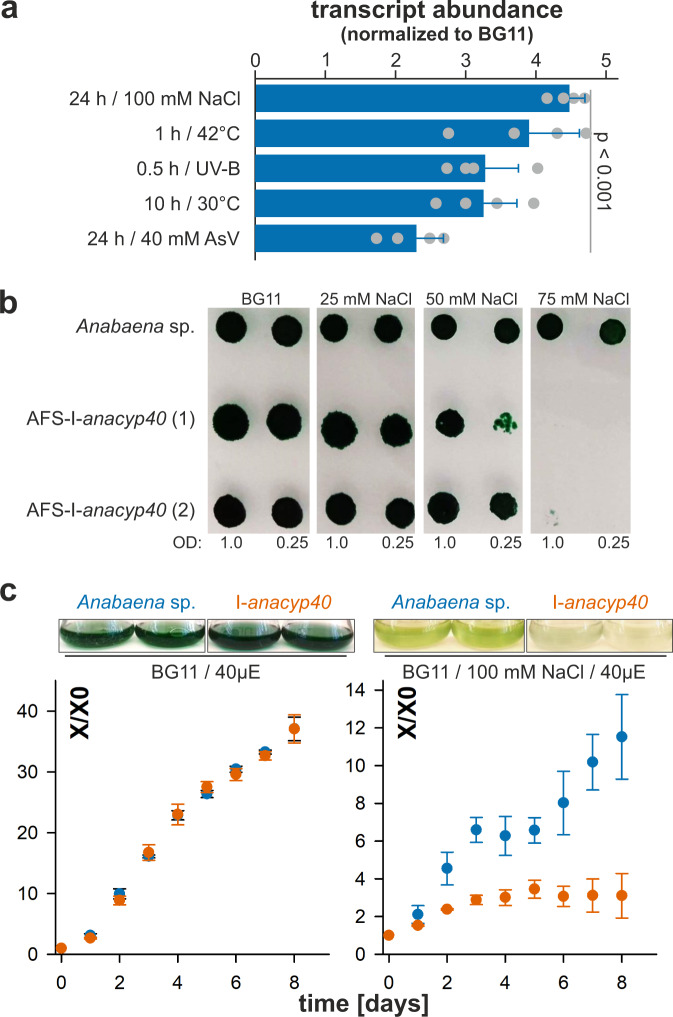
*ana*Cyp40 is required for salinity stress response. **a** Wild-type *Anabaena* sp. was grown in BG11 and cells were treated as indicated followed by RNA isolation and qRT-PCR analysis with specific oligonucleotides for *anaCyp40* (Supplementary Table 4). The mean of the ratio to the mRNA abundance in non-treated cultures is shown and error bars indicate the standard deviation (*n* = 4). The statistical significance determined by ANOVA (Duncans) of the difference to non-treated cultures is indicated. **b** Two independently generated AFS-I-a*naCyp40* strains and wild-type cells were spotted at two indicated concentrations at BG11 supplemented with indicated concentrations of NaCl. The plates were imaged after 8 days of growth. **c** Wild-type (blue) and AFS-I-a*naCyp40* (orange) were grown in BG11 liquid medium (left) with additional 100 mM NaCl (right). The culture density was imaged after 8 days (top) and the average values and the standard deviation for the growth of three independent cultures normalized to the OD at the start of the analysis are shown (bottom). Source data are provided as a Source data file.

The *ana*Cyp40-coding gene is expressed under normal growth conditions *of Anabaena* sp., while the mRNA abundance is enhanced in response to abiotic stress. qRT-PCR analysis on isolated mRNA showed that the exposure of *Anabaena* sp. to arsenic, to desiccation by incubation at 30 °C for 10 h, to heat stress at 45 °C, to enhanced salinity by addition of 100 mM sodium chloride or to UV-B stress by application of 2.9 mW m^−2^ nm^−1^ for 30 min enhances the transcript abundance of *anacyp40* by 2.5–4.5 fold compared to normal growth conditions (Fig. 2a). Hence, *anacyp40* transcription is regulated in response to various abiotic stresses, and appears to be required for cellular homeostasis of *Anabaena* sp. This is consistent with the observed ability of *ana*Cyp40 to enhance the stress resistance of *E. coli* (Fig. 1).

An insertion mutant created by single recombination and annotated as AFS-I-*anacyp40* (*Anabaena* sp. mutant created in Frankfurt by Schleiff group with plasmid insertion)^41^ was generated to explore the function of *ana*Cyp40 in cyanobacterial stress response (Supplementary Table 4). The insertional mutant was segregated, documenting that *ana*Cyp40 is not essential under normal growth conditions (Supplementary Fig. 2). It was reported recently that an antisense RNA to *alr5059* is expressed in the absence of fixed nitrogen, which might be involved in heterocyst differentiation^42^. However, the strategy of mutant generation does not inhibit the induction of the antisense RNA in the absence of fixed nitrogen (Supplementary Fig. 2). Thus, the phenotype of the mutant is dedicated to the loss of *ana*Cyp40 function.

In total, three independent strains of AFS-I-*anacyp40* were isolated and analyzed. All three strains had the same phenotype as exemplified for the strain (1) and (2) (Fig. 2b). Subsequently, the results for strain (1) are shown as representative if not otherwise noted. Consistent with the expression profile of *anacyp40*, AFS-I-*anacyp40* showed an enhanced sensitivity to salt stress (Fig. 2b). The growth of the mutant strain on solid BG11 medium under standard light conditions was comparable to wild-type *Anabaena* sp., but was inhibited on solid BG11 medium containing 75 mM NaCl. Similarly, the mutant strain does not show any growth distinction to wild type in liquid BG11 medium (Fig. 2c, left), but is reduced in the presence of 100 mM NaCl (Fig. 2c, right). Worth mentioning, the growth of wild-type *Anabaena* sp. is reduced in the presence of 100 mM NaCl as well when compared to the growth of this bacterium in normal media.

### *Ana*Cyp40 interacts with different proteins and complexes

The importance of *ana*Cyp40 in stress response (Figs. 1, 2) prompted the analysis of the substrate spectrum of the cyanobacterial cyclophilin. For isolation of cyclophilin-substrate complexes a strain coding for the protein bearing a C-terminal Strep-tag was created (AFS-*anacyp40*-strep; Supplementary Fig. 4). The AFS-I-*anacyp40-strep* strain exhibited a phenotype comparable to the wild type in response to enhanced salinity (Supplementary Fig. 5), indicating that the function of *ana*Cyp40 is not impaired by the Strep-tag. The complex formation of *ana*Cyp40 was probed by mass spectrometry of proteins after affinity tag purification of the Strep-tagged protein from cell lysate (Supplementary Fig. 4). In total, 65 proteins have been detected with a p-value smaller than 0.05 (Table 1, Supplementary Data 2).

**Table 1:** Proteins detected to interact with *ana*Cyp40.

Category	Gene ID	Name	*p*-value	ExpD.	MP	aa	% P
BAIT	Alr5059	BAIT	1 × 10^−3^	3	40		
Ribosome	All4199	S5	1 × 10^−3^	3	10	174	3.4
	All4202	S8	1 × 10^−3^	3	11	133	3.0
	All4203	L5	1 × 10^−3^	3	12	182	4.4
	All4210	L22	1 × 10^−3^	3	5	119	5.0
	All4213	L23	1 × 10^−3^	3	10	104	9.6
	All4205	L14	2 × 10^−3^	2	10	122	4.1
	All4214	L4	2 × 10^−3^	2	11	210	3.3
	All4792	S2	2 × 10^−3^	2	14	265	3.8
	Alr5297	L19	2 × 10^−3^	2	8	120	2.5
	Alr5301	L1	2 × 10^−3^	2	19	238	4.6
	Asr0742	S21	2 × 10^−3^	2	10	62	1.6
	All4198	L15	0.016	2	11	148	4.7
	All4200	L18	0.016	2	11	120	2.5
	All4336	S10	0.016	2	14	105	5.7
	All4339	S7	0.016	2	11	156	3.8
RNA poly-merase	All4191	RpoA	1 × 10^−3^	3	17	315	4.4
	Alr1594	RpoB	1 × 10^−3^	3	28	1131	5.7
	Alr1596	RpoC2	1 × 10^−3^	2	51	1355	4.1
	Alr1595	RpoC1	0.016	2	13	625	5.4
	Asr4648	RpoO	0.016	2	3	78	2.6
Putative substrate	All1075		1 × 10^−3^	3	4	294	4.4
	All3822	Ycf27	1 × 10^−3^	2	10	242	4.1
	All4008		1 × 10^−3^	3	11	589	4.1
	All4338	FusA	1 × 10^−3^	3	11	692	4.8
	All4358	ClpP	1 × 10^−3^	3	7	220	9.1
	All4779	Ssb3	1 × 10^−3^	3	5	182	14.3
	Alr0088	Ssb1	1 × 10^−3^	3	6	119	3.4
	Alr0128	ChlP	1 × 10^−3^	3	12	406	4.4
	Alr1890		1 × 10^−3^	3	13	526	4.4
	Alr2268	PurC	1 × 10^−3^	3	4	245	3.3
	Alr2350		1 × 10^−3^	3	10	313	7.0
	Alr2372		1 × 10^−3^	3	4	339	4.4
	All3272	RecA	1 × 10^−3^	3	4	357	2.5
	Alr3276		1 × 10^−3^	3	13	312	7.1
	Alr3768	OrrA	1 × 10^−3^	3	4	240	2.9
	Alr3952	DevH	1 × 10^−3^	3	7	239	5.4
	All2315	IlvC	2 × 10^−3^	2	10	331	3.6
	All2566	Gap1	2 × 10^−3^	2	8	343	4.7
	All3680		2 × 10^−3^	2	5	317	6.6
	Alr0946		2 × 10^−3^	2	19	144	5.6
	Alr1526	CbbS	2 × 10^−3^	2	12	109	7.3
	Asr0105		2 × 10^−3^	2	5	88	3.4
	All5265	GyrB	4 × 10^−3^	2	5	645	3.4
	All7614		4 × 10^−3^	2	7	547	4.0
	Alr4798	ArgG	4 × 10^−3^	2	3	400	5.7
	All0865	CcmM	0.016	2	26	555	4.3
	All2777	RbpE	0.016	2	3	99	2.0
	All4623	InfC	0.016	2	9	177	5.1
	Asr3935	Hup	0.016	2	7	94	5.3
PBS	Alr0530	CpcC	1 × 10^−3^	3	38	266	3.5
	Alr0535	CpcG2	1 × 10^−3^	3	10	247	6.1
	Alr0020	ApcE	8 × 10^−3^	3	92	1132	6.0
	Alr0021	ApcA1	0.018	3	21	161	3.1
	Asr0531	CpcD	0.023	3	6	80	1.2
	Alr0537	CpcG4	0.042	3	15	253	3.2
	Asr0023	ApcC	0.046	3	16	68	2.9
	Alr0525	PecC	0.047	3	19	278	2.5
	Alr0022	ApcB	0.049	3	14	162	1.9
PSII	All3854	PsbO	1 × 10^−3^	3	9	273	4.8
	All3076	PsbP	0.016	2	2	246	4.9
	Alr4291	PsbC	0.031	2	4	459	4.8
PSI	Asr4319	PsaE	0.031	2	4	70	4.3
ATP synthase	All0004	AtpC	1 × 10^−3^	3	7	315	3.5
	All5039	AtpD	1 × 10^−3^	3	23	482	5.2

Given are the putative category, gene ID and name, *p*-value (as described in “Methods”), number of experiments the protein was detected in, maximal number of peptides identified in one of the seven replicas (MP), number of amino acids and percentage of prolins (%P) in the sequence. The average proline content in all proteins of the *Anabaena* sp. proteome is (4.6 ± 2.0%).

Many, but not all proteins of the ribosomes and the RNA polymerase were precipitated with *ana*Cyp40 as bait. This could suggest that *ana*Cyp40 interacts with substrates during translation. Notably, the cell lysate was not treated with RNAse or DNAse, which would disrupt RNA-polymerase - ribosome complexes^43^. In favor of this interpretation is the discovery of proteins from small and large ribosomal subunits, the former interacting with the RNA polymerase and the latter representing the proteins at the ribosomal exit tunnel. Interestingly, the PPIase-like domain containing trigger factor anchors to the ribosome through interaction between its tail domain and L23, being in vicinity of L29 and L19^44^. In here, L23 and L19 were identified among the ribosomal proteins. Alternatively, ribosomal and polymerase proteins could also be substrates of *ana*Cyp40, because all of them contain at least one proline (Table 1). However, in the latter case, a broader range of precipitated ribosomal proteins would have been expected^45^.

A second group represented by 29 proteins presumably involves substrates of *ana*Cyp40, as these could not be assigned to a specific functional complex (Table 1). All of these proteins contain a high number of prolines, and especially proteins like ClP, Ssb3 or CbbS have a higher proline content than the average in the theoretical proteome of *Anabaena* sp. Thus, an assignment as substrates would be consistent with the proposed function of *ana*Cyp40.

A third category unifies proteins involved in photosynthesis, with the highest abundance found for PBS components (Table 1). Some of these genes belong to the highest expressed genes in *Anabaena* sp. wild-type cells^46^, which could argue for a classification as substrate. However, this does not hold true for all genes coding for the identified PBS proteins and in turn, not all PBS proteins encoded by highly expressed genes, or containing a large number of prolines, were identified (Supplementary Table 5). This might suggest that *ana*Cyp40 has an additional function related to photosynthetic performance.

### *Ana*Cyp40 is important for the photosynthetic capacity of *Anabaena* sp. PCC 7120

**Fig. 3:**
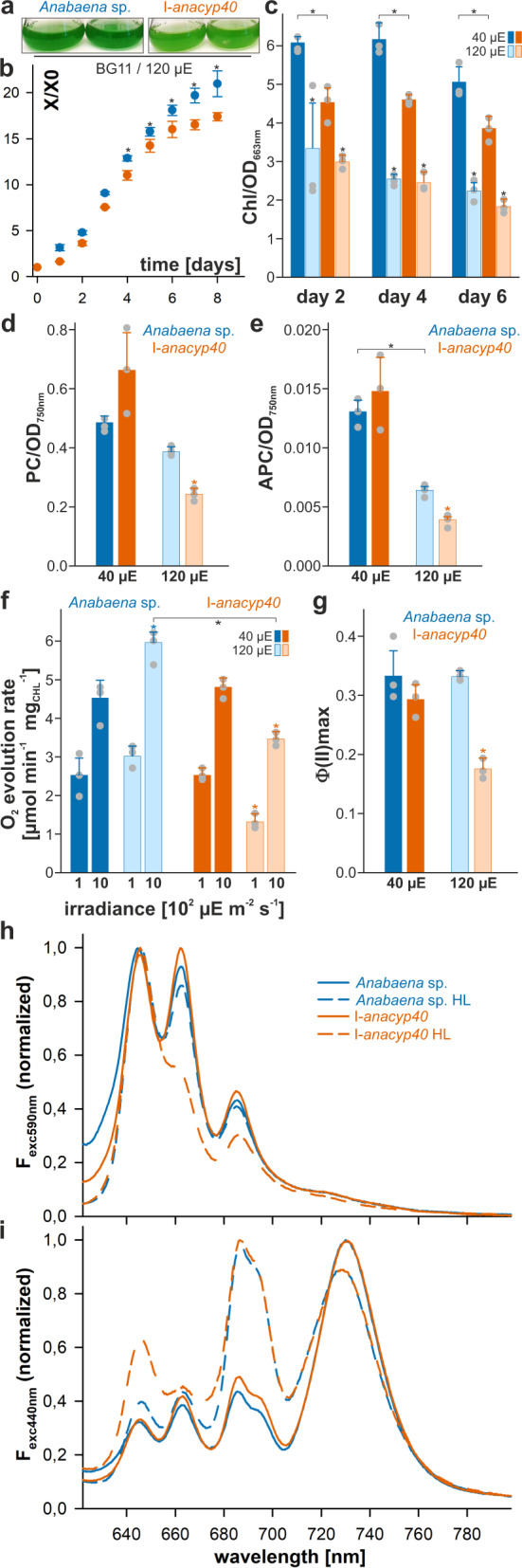
*ana*Cyp40 is important for the photosynthetic performance under high-light. **a**, **b** Wild-type (blue) and AFS-I-*anaCyp40* (orange) were grown in BG11 liquid medium at 120 µE illumination (right). The culture density was imaged after 8 days (**a**) and the average values for the growth of three independent cultures normalized to the OD at the start of the analysis are shown (**b**). **c** The chlorophyll content in wild-type (blue) and AFS-I-a*naCyp40* (orange) cells grown under 40 µE or 120 µE (transparent color) was analyzed and normalized to cell density for three independent experiments. **d**, **e** The PC (**d**) and the APC (**e**) content in wild-type (blue) and AFS-I-a*naCyp40* (orange) cells grown under 40 µE or 120 µE (transparent color) was analyzed and normalized to cell density. The statistical significance was analyzed and *p* < 0.001 is indicated. **f** The oxygen evolution rate was determined for wild-type (green) and AFS-I-a*naCyp40* (orange) grown in BG11 liquid medium at 40 (dark color) or 120 µE illumination (transparent color). The average values and the standard deviation are shown. Stars indicate statistical significance with *p* < 0.001. **g** The photosynthetic parameters were determined by PAM. The Φ(II) for wild-type and mutant culture grown at 40 or 120 µE are shown. The statistical significance of the change was analyzed for same illumination condition and *p* < 0.001 is indicated. Error bars in **b**–**g** indicate standard deviation and the bare represents the mean. In **b**–**g** the statistical significance was analyzed by ANOVA (Duncans) and the star indicates a *p* < 0.001. **h**, **i** The 77 K fluorescence emission spectra of *Anabaena* sp. wild-type (green) or the insertion mutant of *anaCyp40* (I-*anaCyp40*, orange) grown at normal (solid line) or high-light (dashed line) were recorded by excitation with 590 nm (**f**) or 440 nm (**g**). Normalization was done at 800 nm, and the spectra are the average of at least three independent biological replicates. The color and line coding shown in (**f**). Source data are provided as a Source data file.

Consistent with a function of *ana*Cyp40 in regulation of photosynthesis, the mutant strain was significantly reduced in its growth when cultivated at 120 µE illumination (Fig. 3a, b). Interestingly, the chlorophyll content, but not the phycocyanin or allophycocyanin content, was significantly reduced in the mutant compared to wild type under normal growth conditions (Fig. 3c–e). The chlorophyll, phycocyanin or allophycocyanin content was generally reduced in wild type and AFS-I-*anacyp40* under high-light conditions. Here, the difference of the chlorophyll content between the wild-type and mutant strain was not significant, while the phycocyanin or allophycocyanin content was significantly lower in the mutant.

The analysis of the oxygen evolution revealed a comparable efficiency of wild type and AFS-I-*anacyp40* when grown under normal light conditions in BG11 (Fig. 3f). Cultivation under high-light conditions resulted in an enhanced maximal rate of net oxygen production at light saturation (Pm) at 1000 µE m^−1^ s^−1^ in wild type, but a reduced oxygen production in the mutant (Fig. 3f, Supplementary Table 6). Moreover, the compensation irradiance at which oxygen production and oxygen consumption are balanced (Ec), that can be estimated from Pm and the measurement at 100 µE m^−1^ s^−1^, was enhanced in AFS-I-*anacyp40* when grown under high light (Fig. 3f, Supplementary Table 6). The maximal effective PSII quantum yield (Φ(II)) probed by pulse-amplitude modulation (PAM-)measurements was comparable between wild-type *Anabaena* sp. and AFS-I-*anacyp40* grown under normal light (Fig. 3g, left). In contrast, a significant reduction of the maximal effective PSII quantum yield was observed for the mutant when compared to wild-type after growth at high-light (Fig. 3g, right).

Subsequently the 77 K fluorescence spectra was recorded. Excitation of the PBS at 590 nm (Fig. 3h) resulted in a large phycocyanin (~650 nm) and allophycocyanin peak (~665 nm), as well as in a somewhat smaller peak at ~685 nm likely representing the fluorescence of ApcE (~680 nm) and PSII (~685–695 nm)^47–50^. The shoulder at ~720–740 nm is related to the fluorescence of PSI. Under normal light conditions, a difference between mutant and wild-type strain was not observed (Fig. 3h, solid lines). When grown under high light, especially the allophycocyanin and the PSII fluorescence was reduced in the mutant, while this fluorescence was only moderately affected in wild type (Fig. 3h, dashed lines). Excitation of the chlorophyll at 440 nm yielded highest fluorescence of PSI (~720–740 nm) under normal light conditions, and a comparable fluorescence of PSI and PSII (~685–695 nm) at high light (Fig. 3i, dashed lines). In addition, a basal fluorescence of the PBS is observed as well (~650 nm/~665 nm). Remarkably, the mutant strain grown under high light showed an enhanced phycocyanin peak when compared to the wild type.

The apparent energy-transfer from PBS to PSI in relation to the energy transfer from PBS to PSII deduced from the ration F_PSI_/F_PSII_ after PBS excitation^51^ does not change in the mutant or through high-light treatment (Supplementary Table 6). The ratio of the activity of the two photosystems, which was calculated based on chlorophyll excitation changed in response to high-light, but again, not through mutagenesis of gene coding for *ana*Cyp40.

### The dimerization of photosystem I is affected in the *ana*Cyp40 mutant

**Fig. 4:**
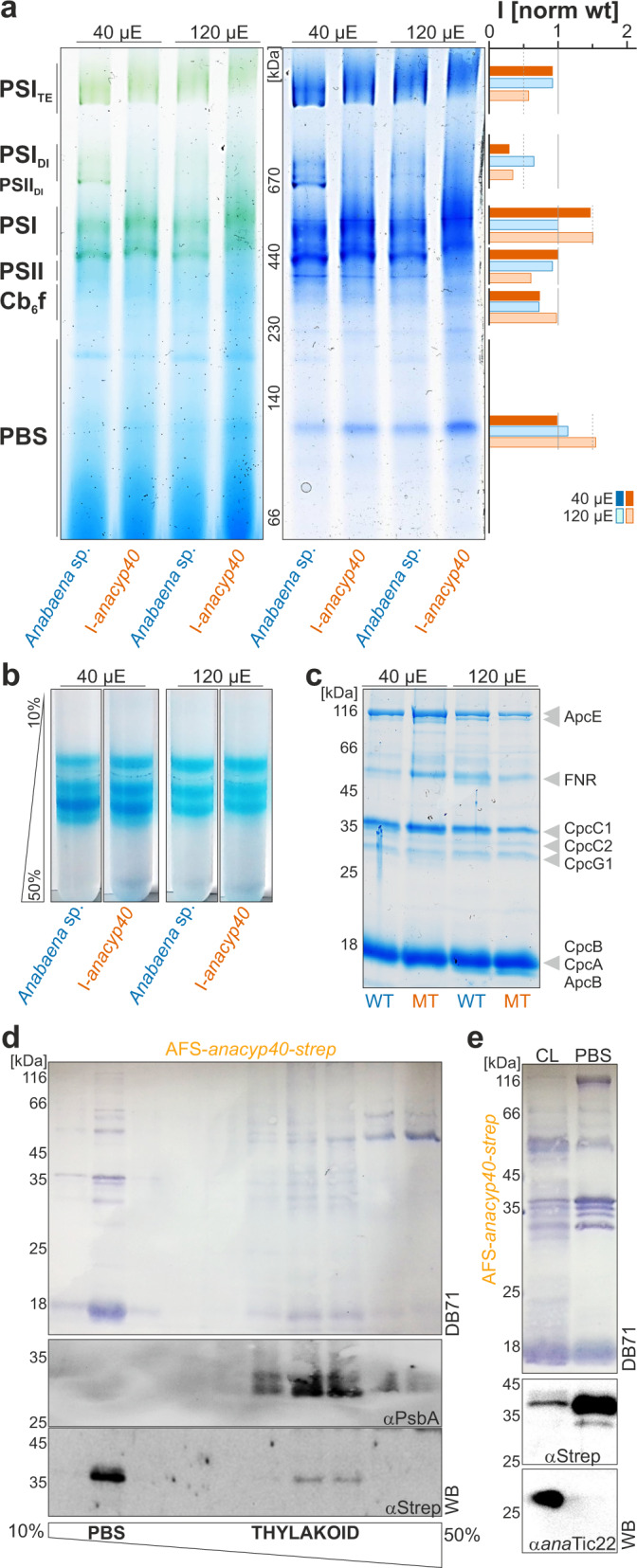
The impact of *ana*Cyp40 on assembly of photosynthetic complexes. **a** Membranes of wild-type (green) and AFS-I-a*naCyp40* (orange) grown in BG11 liquid medium at 40 or 120 µE illumination were solubilized and subjected to BN-PAGE. The chlorophyll staining (left) and the Coomassie Blue staining (right) are shown. On the left migration of complexes and between the two images the migration of the molecular weight standards is show. The Coomassie Blue staining was quantified by ImageJ and the intensity of the individual complexes was normalized to the intensity in wild-type grown at 40 µE light intensity. **b** Phycobilisomes were isolated from cultures grown as in (**a**) and subjected to a 10–50% sucrose gradient. A representative profile is shown. **c** Equal amounts of phycobilisomes (5 µg protein) isolated from the strains as in (**a**) were subjected to SD-PAGE as indicated. The migration of the molecular weight is shown on the right and the proteins are assigned^54^. **d** The strain AFS-*anaCyp40*-strep was grown in BG11, cells harvested and solubilized in low concentrated buffer and subjected on top of a 10–50% sucrose gradient. The fractions were subjected to SDS-PAGE followed by Western blotting (DB71-staining shown) and incubation with indicated antibodies. PBS and Thylakoid membrane fractions are indicated. **e** Cell lysate (CL) and phycobilisomes (PBS) were isolated in buffer with high concentration of phosphate from AFS-*anaCyp40*-strep and subjected to SDS-PAGE followed by Western blotting (DB71 staining shown) and incubation with indicated antibodies.

The influence of the mutation on the assembly of complexes involved in photosynthesis was determined by BN-PAGE analysis, using isolated membranes of *Anabaena* sp. wild type and AFS-I-*anacyp40* grown under normal or high-light conditions. Thylakoid membranes were solubilized by addition of 1% n-Dodecyl β-D-maltoside. At normal growth conditions, the abundance of the PBS, the cytochrome b_6_f complex and PSII was not affected in the mutant, while the PSII dimer was not detected in AFS-I-*anacyp40* (Fig. 4a; Supplementary Fig. 3). In contrast, the abundance of the monomeric PSI is enhanced, while the level of the dimeric state of PSI is reduced. Remarkably, the abundance of the tetrameric state is only moderately affected (Fig. 4a, PSI_TE_). When grown at high light, the wild type shows the typical reduction of the dimeric PSI. Moreover, the PSII dimer is reduced under strong light as well. In the mutant, PBS and monomeric PSI were enriched, while the dimeric and tetrameric state of PSI was reduced when compared to wild type. Hence, *ana*Cyp40 influences PSI dimerization, and one explanation could be that the functions of PsaL might have been lost, which is discussed to be important for dimerization of PSI^52,53^.

### *Ana*Cyp40 localizes to thylakoids and physically interacts with the phycobilisomes

The changes of PBS association with membranes in the mutant strain (Fig. 4a) led to the analysis of the cellular PBS content and composition. The PBS were isolated and fractionated on a 10–50% sucrose gradient (Fig. 4b). While an alteration between normal and high-light conditions was observed, the sub-complex profile was not affected in the mutant strain. When isolated phycobilisomes were subjected to SDS-PAGE, comparable profiles annotated according to previous reports^54^ were observed as well (Fig. 4c). Only an increase of ApcE was detected in AFS-I-*anacyp40*.

To further probe for an interaction of *ana*Cyp40 with phycobilisomes, AFS-*anacyp40*-strep (Supplementary Figs. 4, 5) cells were lysed and the cell lysate was fractionated by sucrose gradient centrifugation. A small fraction of *ana*Cyp40 migrated with the thylakoid membrane fraction, identified by the protein profile and the immunodetection of PsbA (Fig. 4d; Supplementary Fig. 6). Most of the protein migrated at a lower density fraction that contained PBS as judged from the protein profile (Fig. 4d) and the color of the fraction (not shown). To distinguish between an interaction with PBS and a co-migration of *ana*Cyp40 in this fraction, PBS total lysate was isolated from AFS-*anacyp40*-strep, and probed with antibodies against the strep tagged protein or the periplasmic localized *ana*Tic22^55^. While both proteins were detected in the cell lysate (Fig. 4e, CL; Supplementary Fig. 7), the Strep-tagged *ana*Cyp40 protein was enriched in the PBS fraction (Fig. 4e, PBS).

**Fig. 5:**
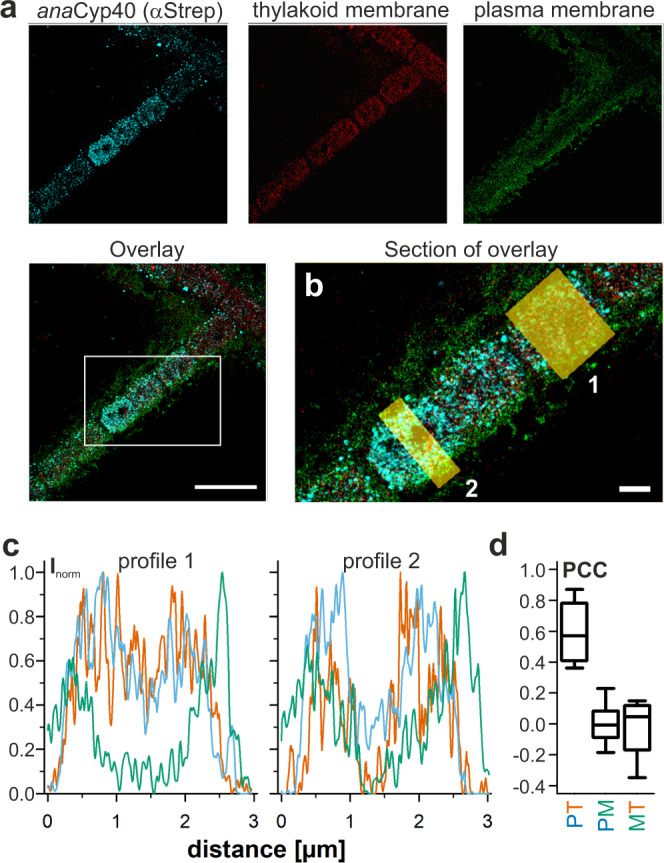
The intracellular localization of *ana*Cyp40. **a** AFS-*anaCyp40*-strep was grown in BG11 and processed as described in “Methods”. Using three-color super-resolution microscopy, the nanoscale localization of *ana*Cyp40 (left), the thylakoid membrane (second from left) and the plasma membrane (second from right) were visualized (overlay shown on the right, scale bar = 5 µm). **b** Enlarged section as indicated in (**a**) with two boxed regions (1, 2) from where intensity profiles were extracted (scale bar = 1 µm). **c** Intensity profiles for the two regions shown in (**b**) for *ana*Cyp40 (cyan), the thylakoid membrane (red) and the plasma membrane (green) are shown. **d** The Pearson correlation coefficient (PCC) was calculated from intensity values for pixels and for *ana*Cyp40 and thylakoid (PT), *ana*Cyp40 and membrane (PM) as well as for the membrane and thylakoid (MT). Values were calculated in 10 individual regions and are shown as box plot (horizontal lines indicate median, box sizes represent the 25th and 75th percentile of all values, whiskers indicate the standard deviation). Source data are provided as a Source data file.

The association with the phycobilisomes prompted the analysis of the intracellular localization of *ana*Cyp40 with near-molecular spatial resolution. We used single-molecule super-resolution microscopy in combination with DNA-labeled antibodies (DNA-points accumulation for imaging in nanoscale topography, DNA-PAINT)^56^ to determine the nanoscale structural organization of *ana*Cyp40 in cells. The localization of the Strep-tagged protein was visualized in AFS-*anacyp40*-strep together with endogenous fluorophores in the thylakoid membrane. The outer membrane and plasma membrane were stained with NileRed (Fig. 5a). The endogenous fluorophores of *Anabaena* sp. exhibited a photoswitching behavior and were sufficiently bright and photostable to perform single-molecule detection, which allowed tracing the thylakoid structure in cells. After photobleaching of the auto-fluorescence signal, a DNA imager strand was added to the imaging buffer in order to visualize AFS-*anacyp40*-strep in the same cells. The super-resolution images show the distribution of the anti-Strep signal inside the cell (Fig. 5a, overlay). The spatial resolution of the super-resolution images was determined by decorrelation analysis to be 24 nm (Supplementary Fig. 8)^57,58^. A co-localization analysis from intensity profiles (Fig. 5b) showed a spatial separation of the NileRed and the anti-Strep signal, but a co-occurrence of the thylakoid localized auto-fluorescence and the anti-Strep signal. The calculation of the Pearson correlation coefficient (PCC) of multiple regions indicates a co-localization of *ana*Cyp40 with thylakoid membranes, but not with the membrane system surrounding the cell (Fig. 5c). This is consistent with the obtained phenotype, as well as with an association of *ana*Cyp40 with phycobilisomes. The specificity of the antibody staining was confirmed in wild type *Anabaena* sp. (Supplementary Fig. 9).

### *Ana*Cyp40 contains two functional domains

**Fig. 6:**
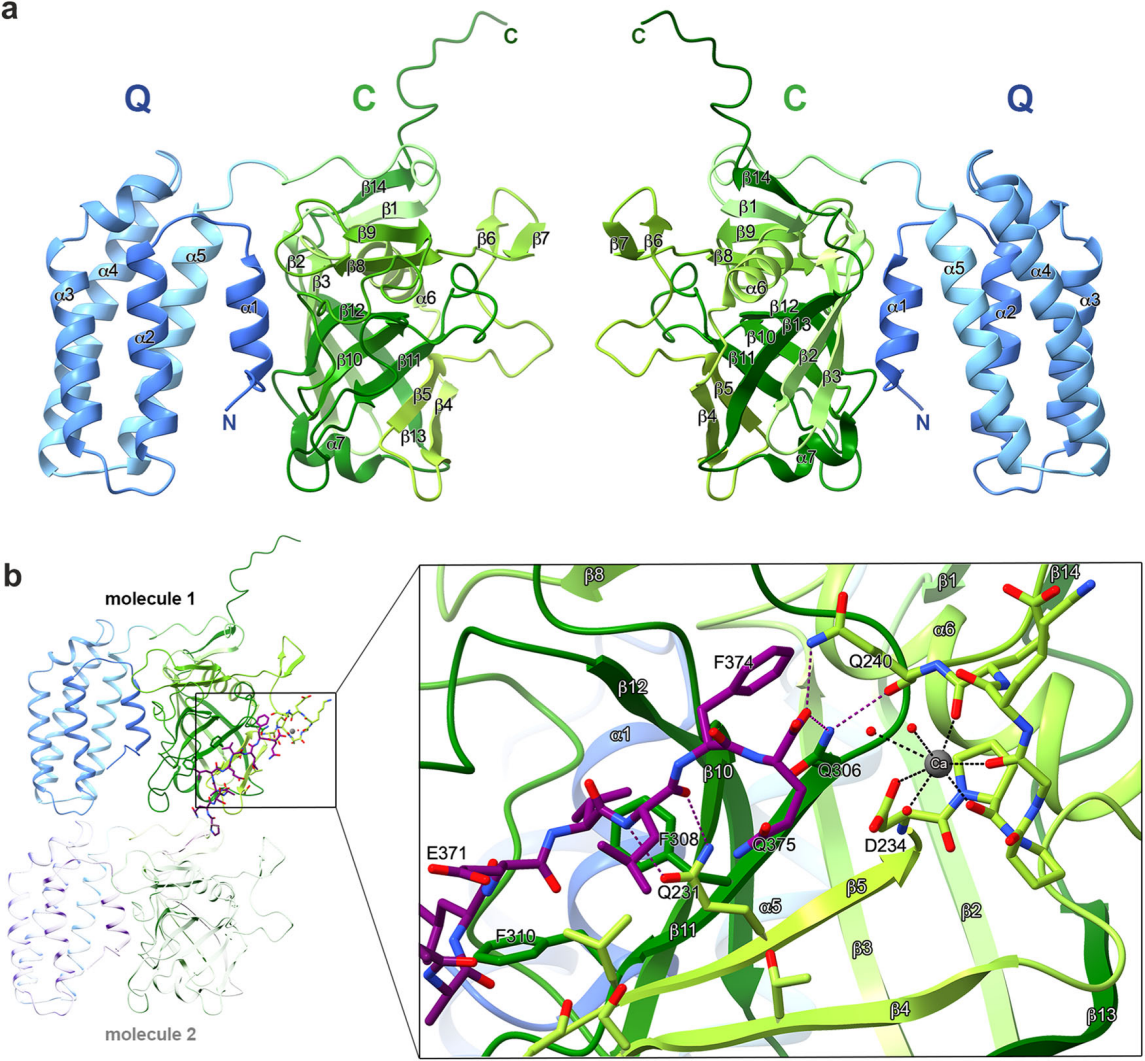
Overall structure of *ana*Cyp40. **a** Ribbon presentation of *ana*Cyp40 from two orientations related to each other by a 180° vertical rotation. The N-terminal four-helix bundle (Q-domain) is shown in blue and the cyclophilin domain (C-domain) is shown in green colors. The two domains are linked by a flexible loop. The interaction between the domains is mediated by the N-terminal helix (α1). The cyclophilin domain is formed by a distorted β-barrel with eight β-strands (β2–5 and β10–13). The β-barrel is flanked on one side by helix α6 and the remaining β-strands and on the other side by helix α7. The N- and C-termini are indicated. **b** The intermolecular interactions between two symmetry-related *ana*Cyp40 molecules are shown on the left side. The interaction is mediated by the last seven C-terminal residues shown in purple. The boxed region is magnified on the right side and shows the interactions of the C-terminal peptide with the potential substrate binding site on the outer surface of the β-barrel. The metal at the site within close proximity to the C-terminus of the symmetry-related monomer was interpreted as heptagonal coordinated calcium ion (Ca^2+^) that obviously is part of the active site of *ana*Cyp40.

A more precise characterization of the enzymatic function of *ana*Cyp40 at a molecular level requires high-resolution structural information. To achieve this, high-quality single crystals of *ana*Cyp40 lacking the N-terminal trans-membrane helix (*ana*Cyp40_ΔTM_) were grown by vapor diffusion in hanging drops^39^ and used to collect diffraction data to 1.2 Å (Supplementary Table 7). The structure was determined by molecular replacement with *at*Cyp38 (pdb-id: 3rfy)^23^ as search model. The asymmetric unit contains one *ana*Cyp40 molecule with two domains: a helical N-terminal domain and a C-terminal cyclophilin domain (Fig. 6a). Except of five N-terminal amino acids, all residues could be traced and are present in the final model. This includes the “LEVLFQ” sequence from the PreScission protease recognition site at the C-terminus that remained after the cleavage of the purification tag. The C-terminal region contributes to the crystal contacts between the individual molecules by interacting with the C-domain of the next molecule (Fig. 6b, Supplementary Figs. 10, 11). The N-terminal domain of *ana*Cyp40 (residues 42–162) has a total of five helices, which are all arranged parallel to each other and connected by short loops (Fig. 6a). The first Helix (α1) is about half as long as the other four helices (α2–α5), which form a four-helix bundle. The topology of the four-helix bundle is very similar to the PsbQ proteins from photosystem II of higher plants (e.g., pdb-id: 1vyk, 2mwq, 3ls1; Supplementary Fig. 12)^59–61^ leading to the annotation of this domain as Q-domain (Fig. 6a). However, helix α1 has no counterpart in other known PsbQ structures (Supplementary Fig. 12).

**Fig. 7:**
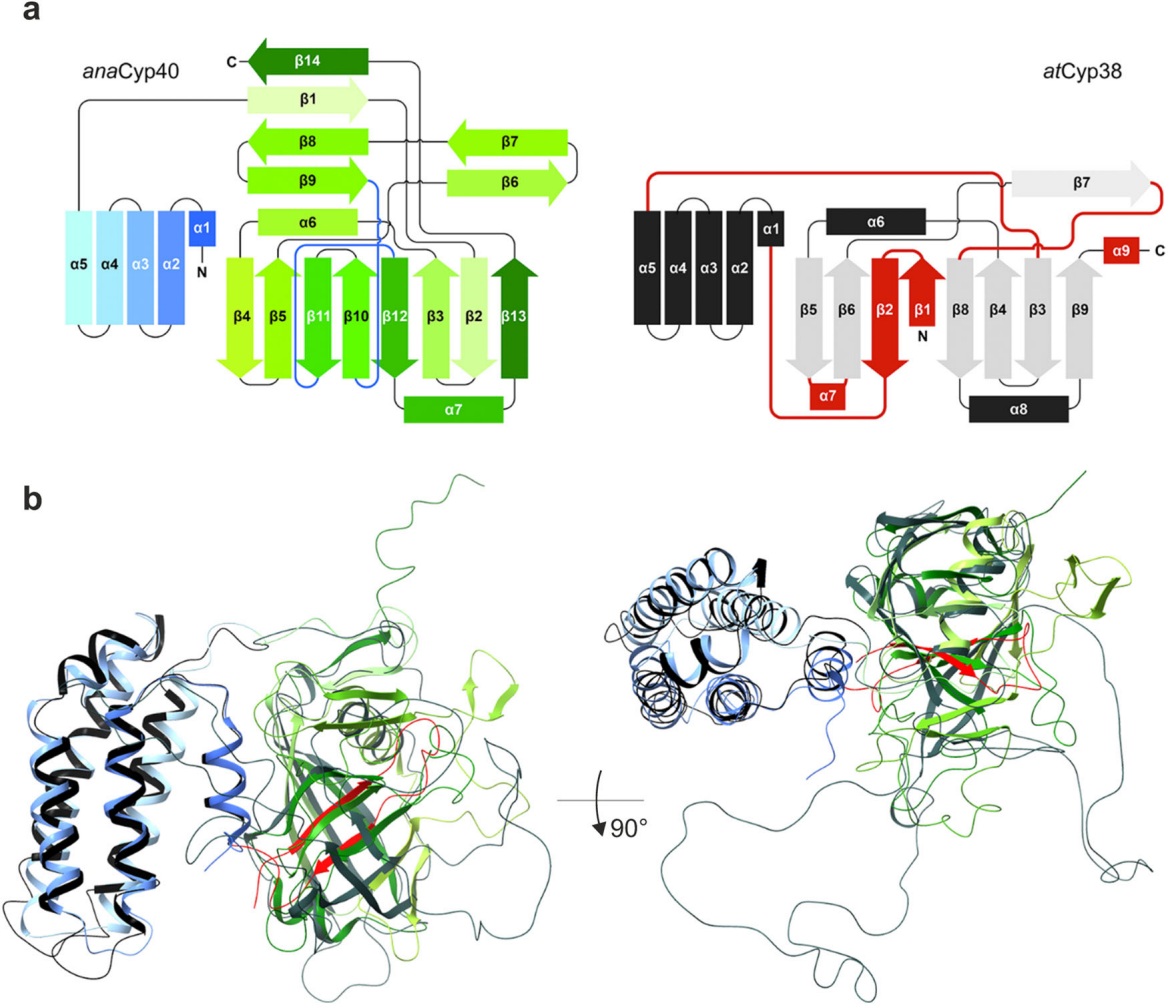
Topological and structural comparison of *ana*Cyp40 and *at*Cyp38. **a** The topological organization of structural elements in *ana*Cyp40 is shown on the left and of *at*Cyp38 (pdb-id: 3rfy) on the right. In *ana*Cyp40 the Q-domain is shown in blue colors and the C domain in red colors. The loop regions interacting with helix α1 are shown as blue lines. β-strands β2- β5 and β10-β13 form the 8-stranded β-barrel. In *at*Cyp38 the structural elements of Q-domain are shown in black and of the C-domain in gray. Strong discrepancies to the topology of *ana*Cyp40 are indicated in red. **b** The structural comparison of *ana*Cyp40 and *at*Cyp38 from two perspectives shows a very similar organization of the Q domain whereas the similarities in the C-domain are restricted to the β-barrel and the helices surrounding the β-barrel. Compared to the unstructured long loops that connect the helices and β-strands of the β-barrel in *at*Cyp38, the loops in *ana*Cyp40 are shorter and have structural elements like β-hairpins and short β-sheets. Coloring of the structural elements in *ana*Cyp40 is according to Fig. 6. The topological discrepancies are highlighted in red also in the structure of *at*Cyp38.

Helix α1, located between the four-helix bundle and cyclophilin domain, is the main structural element that mediates the interaction of the Q- and C-domain. Helix α3 in the bundle is interrupted at about 2/3 of its length, and the shorter part is tilted by about 45° with respect to the rest of that helix and the bundle. The four-helix bundle is held together by interactions of hydrophobic residues that are pointing to the center, forming a hydrophobic core. On the surface ionic and hydrophilic interactions predominate, where the predominantly positively charged surface of helices α2 and α3 stands out (Supplementary Fig. 13). Together with the N-terminal area of helix α1, they form a fairly large positively charged area on that side, while more or less neutral to slightly negatively charged residues are present on the surface of helix α4 and α5 (Fig. 7). The topological arrangement of the four-helix bundle of *ana*Cyp40 is also present in other unrelated proteins, as found by the structural analysis of the Q-domain using DALI^62^. In addition to the PsbQ proteins and among others, cytochrome C (pdb-id: 1CPR; 1QQ3)^63,64^, complement inhibitory domain of BBK32 (pdb-id: 6N1L)^65^, or even the transmembrane subunit of the plasma membrane CO_2_ channel LCI1 (pdb-id: 6BHP)^66^ have the same helix topology and therefore exhibit high structural similarity and low RMSDs (<2 Å) to the *ana*Cyp40 Q-domain (Supplementary Fig. 12).

**Fig. 8:**
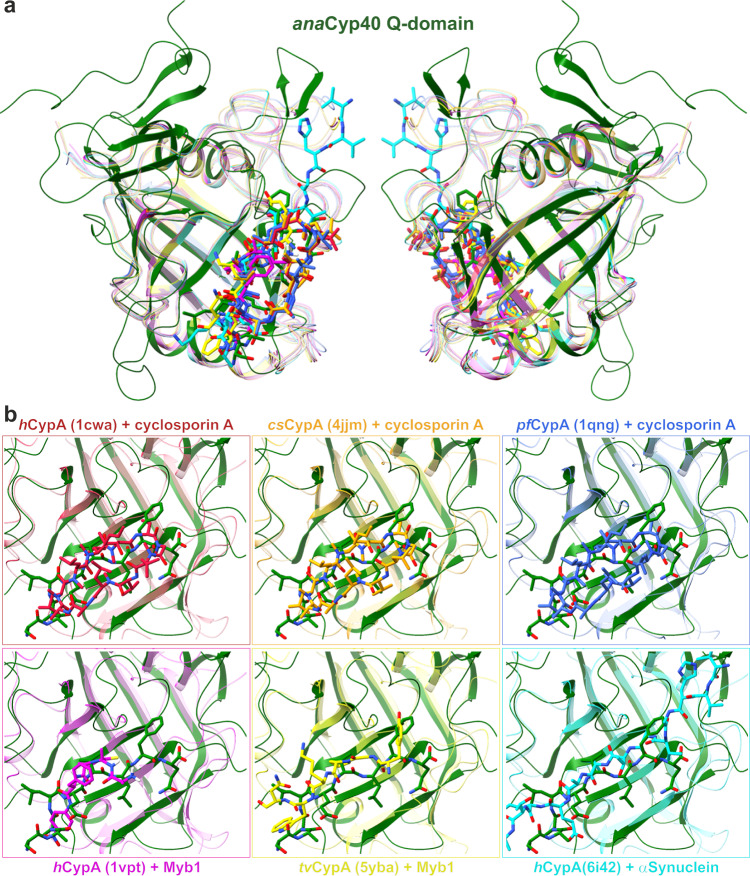
Superimposition of the *ana*Cyp40 C-domain to single-domain cyclophilins. **a** The C-domain of *ana*Cyp40 is superimposed to single-domain cyclophilins identified by DALI^62^ with an RMSD < 2 Å. Three of the compared domains (1cwa:^67^ red; 4jjm:^125^ orange; 1qng:^67^ blue) have a bound cyclosporin A at the corresponding site of *ana*Cyp40, where the C-terminal peptide from the symmetry-related molecule is bound. The other three remaining domains (1vbt:^126^ magenta; 4jjm:^125^ orange; 6i42:^127^ cyan) have at the same position peptides of different length. **b** Individual comparison of the substrate binding sites are highlighted.

The cyclophilin domain (C-domain, residues 163-368) is formed by 14 β-strands, of which 8 antiparallel β-strands form a distorted β-barrel that is flanked by an α-helix on each side (Fig. 6a). Helix α6 between β3 and β4 and helix α7 between β12 and β13 are connected to the β-barrel by short loops. Helix α6 is surrounded by the β-hairpins formed by β6/7 and β8/9, and by a β-sheet formed by the β-strands β1 and β14. In contrast to the short loops connecting the β-strands β2 to β3 and β4 to β5 in the β-barrel, the loops between the other β-strands are relatively long (8–20 amino acids). The Q- and C-domain in *ana*Cyp40 are well separated from each other. In contrast, the Q- and C-domain in *at*Cyp38 are intertwined with β-strands β10 and β11, which are topologically placed to the N-terminus of the protein (Figs. 7; 8a). Consequently, the C-domain topology of *ana*Cyp40 does not agree with that of *at*Cyp38^23^, but it is in perfect topological agreement with all other single-domain cyclophilins with known structures like hCypA^67^ and PfCyP19^68^ (Fig. 8).

*Ana*Cyp40 crystallized in the absence of substrates or inhibitors. However, the C-terminal tail of the second molecule with the leftover residues from PreScission cleavage (amino acids ^370^LEVLFQ^375^) occupies the potential substrate binding pocket (Figs. 6b; 8, Supplementary Fig. 10) and probably mimics a substrate of *ana*Cyp40. The binding of the peptide stretch is mediated by sidechain interactions of Glu226 and Gln231 from β5, Asp234 and Gln240 from the loop downstream to β5, and Gln306 from β11 with the backbone of the “substrate”. Also, hydrophobic interactions of Phe308 and Phe310 from β11 contribute to the binding. In close proximity to the C-terminus of the “substrate” we also identified a metal binding site, formed by the carboxyl group of Asp234 and backbone of oxygens of Asp234, Pro235, Gly237, Glu239. These residues surround the ion, together with three water molecules. The heptagonal coordination geometry of the metal ion and the electron density suggests a Ca^2+^ ion binding at this position (Fig. 6b), which remained bound to the protein during the purification steps, indicating a tight binding of that ion.

## Discussion

*Ana*Cyp40 belongs to the multidomain cyclophilin protein family (TLP40 like) with a predicted transmembrane helix at its N-terminus (aa 13-30), followed by a domain of unknown function (DUF, aa 54–148) and a C-terminal peptidyl-prolyl cis–trans isomerase domain, first identified in spinach (TLP40)^13–15^. These proteins are present in the green lineage ranging from cyanobacteria to algae and higher plants. In contrast, they do not exist, or at least have not been identified so far, in heterotrophic/chemoautotrophic bacteria, fungi or mammals, suggesting a specialized function for photosynthetic organisms (Fig. 1). TLP40 contains two important N-terminal segments (GLKALDSVERN^158^ and AGLAESKKDRG^185^) with similarity to the sequence of FKBP12, which is known to participate in recruiting the protein phosphatase calcineurin (~60% similarity)^69^. These are involved in the regulation of thylakoid protein phosphorylation by TLP40^70^. Interestingly, these sequences are either not present (motif 1, loop between helix α2 and α3; Fig. 6) or replaced in *ana*Cyp40 (motif 2, loop between helix α3 and α4; TSVPEERQTQA^114^; ~10% similarity to the phosphatase binding sequences of FKBP12; Fig. 6), which precludes its possibility to regulate thylakoid protein dephosphorylation. In addition, a sequence alignment revealed differences between the cyanobacterial and the plant protein, particularly in the Q-domain^39^. These two observations might be the reason for an at least in parts different function of the cyanobacterial and the plant protein. Nevertheless, *ana*Cyp40 is required for the regulation of the photosynthetic activity as well, especially under high light conditions (Fig. 3).

The N-terminal *ana*Cyp40 Q-domain, and the homologous domains in *at*Cyp38^23^ share structural similarity to several unrelated proteins. Exemplarily the oxygen evolving subunit of PSII (PsbQ)^59–61^, cytochrome C^63,64^, the complement inhibitory domain of BBK32^65^, or even the transmembrane subunit of the plasma membrane CO_2_ channel LCI1^66^ have high structural similarity to the *ana*Cyp40 Q-domain (Fig. 7), indicating a high versatility for this domain architecture. In contrast to PsbQ (pdb-id 1vyk)^59^ and cytochromes^63,64^, which have specifically bound Zn^2+^ ions (Fig. 7b), metal binding was not identified in the *ana*Cyp40 Q-domain (Fig. 7a). However, the surface charge distribution with positively charged clusters that is present on one side of the Q-domain indicates that the function of this domain is likely to position the catalytic domain with respect to the membrane (Supplementary Fig. 13).

The cyclophilin domain of *ana*Cyp40 is enzymatically active (Fig. 1), as shown previously for TLP40^15^. Consistent with this, the expression of the cyanobacterial gene in *E. coli* increases the resistance of the bacteria against various abiotic stresses. On the other hand, the loss of function of *ana*Cyp40 in *Anabaena* sp. increases the stress sensitivity (Figs. 1 and 2). This phenotype can be explained by a function of *ana*Cyp40 in maintaining the correct protein folding during stress in cooperation with chaperones, as reported for other cyclophilins^1–3^. Indeed, *ana*Cyp40 interacts with proteins of the transcription coupled translation machinery, as well as with 29 proteins that contain a high percentage of prolines, which are likely substrates of *ana*Cyp40 (Table 1).

**Fig. 9:**
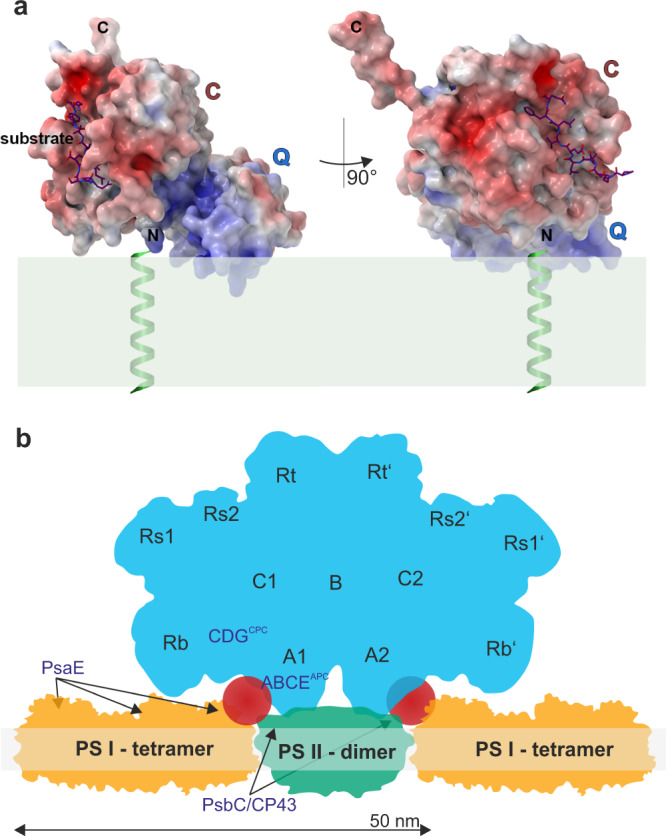
Model of *ana*Cyp40 at the membrane and hypothetical functional models. **a** *Ana*Cyp40_ΔTM_ is placed on the membrane (green) in that way that the positively charged surface of the Q-domain interacts with the membrane lipids. In this orientation the N-terminus is close at the membrane, so that the trans membrane helix in the full-length *ana*Cyp40 can be attached to the N-terminus of *ana*Cyp40_ΔTM._ In this orientation also the substrate binding site is accessible for the substrate of *ana*Cyp40. **b** The structure of the tetrameric PSI^71^, the dimeric PSII^72, 73^ and the PBS^74^ as well as the information on the positioning of the PBS with respect to PSII^74^ was used to create a model for the positioning on/in the thylakoid membrane. The different domains of the PBS (black letter) and the positioning of the proteins found to complex with *ana*Cyp40 (purple letter) are indicated. The red circles indicate the maximal dimension of the soluble domain of *ana*Cyp40 that would fit into the cavity in the PBS between PSII and PSI according to the first hypothetical functional model. Note: PBS-PSII-PSI megacomplex formation used here to illustrate a putative function of *ana*Cyp40 is only one mode of complex organization at membranes as current results point toward a flexible arrangement. Hence, reader should keep in mind that this is only one possible ensemble.

The structure presented here is consistent with the observed enzymatic property of the *ana*Cyp40 C-domain, which interacts with the flexible C-terminus of another molecule that represents an unfolded or not properly folded amino acid stretch. The substrate binding pocket is similar to other biochemically active single domain cyclophilins (Figs. 6–9) with one exception. In the reported structure for *at*Cyp38, two β-strands, which correspond to the β-strands β10 and β11 in the *ana*Cyp40 β-barrel, were placed topologically N-terminal to the *at*Cyp38 Q-domain (Fig. 7). These served as indication for the inactivity of *at*Cyp38, as this “β-sheet insertion” would prevent substrate binding to the active site. However, as *at*Cyp38 residues 78-83, which are located in the N-terminal half of the first β-strand, originate from the thrombin cleavage site of the construct used for the crystallization, we assume an incorrect assignment and subsequent topological misinterpretation of the *at*Cyp38 structure. In addition to the topological mismatch found in the N-terminal β-strands, direct structural comparison of *ana*Cyp40 and *at*Cyp38 shows more discrepancies. While in *ana*Cyp40 the conserved canonical cyclophilin-like domain that comprises two α-helices (amino acids 201–214 and 337–344) flanks the distorted β-barrel with eight β-strands in a (β2-3)-α6-(β4,5,10-12)-α7-β13 architecture (Figs. 6, 7), the loops between the β-strand and α-helices of the *at*Cyp38 C-domain are remarkably long. The analysis of the electron density and the available *at*Cyp38 model deposited in the PDB (pdb-id: 3rfy)^23^ gives the explanation for this. For most of the residues in these long loops, no electron density is present. Moreover, a metal binding site where Ca^2+^ was bound is present in *ana*Cyp40 in close proximity to the C-terminus of the “substrate”. This might represent a specific regulatory mode for substrate recognition. Metal binding close to the cyclophilin domain is reported so far only for the phloem cyclophilin BnCYP19-1^71^.

In vivo multi-color super-resolution microscopy revealed the structural organization of *Ana*Cyp40 directly in cells. We have developed a simple and robust procedure to visualize *Ana*Cyp40 in respect to the thylakoid and cellular membrane with a spatial resolution of 24 nm. Imaging data showed that *Ana*Cyp40 is localized in, or at least attached to the thylakoid membrane (Figs. 5, 9), where the protein interacts with components of the PBS and the photosystems (Fig. 4, Table 1). These results support the findings obtained in this study on the association of *Ana*Cyp40 to PBS and the photosystems in a cellular context. This interaction appears to be specific and is not dependent on the expression level or a high proline content of the interaction partner (Table 1; Supplementary Table 4). Analyzing the position of the interaction partners in existing structures of tetrameric PSI^52^, dimeric PSII^72,73^ and phycobilisomes^74–76^ shows an enrichment of those between the two photosystems and near a cavity in the PBS between the core (A1/A2) and the Rb rods. Remarkably, the cavity has a dimension of about 6–7 nm.

One model based on the obtained results would place *ana*Cyp40 as part of the assembled structure between PBS and the photosystems (Fig. 9; green circle). In line with such interpretation, PsaE exposes a domain toward the cytoplasm^52^ while PsbC is a transmembrane protein facing the outer rim of PSII^72,74^. The other two identified PSII proteins, PsbO and PsbP (CyanoP) directly interact with PsbC^77,78^. Both proteins are present in the thylakoid lumen and thus either they interact with *ana*Cyp40 through its lumenal exposed N-terminus, or the three PSII proteins together with *ana*Cyp40 form a subcomplex that remains stable during the solubilization of the thylakoid membrane. Worth mentioning, PsbP is discussed to be important for PSII assembly^79–81^. However, we did not observe a significant reduction of PSII monomer or dimer, only of the PSI dimer (Fig. 4). Thus, this model suggests that the membrane-anchored *ana*Cyp40 is required for the arrangement or stabilization of the photosynthetic super complex involving PBS, PSI and PSII. Consistent with such hypothesis, AFS-I-*anacyp40* is sensitive to high light, which was reflected by a reduced growth and a reduction of the oxygen evolution rate and the maximal effective PSII quantum yield in the mutant strains (Figs. 2, 3). Moreover, a lower fluorescence emission from APC and PSII after excitation with 580 nm light was observed in the mutants under high light, which suggests a disturbed PBS function. A similar loss of APC activity was observed for a mutant of *Synechocystis* sp. PCC 6803, which cannot assemble functional phycobilisomes^82^. Consequently, our results are indicative for a reduced energy transfer between phycobilisomes and the photosystems in general, as the transfer between PBS and PSI or PSII is comparable between wild type and the mutant strain. The phycocyanin fluorescence after excitation of chlorophyll was enhanced in the mutant compared to wild-type when grown under high light, this can be linked to the previously discussed assembly of PBS with IsiA^83^, which is induced under high light conditions^84^. Again, the increased association of PBS with IsiA in the mutant supports the hypothesis of a destabilized assembly of PBS with the photosystems. The absence of PSI complex dimers and the reduction of tetramers observed in the mutants (Fig. 4a), the latter is likely required for PSI-PBS connectivity^82^, had a considerable negative effect on energy transfer from the PBS.

An alternative model could be built on the cyclophilin activity of *ana*Cyp40. The peptidyl-prolyl cis-trans isomerase activity of the membrane anchored protein could be important for the proper folding of the PBS and PS proteins, as prerequisite of the subsequent assembly. Remarkably, 9.3% of all amino acids of PsaL are prolines and although not detected in our experiment, this protein could be a prominent target. Its malfunction would lead to reorganization of the oligomeric state of the PSI^52,53^. This model would define a functional relevance of *ana*Cyp40 comparable to that of HspA, which is involved in the salt stress response^85^ and the protection of the photosynthetic machinery^86–88^. Such functional model for *ana*Cyp40 would still be consistent with the membrane localization observed, because thylakoid associated ribosomes have been observed which would link translation and protein folding in the vicinity of these membranes^89^. However, based on electron tomography, membrane associated ribosomes and the PBS-PS complexes do not co-localize^89^, which leaves the association of *ana*Cyp40 with specific PBS and PS proteins unexplained.

Summarizing, the cyclophilin *ana*Cyp40 from the cyanobacterium *Anabaena* sp. is an enzyme which might have a dual functionality. One the one hand, the structural composition in conjunction with the functional properties denote the protein as factor for the general stress response, due to its proposed activity in general protein folding. On the other hand, its association with the thylakoid membrane (Fig. 9a), with the photosystems and PBS (Fig. 9b), in combination with the physiological properties of the mutant strain mark *ana*Cyp40 as an important factor for the regulation of cyanobacterial photosynthesis. The exact mode of this regulatory function can only be speculated on at stage, and further investigation is required to provide a detailed explanation. Moreover, its relation to the dimerization of PSI and PSII and thus, to PsaL, has to be further resolved. The discrepancy to the plant proteins is in accordance with the fact that cyanobacterial D1 is not phosphorylated^24^, while plants do no longer contain PBS^82^. Thus, the function of plant TLP40 has likely been adjusted during evolution. Moreover, our structural analysis places the TLP40 family into the family of typical cyclophilins. Hence, *ana*Cyp40 likely provides a link between photosynthetic performance and stress response. Whether these two functions co-exist, or whether *ana*Cyp40 switches between the proposed two functions during stress remains to be established.

## Methods

### Bioinformatics analysis

*Anabaena* sp. Cyp40 orthologs were identified from OMA orthology database^90^ using default settings. Putative functional domains were searched using the Conserved Domain Database (CDD)^91^. Multiple sequence alignments were computed using MUSCLE and phylogeny was inferred by using Maximum Likelihood method with default settings of MEGA6^92^. Branches corresponding to partitions reproduced in less than 60% bootstrap replicates are collapsed. Initial tree(s) for the heuristic search were obtained by applying the Neighbor-Joining method to a matrix of pairwise distances estimated using a JTT model. A discrete Gamma distribution was used to model evolutionary rate differences among sites (5 categories (+G, parameter = 2.4832)). The analysis involved 72 amino acid sequences. All positions with less than 95% site coverage were eliminated. That is, fewer than 5% alignment gaps, missing data, and ambiguous bases were allowed at any position. There was a total of 153 positions in the final dataset. List of genes used for phylogenetic analysis is present in Supplementary Table 1. Prediction of signal peptide is described in the text and of transmembrane domains in Supplementary Table 2.

### Stress tolerance assay utilizing *E. coli*

*ana*cyp40 was cloned into pET21a followed by transformation of pET21a—*ana*cyp40 and pET21a into *E. coli* strain BL21 (DE3). Primers used are listed in Supplementary Table 8. The impact of abiotic stresses on *E. coli* BL21(DE3) strains transformed with empty pET21a and pET21a—*ana*cyp40 was determined by spot assay after subjecting *E. coli* BL21 cells to different treatments of arsenic (Na_3_AsO_4_), heat, salinity (NaCl) and UV-B. Prior to stress treatment, transformed cells were grown in LB medium with 100 μg/ml ampicillin at 37 °C up to OD_600nm_ = 0.6, followed by IPTG addition (final concentration 0.3 mM) and growth for 5 h at 16 °C. Cultures were serially diluted (10^−1^, 10^−2^, and 10^−3^ fold) and 3 μl from each dilution was spotted on LB-ampicillin plates comprising indicated concentrations Na_3_AsO_4_ or NaCl. For heat (50 °C) and UV-B treatment (12.9 mW m^−2^ nm^−1^), induced *E. coli* cells were exposed for indicated times followed by spotting on LB ampicillin plates. All plates were incubated at 37 °C overnight and images were captured.

### Enzymatic analysis of PPIase activity

PPIase activity was examined at 15 °C for indicated times in a coupled reaction with chymotrypsin^40^. The 1 ml mixture contained 40 μM N-succinyl-ala-ala-pro-phe p-nitroanilidine in 50 mM HEPES (pH 8.0), 150 mM NaCl, 0.05% Triton X-100 and 5 nM of protein. The reaction was started by the addition of chymotrypsin (300 μg/ml) at 15 °C and the change in absorbance at 390 nm was recorded, the number of replicas is indicated in the figure legend.

### Cyanobacterial and bacterial strains and plasmids generation

All strains used are listed in Supplementary Table 4. For the generation of *ana*cyp40 single-recombinant insertion mutants (AFS-I-*anacyp40*), an internal fragment (168–665 bp) of the coding region was amplified by PCR from *Anabaena* sp. genomic DNA (oligonucleotides: Supplementary Table 8) introducing BamH1 restriction sites. PCR product was cloned into pGEM-T vector (Promega), producing plasmid pGEMT-I-*ana*cyp40 (bp168–665) and further subcloned in pCSV3 vector^93^ containing an Sp^r^ Sm^r^ gene cassette, resulting in plasmid pCSV3-I-*ana*cyp40 (Supplementary Table 9). In addition, a plasmid with a gene coding for a translational fusion of *ana*Cyp40 with the C-terminal Strep-tag (WSHPQFEK) was constructed. The gene and Strep-tag were amplified by PCR (oligonucleotides: Supplementary Table 8) and cloned into the EcoRI sites into pGEM-T-Easy vector (Promega), producing plasmid pGEMT-Easy-*ana*cyp40-strep. The product was cloned in pCSV3 vector^93^ containing an Sp^r^/Sm^r^ gene cassette, resulting in plasmid pCSV3-*ana*cyp40-strep (Supplementary Table 9). The plasmid was transferred into wild-type *Anabaena* sp. through conjugation^94^ producing single recombination mutant AFS-*anacyp40*-strep. The fusion was confirmed by colony PCR.

### Transcript analysis

RNA extraction using the RNASure mini kit (Nucleo-pore) was performed from 50 mL culture (OD_750nm_ = 0.6) of *Anabaena* sp. grown in BG11 before and after 1 day of incubation in 100 mM NaCl, 40 mM Na_3_AsO_4_, exposure to 42 °C for 1 h, 30 °C for 10 h or 12.9 mWm^−2^ nm^−1^ UV-B for 30 min^21,22,95–97^. Total RNA (1 µg) was reverse transcribed in a 20 μl using the iScript cDNA synthesis kit (BioRad). Transcript analysis was performed using gene specific primer for *ana*cyp40 and for reference 16S rRNA (Supplementary Table 8). For qRT-PCR using a CFX-96 (Bio-Rad), 15 ng of cDNA extracted from each sample was used in 20 μl including 10 pmol of each, forward and reverse primers and 1x Sso fast evagreen qPCR supermix (BioRad). Transcript levels were normalized to 16S transcript and calculated relative to 0 h using the 2^−ΔΔCt^ method^98^ to evaluate the relative quantities of each amplified product. The threshold cycle (Ct) was automatically determined for each reaction by the system (default parameters). The specificity of the PCR was determined by melting curve analysis of the amplified products.

### *Anabaena* sp. growth, spotting assay and chlorophyll content estimation

*Anabaena sp*. was grown in BG11 medium^99^ under photoautotrophic conditions buffered with 8 mM TES-NaOH, pH 7.5 and 17.6 mM NaNO_3_ as the nitrogen source at 30 ± 2 °C under constant illumination (70 μmol photon m^−2^ s^−1^, photosynthetically active radiation). Cultures of AFS-I-*anacyp40* and AFS-*anacyp40*-strep contained 3 μg ml^−1^ streptomycin/3 μg ml^−1^ spectinomycin. Agar plates were prepared with 1% Bacto™ Agar (Otto Nord Wald, G).

For growth curve analysis, cells were grown in BG11 liquid medium, washed three times by centrifugation (5000 × *g*, RT, 3 min) and resuspended in BG11 adjusted to an OD_750_ of 0.05. All experiments were performed in triplicates in 50 ml culture volumes. Alternative additions ore treatments are described in the text. OD_750_ values were recorded once a day for 8 days. For growth analysis on solid medium, cells were spotted in triplicates at different OD_750_ (0.25, 1.0) on standard BG11 agar medium either in the presence or absence of the indicated amounts of NaCl concentrations. Chlorophyll a concentration was determined^41^.

### 77 K Fluorescence emission spectra

77 K fluorescence measurements were performed using an JASCO spectrofluorimeter (FP-8700 spectrofluorimeter, JASCO). Cuvettes were kept immersed in liquid nitrogen and transferred to the measuring cell of the fluorimeter one at a time. Chla excitation was performed at 440 nm and PBS excitation at 590 nm. Fluorescence emission was measured from 600 to 800 nm. Assignment of peaks was made according to literature^47–50^.

### Chlorophyll fluorescence analysis and oxygen evolution measurements

Chlorophyll fluorescence analysis were acquired using a Closed FluorCam FC 800-C (Photon System Instruments, Brno, Czech Republic) with a CCD camera and four fixed LED panels. All images were captured using the FLUORCAM 7 software (Photon System Instruments). Chl a fluorescence images were taken using the imaging PAM fluorimeter^100^. In brief, cells in 2 ml of liquid culture were dark-adapted for 15 min and the minimum fluorescence level (F_o_) was determined with a measuring light. After start of the measurement, a 0.8 s pulse of saturating light was applied (determination of F_m_′_dark_) followed by exposure to actinic light for 300 s with saturating pulses in between (determination of F_m_′). The actinic light was then turned off and saturating light was applied to monitor recovery kinetics of F_m_′ in the absence of actinic light. Fluorescence parameters were determined by established protocols^101^.

Oxygen evolution was quantified in 2 mL culture samples (adjusted to 20 μg chlorophyll mL^−1^) in BG11 with 10 mM NaHCO_3_ using a Clark-type electrode and DW2 Oxygen Electrode Chamber (Hansatech) with constant stirring at 30 °C. Light was introduced to samples using an LS2/H tungsten-halogen 100 W light source. All measurements were performed in triplicate.

### Thylakoid membrane preparation and BN-PAGE

Isolated thylakoid membranes^102^ equivalent to 1 mg Chl ml^–1^ was solubilized with 1% n-Dodecyl β-D-maltoside (β-DM) on ice for 15 min, followed by centrifugation at 9391 × *g* rpm for 10 min at 4 °C. The supernatant (equivalent to 7 μg of Chl) was subjected to BN-PAGE^103^ with a gradient of 5–13.5% (w/v) acrylamide in the separation gel. Complex assignments were performed according to existing data in the lab based on previous reports^104^.

### Phycobilisome (PBS) Isolation

PBS was isolated according to established protocols^105^ with few modifications. 500 ml cells grown in BG11 were harvested, washed twice with 0.9 M potassium phosphate buffer (pH 7.0), and crushed in liquid nitrogen thrice. The cell extract was treated with 2% (vol/vol) Triton X-100 in 0.9 M phosphate buffer for 30 min and centrifuged at 20,000 × *g* for 20 min at 18 °C to separate the extract into the upper green Triton X-100 layer and the lower blue aqueous layer. The blue layer was loaded onto a 10–50% (wt/vol) linear sucrose density gradient with 0.9 M phosphate buffer and centrifuged at 130,000 × *g* for 16 h at 18 °C.

### Affinity Purification of *ana*Cyp40 Complexes

500 ml culture (OD ~ 3.0) of AFS-*anacyp40*-strep or wild-type *Anabaena* sp. was centrifuged at 8000 rpm for 10 min. Cell pellets were resuspended in 15 ml of ice-cold buffer (100 mM Tris, pH 8.0, 50 mM NaCl, 5 mM β-mercaptoethanol, 1 mM PMSF). Following three cycles of lysis in liquid nitrogen, 1 mg/ml lysozyme, 1% β-DM and Protease inhibitor cocktail was added. After incubation for 60 min at 4 °C on rotary shaker, the whole cell lysate was centrifuged at 3500 rpm for 2 min and biotin blocking buffer was added. This fraction was loaded onto MagStrep “type3” XT beads (IBA Life Sciences) preequilibrated with lysis buffer and left on rotary shaker for 60 min at 4 °C. The column was washed with 100 mM Tris, pH 8.0, 1 mM β-mercaptoethanol, 1 mM PMSF, 1 mM EDTA containing 50 mM NaCl (WB1), 100 mM NaCl (WB2) or 100 mM NaCl and 0.5% Nonidet P-40 (WB3). *ana*Cyp40 protein complexes were eluted WB1 containing 50 mM D-biotin. Protein abundance was tracked using Strep-Tactin﻿® HRP conjugate (IBA Life Sciences).

### Tryptic *in solution* digestion and liquid chromatography electrospray ionization MS

Elutions form affinity purification were pooled and and 2X Lysis Buffer (5% DCA, 5 mM TCEP, 100 mM NH_4_HCO_3_) was added, mixed at ~1000 rpm in a thermo-mixer for 10 min at 90 °C. Subsequent alkylation was performed (15 mM IAA, 3 mM NH_4_HCO_3_, pH 8) and samples were kept in the dark for 30 min followed by dilution in dilution buffer up to final concentration of 0.5% DCA, 0.5 mM TCEP, 100 mM NH_4_HCO_3_. Trypsin was added to the samples (1:500 w/w) and incubated at 37 °C for overnight. Reaction was stopped by addition of 1.0% TFA. The samples were desalted using C18 column^106^. Tryptic peptides recovered from C18 column were fractionated using strong cation-exchange (SCX) StageTips^107^. In total, 4 SCX fractions were collected by eluting first 3 fractions in 75, 200, 400 NH_4_HCO_3_/30% ACN/0.5% TFA solutions followed by a final elution with 5% mM ammonium hydroxide/50% ACN. Fractions eluted were dried in a SpeedVac, resuspended in ddH_2_O and subjected to LC-MS analysis as described^55^.

The three experiments (experiment 1: one biological probe and 12 controls, experiment 2 and 3: three biological replicas and three controls) were analyzed to determine the LFQ value for each experiment. For further analysis, only proteins with identified peptides in all bait-dependent precipitations and an LFQ > 0 in at least 2 of the bait containing probes were considered. The ratio of log_10_(LFQ + 1)_BAIT_-log_10_(LFQ + 1)_CONTROL_ was calculated and the *p*-value for the distribution of the ratios was determined for the hypothesis of a median ≤0 by one way *t*-test (Sigma Plot, SPSS).

### Super-resolution imaging

Fixation of cells was performed according to existing protocols^108^. In brief, 20 µl of cyanobacterial culture (OD_750_ = 0.3) was added to poly-lysine coated surfaces and dried for 20 min at 55 °C followed by fixation in 70% ethanol and incubation for 30 min at −20 °C. The surfaces were air-dried for 20 min at room temperature and permeabilized by adding a drop of 0.05% Triton X-100 in PBS for 2 min at room temperature, and repeated it three times by removing the drop each time with a pipette. Afterward, incubated with a drop of 3% BSA (bovine serum albumin), 0.2% Triton X-100 in PBS for 1 h at 4 °C in a moisture chamber and removed the blocking solution. Cells were incubated with anti-strep primary antibody for 2 h at 4 °C in a moisture chamber and subsequently washed three times at room temperature. Afterward, incubated with oligolabelled secondary antibody for 1 h at 4 °C in a moisture chamber followed by three washing steps. The docking strand (DNA strand at the secondary antibody) was P1.

Super-resolution imaging was performed on a home-built microscope as described^109^. The software μManager^110^ was used to control the microscope hardware and the Andor Ixon Ultra EMCCD camera (DU-897U-CS0-#BV; Andor). Measurements were performed applying a highly inclined and laminated optical sheet^111^. First, samples were illuminated with a 647 nm excitation laser to detect the autofluorescence until all cellular fluorophores were bleached. Typically, 60 min of imaging data were recorded at a frame rate of 20 Hz (72000 frames). Second, 400 pM P1-Atto655 imager strand in PBS supplemented with 500 mM NaCl was added to the sample and excited at 647 nm using the same laser. 48,000 frames with a frame rate of 20 Hz were recorded to visualize the P1 labeled prolylisomerase. Third, 500 pM NileRed in PBS was added to the sample to visualize the outer and plasma membrane^112^, illuminated with a 568 nm excitation laser and 15,000 frames with a frame rate of 50 Hz were recorded. Laser densities range between 0.5 and 5 kW/cm^2^.

Super-resolution images were generated using rapi*d*STORM^113^ or Picasso^114^ and post-processed using Fiji^100^. Single-molecule localizations were fitted using free PSF fit parameters in rapi*d*STORM. For autofluorescence and NileRed, a global threshold of 132 photons was applied, and for Atto655, a global threshold of 1320 photons was used. Localizations were filtered by their full width at half maximum (220 nm < FWHM < 440 nm for the autofluorescence and Atto655, 280 nm < FWHM < 360 nm for NileRed) and PSF asymmetry (0.7 < FWHM(x)/FWHM(y) < 1.3). Brightness and contrast were adjusted for optimal visualization. The software Origin2019 (OriginLab Corporation) was used for calculations and graphical data representation. Parameter-free image resolution estimation based on decorrelation analysis was performed using the Fiji plugin ImageDecorrelationAnalysis^57^.

Cluster analysis was performed using the DBSCAN algorithm implemented in the LAMA software^115^. Localizations with equal or more than 10 neighbors (minpts = 10) within a 20 nm radius were assigned to a cluster.

### Protein Purification for crystallization

The 10x-His fusion proteins (F7XC3H-*anacyp40*_ΔTM(1-35)_-HRV 3C-His_10_ construct) was overexpressed in *E. coli* BL21 (DE3). Cells were grown in Luria-Bertani medium to an OD_600_ of 0.5–0.7 at 37 °C. Expression of the recombinant protein was induced with 1.0 mM isopropylthio-β-galactoside at 25 °C for 16 h. Cells were harvested by centrifugation (4 °C; 6000 × *g*; 10 min). The bacterial pellet was suspended in ice-cold lysis buffer (20 mM Tris-HCl pH 7.0, 150 mM NaCl), lysed by French press (Thermo Scientific, Waltham, USA) at 1200 psi followed by centrifugation (30.000 × *g*, 4 °C, 30 min). The supernatant was loaded onto Ni-NTA affinity resin (Qiagen, Hilden, Germany), pre-equilibrated with lysis buffer and washed trice with lysis buffer containing 40 mM imidazole to remove contaminants. On-column cleavage of histidine tag was performed using PreScission protease^116^ in 50 mM Tris-HCl pH 7.0, 150 mM NaCl, 1 mM EDTA and 1 mM DTT at an enzyme: protein ratio of 1:50. The cleaved protein has additional seven C-terminal residues. The flow through was collected and cleared by size exclusion chromatography (Superdex 200, GE Healthcare, Solingen, Germany) pre-equilibrated in lysis buffer and subsequently concentrated using amicon ultra centrifugal filters (30 kDa molecular-weight cutoff filter, Merck, Darmstadt, G) up to ~15 mg/ml.

### Crystallization, data collection, and structure determination

Single crystals of *ana*Cyp40_ΔTM_ were obtained after 7 days from drops consisting of 0.2 M ammonium citrate, 20% PEG 3350, and 10% Morpheus additive screen (PEG 3350 precipitant 25.00 % w/v)^39^. Crystals were immersed for 5 min at 293 K in cryoprotectant solution consisting of 20% Glycerol and 18% sucrose followed by flash-cooling in liquid nitrogen. A total of 7600 X-ray diffraction images were collected on the id29 beamline at the European Synchrotron Radiation Facility (ESRF, France) at 100 K in a nitrogen-gas stream and at a wavelength of 0.9795 Å. The data were indexed, integrated and scaled with the XDS program package^117^ and the structure was solved by molecular replacement^118^ using PHASER^119^ from the CCP4 program suite^120^ with the structure of *at*Cyp38 (pdb-id 3RFY) as search model^23^. The high-resolution model was refined by iterative cycles of manual rebuilding using COOT^121^ and phenix.refine^122^ Figures were drawn using ChimeraX^123^. Electronic potentials for surface presentation were calculated using PDB2PQR^124^ and APBS.

### Accession numbers

Sequence data from this article can be found in the UniProtKB under identifier Q8YM80. The coordinates and structure factors for *ana*Cyp40_ΔTM_ have been deposited at the Protein Data Bank under the entry code 7A73.

### Reporting summary

Further information on research design is available in the Nature Research Reporting Summary linked to this article.

## Supplementary information

Supplementary Information

Reporting Summary

Description of Additional Supplementary Files

SUPPLEMENTARY DATA 1

SUPPLEMENTARY DATA 2

## Data Availability

The atomic coordinates for *ana*Cyp40_ΔTM_ have been deposited in the Protein Data Bank with the accession code 7A73. The source data underlying Figs. 1, 2, 3, 5 are provided as a Source data file. Other data that support the findings of this study are available upon request to the corresponding author. Source data are provided with this paper.

## Source data

Source data
