# Supplementary material for: Cyclophilin anaCyp40 regulates photosystem assembly and phycobilisome association in a cyanobacterium
Source: Nat Commun. 2022 Mar 30;13:1690. doi: 10.1038/s41467-022-29211-w (PMC8967839; doi:10.1038/s41467-022-29211-w)
Supplement: Supplementary file 1 — Supplementary Information [file 41467_2022_29211_MOESM1_ESM.pdf]

SUPPLEMENTARY INFORMATION TO THE MANUSCRIPT: "Cyclophilin anaCyp40 regulates photosystem assembly and phycobilisome association in a cyanobacterium" of Shivam Yadav, Martin Centola, Mathilda Glaesmann, Denys Pogoryelov, Roman Ladig, Mike Heilemann, L.C. Rai, Özkan Yildiz and Enrico Schleiff

SUPPLEMENTARY FIGURES

Supplementary Fig. 1: Kinetics of the activity of *ana*Cyp40ΔTM-His.

Supplementary Fig.2: The generation of the AFS-I-*anacyp40* mutant and antisense RNA transcription.

Supplementary Fig. 3: A second example for the BN-PAGE shown in Fig. 4a.

Supplementary Fig. 4: The generation of the AFS-*anacyp40*-strep mutant.

Supplementary Fig. 5: The wild-type and AFS-*anacyp40*-strep response to high NaCl.

Supplementary Fig. 6: Full blot for Figure 4d.

Supplementary Fig. 7: Full blot for Figure 4e.

Supplementary Fig. 8: Estimated resolution of the super-resolution microscopy images.

Supplementary Fig. 9: Super-resolution microscopy images of the wild type control.

Supplementary Fig. 10: The interactions of monomers in the *ana*Cyp40 crystal.

Supplementary Fig. 11: Crystal contacts between the C-terminus of one *ana*Cyp40 molecule with C-domain of a second molecule

Supplementary Fig. 12: Comparison of *ana*Cyp40 Q-domain to structurally related proteins.

Supplementary Fig. 13: The electrostatic surface potential distribution of *ana*Cyp40

SUPPLEMENTARY TABLES

Supplementary Table 1: List of genes used for phylogenetic analysis.

Supplementary Table 2: Overview on transmembrane domain prediction.

Supplementary Table 3: Kinetic parameters of cyclophilins from different species.

Supplementary Table 4: List of Anabaena strains used in this study

Supplementary Table 5: PBS composition.

Supplementary Table 6: Photosynthetic parameter of wild-type and AFS-I-anacyp40.

Supplementary Table 7: Data collection and refinement statistics

Supplementary Table 8: Oligonucleotides used in this study.

Supplementary Table 9: Plasmids used in this study.

REFERENCES IN SUPPLEMENTARY MATERIALS ONLY

SUPPLEMENTARY DATA

Supplementary Data to Figure 1, 2, 3, 5 and Supplementary Figure 1

SUPPLEMENTAL FIGURES

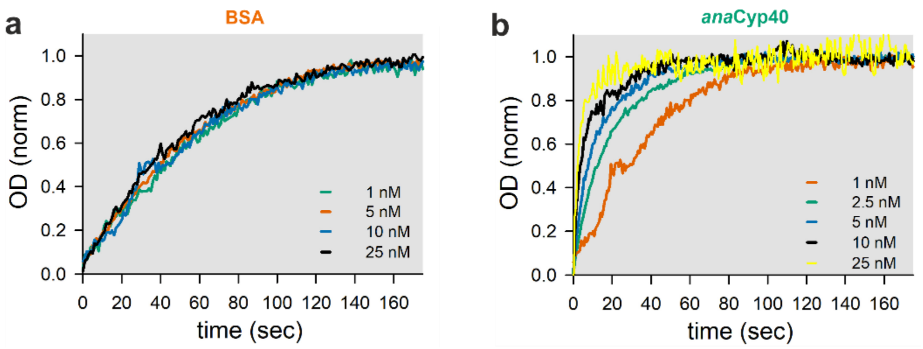

Supplementary Fig. 1: Kinetics of the activity of *ana*Cyp40$_{\Delta TM}$-His.

Purified BSA (**a**) or *ana*Cyp40$_{\Delta TM}$-His (**b**) at indicated concentrations were incubated with 40 µM N-succinyl-ala-ala-pro-phe-p-nitroanilidine and the catalytic reaction monitored by the increase of absorption at 390 nm. The values were normalized to the baseline and to the maximum. A representative experiment is shown.

SUPPLEMENTAL FIGURES

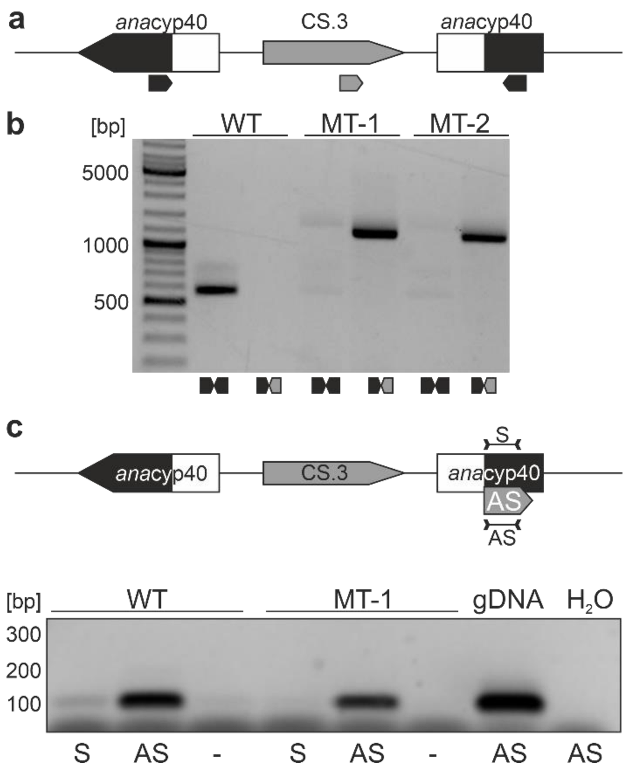

Supplementary Fig. 2: The AFS-I-*anacyp40* mutant and antisense RNA transcription.

a The model for the generated mutant indicating the position of the insertion of the plasmid and the place of annealing of used oligonucleotides is shown**. b** The segregation of the mutant was analyzed with gene and plasmid specific oligonucleotides (Supplementary Table 7) on genomic DNA isolated from wild-type and from AFS-I-*anacyp40*. The experiment was periodically repeated to confirm the segregation throughout the study. **c**. On top, a model of the genomic organization of the mutant strain indicating the position of the antisense region. The produced PCR products are indicated. For the analysis of the RNA presence, the RNA extraction was performed as described using TRIzol (Thermo Fisher Scientific)[1,2]. RNA was isolated from wild type (WT) and AFS-I-*anacyp40* (MT-1) after 24 h of growth in $BG11_0$ (BG11 without nitrate) where the antisense RNA transcript was induced[3]. The absence of DNA in the RNA samples was tested by PCR (not shown). For specific first strand cDNA synthesis 200 ng of RNA were used as template. Revert Aid Transcriptase was utilized (Thermo Fisher Scientific) according to manufacturer's protocol, specific oligonucleotides for the sense (S) and the antisense (AS) transcript were used (0.2 µM) for priming DNA synthesis. To control the RNA self-priming, a control was included where oligonucleotides were omitted (-)[4]. The efficiency of the oligonucleotides used for probing the antisense RNA was tested on gDNA, and the absence of self-annealing by PCR in the absence of RNA or DNA (H_2O). The analysis was performed on three independent cultures. In (b) and (c) the base-pair number for the migration of the molecular main marker is indicated.

SUPPLEMENTAL FIGURES

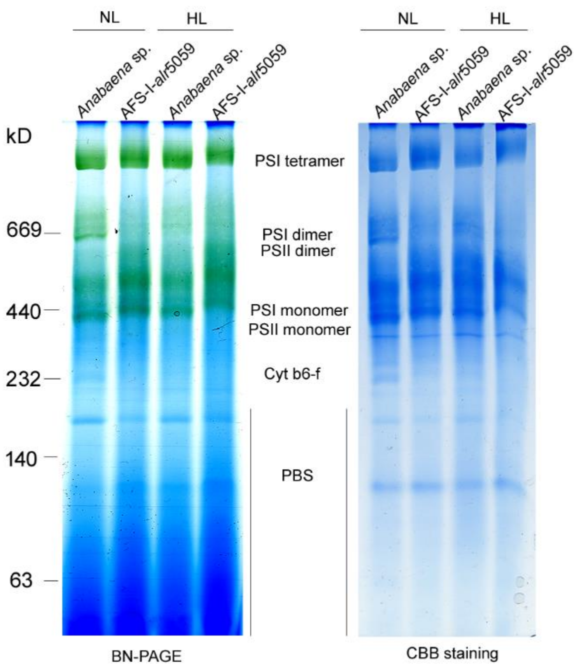

Supplementary Fig. 3: A second example for the BN-PAGE shown in Fig. 4a.

a Membranes of wild-type (green) and AFS-I-anaCyp40 (orange) grown in BG11 liquid medium at 40 or 120 µE illumination were solubilized and subjected to BN-PAGE. The chlorophyll staining (left) and the Coomassie Blue staining (right) are shown. The Coomassie Blue staining was quantified by ImageJ and the intensity of the individual complexes was normalized to the intensity in wild-type grown at 40 µE light intensity. A representative example of three independent trials is shown. The migration of the molecular weight standards is indicated on the left and the molecular weight is given in kDa.

SUPPLEMENTAL FIGURES

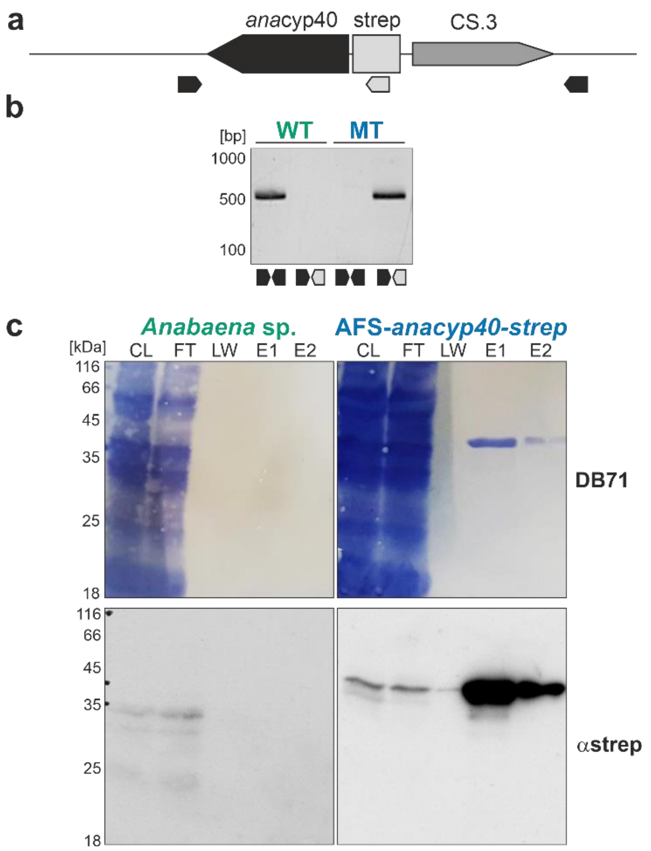

Supplementary Fig. 4: The generation of the AFS-*anacyp40*-strep mutant.

a The model for the generated mutant indicating the position of the insertion of the plasmid and the place of annealing of used oligonucleotides is shown**. b** The segregation of the AFS-*anacyp40*-strep mutant was analyzed with gene and plasmid specific oligonucleotides (Supplementary Table 7) as indicated in (a) on genomic DNA isolated from wild-type (WT) and from AFS-*anacyp40*-strep (MT). The base-pair number for the migration of the molecular main marker is indicated on the left.**c** The specific precipitation of *ana*Cyp40-strep was tested after solubilisation of wild-type cells and cells from AFS-*anacyp40*-strep. The cell lysate (CL), flow through (FL), last wash fraction (LW) and Elution 1 and 2 (E1 / E2) was subjected to SDS-PAGE followed by Western Blotting. Shown is the DB71 stain (top) and the detection with anti-strep antibodies. In the cell lysate and flow through a protein band cross-reactive with the used antibodies is detectable in wild type strain, which is no longer detectable in in the last wash fraction or elution. In turn, the protein *ana*Cyp40-strep is detectable in the lysate, the flow through and the elution fraction of AFS-*anacyp40*-strep cell lysate. The protein is specifically enriched as found by DB71 staining. The migration of the molecular weight standards is indicated on the left and the molecular weight is given in kDa.

SUPPLEMENTAL FIGURES

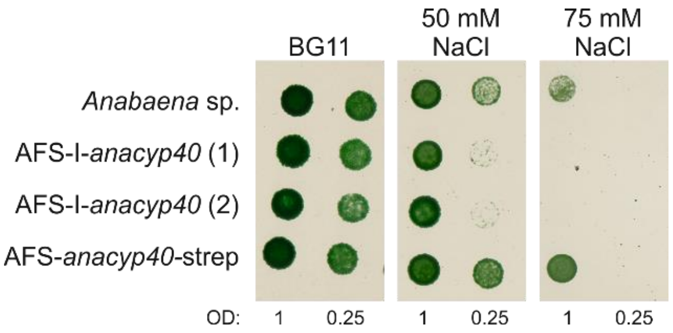

Supplementary Fig. 5: The wild-type and AFS-*anacyp40*-strep response to high NaCl.

The *Anabaena* sp. wild type, two independently generated AFS-I-a*naCyp40* strains (marked with (1) and (2)) as well as the AFS-I-*anacyp40-strep* mutant were spotted onto BG11 control plates and NaCl-containing BG11 plates (50 mM and 75 mM NaCl as indicated). The suspensions had an initial optical density (750 nm) of 1 or 0.25 (indicated at the bottom). The images were taken after 7 days of growth.

SUPPLEMENTAL FIGURES

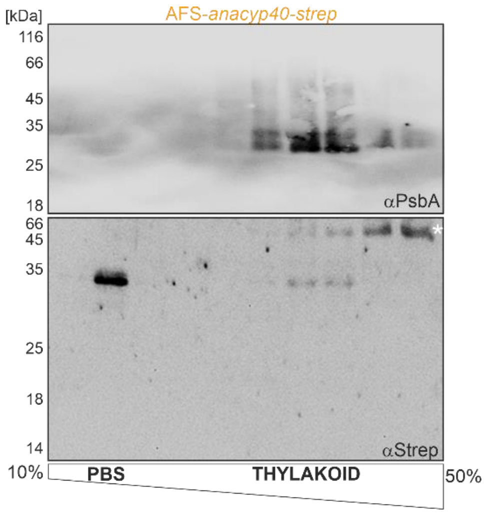

Supplementary Fig. 6: Full blot for Figure 4d.

The strain AFS-*anaCyp40*-strep was grown in BG11, cells harvested and solubilized and subjected on top of a 10-50% sucrose gradient. The fractions were subjected to SDS-PAGE followed by Western blotting and incubation with indicated antibodies. PBS and Thylakoid membrane fractions are indicated. The star indicates a cross reactivity of the antibody. The migration of the molecular weight standards is indicated on the left and the molecular weight is given in kDa.

SUPPLEMENTAL FIGURES

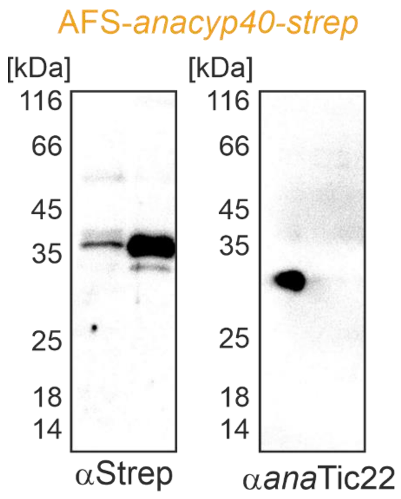

Supplementary Fig. 7: Full blot for Figure 4e.

Cell lysate (lane 1) and phycobilisomes (lane 2) were isolated from AFS-*anaCyp40*-strep and subjected to SDS-PAGE followed by Western blotting and incubation with indicated antibodies. The migration of the molecular weight standards is indicated on the left of each blot and the molecular weight is given in kDa.

SUPPLEMENTAL FIGURES

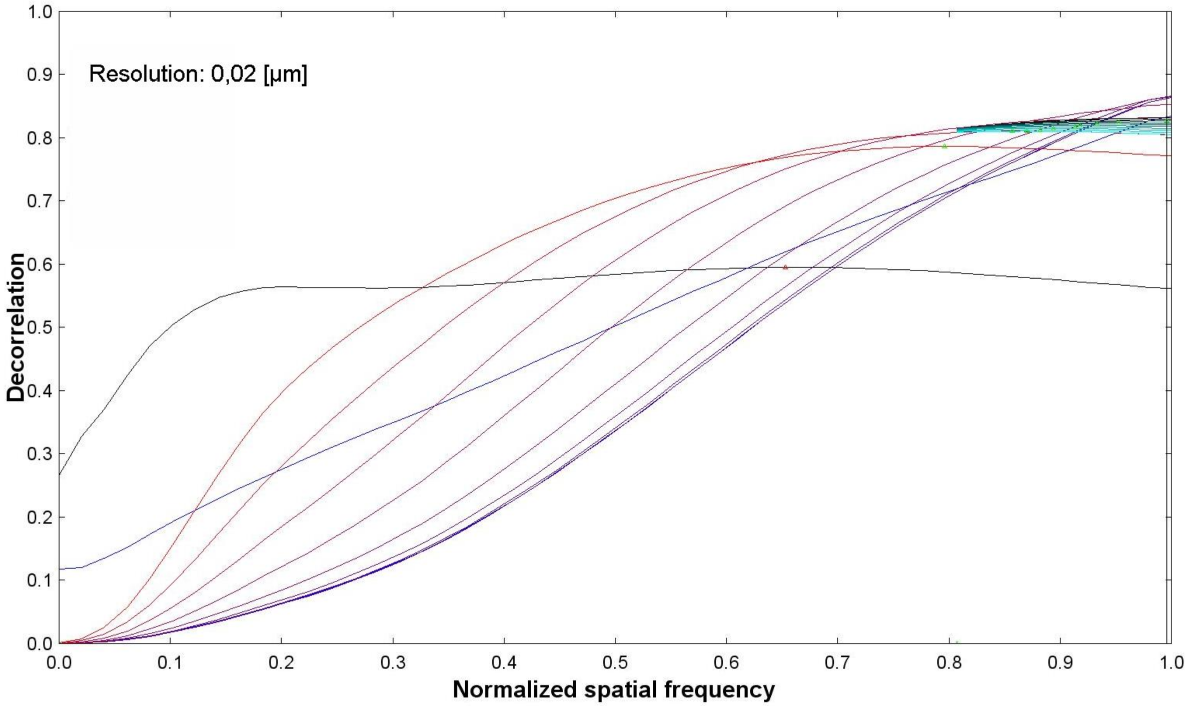

Supplementary Fig. 8: Estimated resolution of the super-resolution microscopy images. High-pass filtered decorrelation functions of *ana*Cyp40 visualized with DNA-PAINT (super-resolution microscopy). A Parameter-free image resolution estimation based on decorrelation analysis was performed.[5] The resolution of the *ana*Cyp40 channel was determined to be 20 nm, which exceeds the maximum theoretical resolution of regular confocal imaging by about a factor of 7.[6]

SUPPLEMENTAL FIGURES

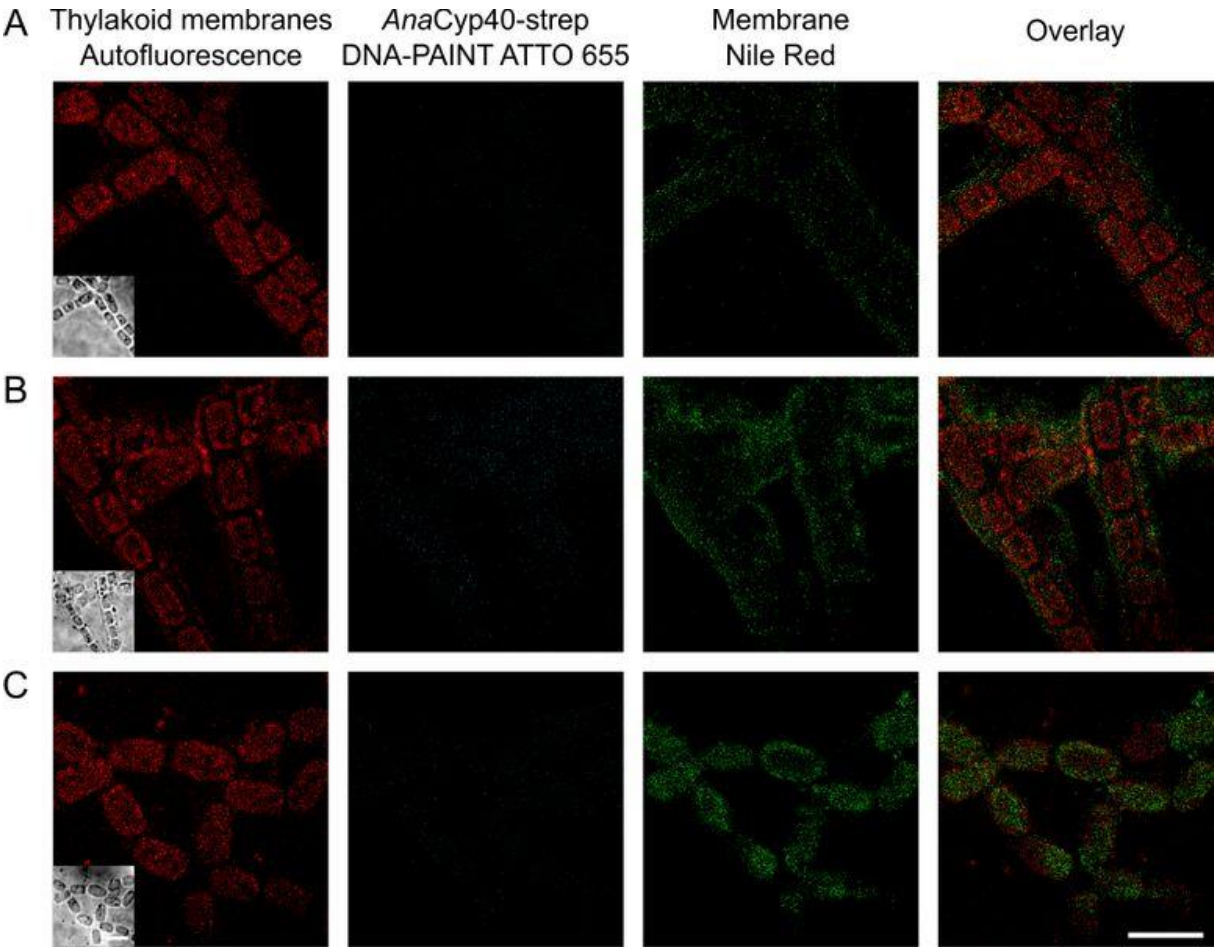

Supplementary Fig. 9: Super-resolution microscopy images of the wild type control. Control measurements to evaluate specific antibody labelling were performed as described in material and methods. The endogenous fluorophores (red), *ana*Cyp40-strep (cyan) and the membrane (green) were visualized in cyanobacteria only stained with the primary antibody (A), the secondary antibody (B), or in unstained cyanobacteria (C), using identical imager strad concentrations and imaging conditions to measurements shown in Figure 5. In all controls the endogenous fluorophores and membrane are clearly visible, but only few localizations were detected for anaCyp40 strep. Representative images of multiple recordings are shown. All images are adjusted to the same scale as indicated by the bar [5 µm] in the right corner.

SUPPLEMENTAL FIGURES

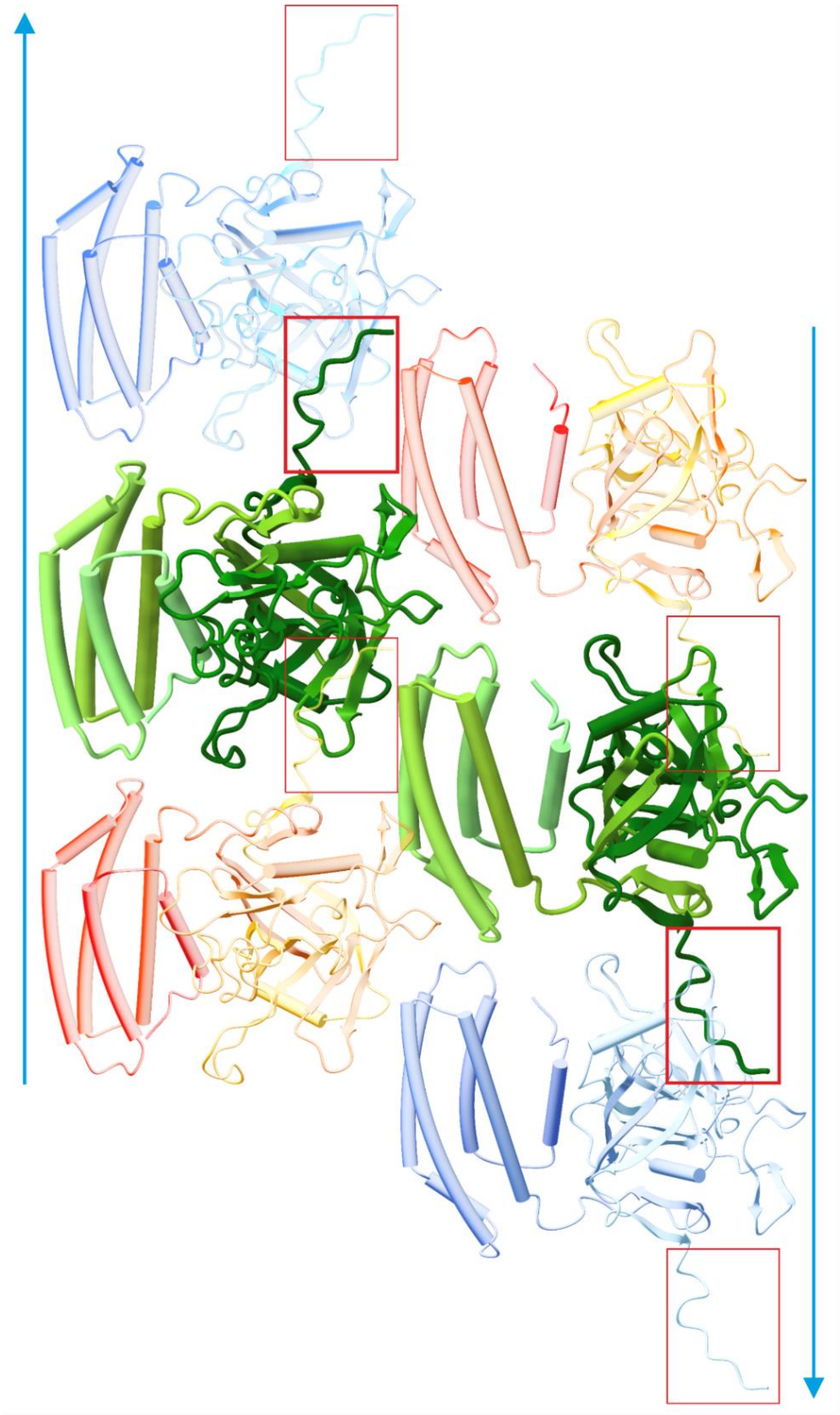

Supplementary Fig. 10: The interactions of monomers in the *ana*Cyp40 crystal.

A cutout of the molecular organization of *ana*Cyp40 monomers in the crystal. The monomers form layers in which the monomers form antiparallel rows, indicated by the two blue oppositely oriented arrows. The residual amino acids of the C-terminal cleavage site (red box) are involved in the crystal contacts between *ana*Cyp40 molecules and interacts with residues on the β-barrel surface of the next molecule.

SUPPLEMENTAL FIGURES

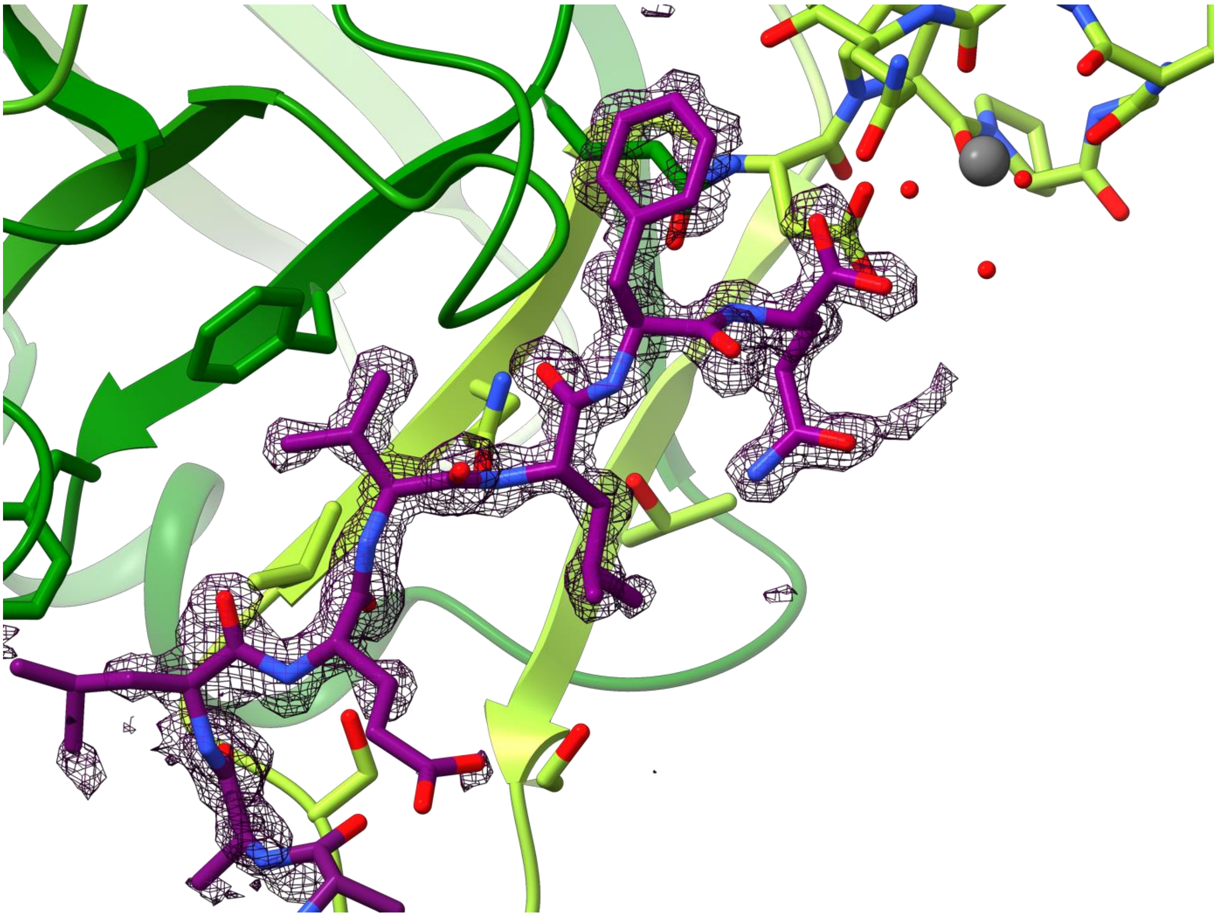

Supplementary Fig. 11: Crystal contacts between the C-terminus of one *ana*Cyp40 molecule with C-domain of a second molecule. The C-terminal amino acids “LEVLFQ” from the PreScission protease recognition site are shown in the Fo-Fc omit map at 3σ contour level.

SUPPLEMENTAL FIGURES

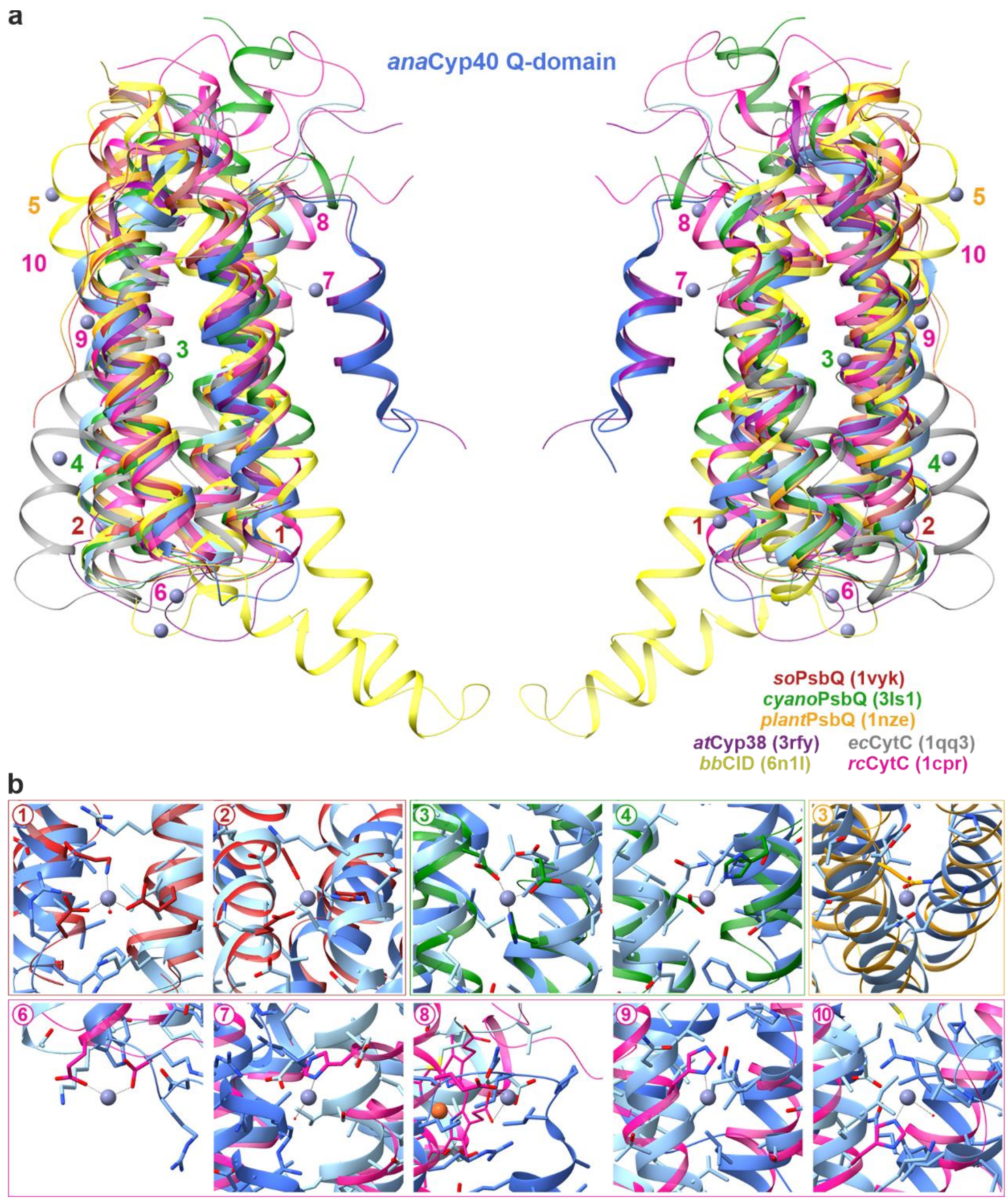

Supplementary Fig. 12: Comparison of *ana*Cyp40 Q-domain to structurally related proteins. **a** The N-terminal Q-domain of *ana*Cyp40 is superimposed to diverse helix bundles of structurally related proteins identified by DALI[7] and which have an RMSD < 2 Å. In addition to the cyanobacterial and plant PsbQs (1vyk[8]: red; 3ls1[9]: green; 1nze[10]: orange), also bacterial cytochrome C (1qq3[11]: grey, 1cpr[12]: magenta), the complement inhibitory domain of BBK32 (6n1l[13]: yellow), and the Q-domain of *at*Cyp38 (3rfy[14]: purple) are superimposed to *ana*Cyp40 Q-domain. Although most of these proteins have Zn^{2+} bound on their surface, neither the positions of the Zn^{2+} ion nor the coordinating residues are conserved. **b** Individual comparison of the sites where Zn^{2+} ions were bound and the corresponding site in anaCyp40. The location of the Zn^{2+} ions on the helix bundle is indicated by the numbers.

SUPPLEMENTAL FIGURES

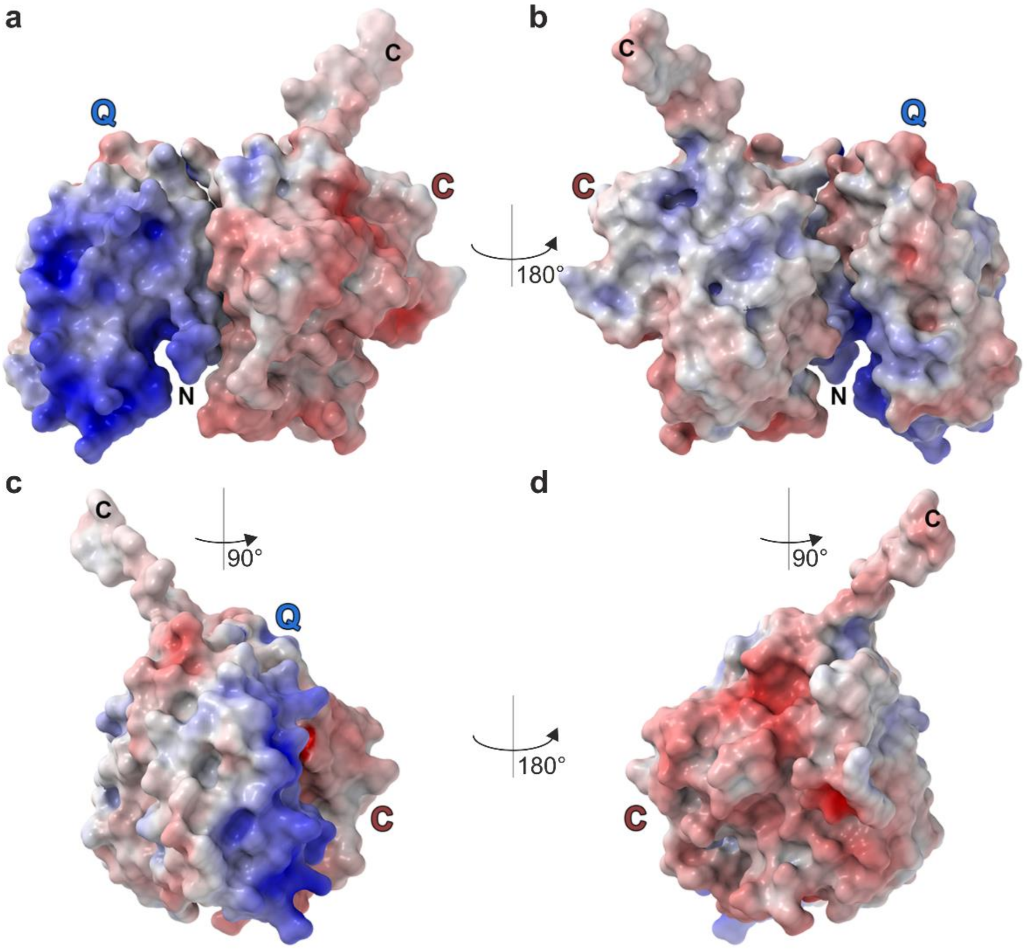

Supplementary Fig. 13: The electrostatic surface potential distribution of *ana*Cyp40. Electrostatic surface potential distribution of *ana*Cyp40 seen from different perspectives. **a** and **b** show the representation presented in Fig. 6a, while **c** and **d** are counter-clockwise and clockwise 90° vertical rotations of a, respectively. The position of the N- and C-terminus (small letters), as well as of the Q- and C-domain (large letters) are indicated. The Q-domain is mainly positively charged (shown in blue) while the C domain is slightly negatively charged (shown in red).

SUPPLEMENTARY TABLES

Supplementary Table 1: List of genes used for phylogenetic analysis. Given is the organism name, the protein ID and the gene accession number. The sequences are provided as Supplementary data 1.

	Organism	Protein ID	Gene
1.	*Nostoc sp.* (strain PCC 7120 / SAG 25.82 / UTEX 2576)	Q8YM80	alr5059
2.	*Gloeobacter violaceus* (strain PCC 7421)	Q7NG65	gll3308
3.	*Anabaena cylindrica* (strain ATCC 27899 / PCC 7122)	K9Z9J9	Anacy_0270
4.	*Nostoc punctiforme* (strain ATCC 29133 / PCC 73102)	B2J712	Npun_F0441
5.	*Nostoc sp.* (strain ATCC 29411 / PCC 7524)	K9QZX2	Nos7524_5280
6.	*Anabaena variabilis* (strain ATCC 29413 / PCC 7937)	Q3MAQ2	Ava_2316
7.	*Nostoc azollae* (strain 0708)	D7E1K7	Aazo_0811
8.	*Cyanobacterium stanieri* (strain ATCC 29140 / PCC 7202)	K9YIG0	Cyast_0282
9.	*Microcystis aeruginosa* (strain NIES-843)	B0JTU3	MAE_13090
10.	*Cyanothece sp.* (strain ATCC 51142)	B1WP74	cce_0505
11.	*Arthrospira platensis* (strain NIES-39 / IAM M-135)	D4ZPR7	NIES39_D07450
12.	*Trichodesmium erythraeum* (strain IMS101)	Q116D7	Tery_1312
13.	*Lyngbya sp.* (strain PCC 8106)	A0YPV7	L8106_21422
14.	*Stanieria cyanosphaera* (strain ATCC 29371 / PCC 7437)	K9Y016	Sta7437_4152
15.	*Acaryochloris marina* (strain MBIC 11017)	B0CD62	AM1_0603
16.	*Chamaesiphon minutus* (strain ATCC 27169 / PCC 6605)	K9UJP7	Cha6605_3707
17.	*Synechocystis sp.* (strain PCC 6803 / Kazusa)	PPI3_SYNY3	sll0408
18.	*Prochlorococcus marinus* (strain AS9601)	A2BNF1	A9601_00241
19.	*Cyanobium gracile* (strain ATCC 27147 / PCC 6307)	K9P908	Cyagr_2330
20.	*Synechococcus sp.* (strain ATCC 27144 / PCC 6301 / SAUG 1402/1)	A0A0H3K3E3	ppiB
21.	*Thermosynechococcus elongatus* (strain BP-1)	Q8DKN8	tlr0821
22.	*Vitrella brassicaformis* (strain CCMP3155)	A0A0G4EJ74	Vbra_20474
23.	*Emiliania huxleyi*	R1F9L9	EMIHUDRAFT_440955
24.	*Cyanidioschyzon merolae*	M1USH1	CYME_CMK307C
25.	*Galdieria sulphuraria*	M2XYR0	Gasu_36910
26.	*Chondrus crispus*	R7QPR7	CHC_T00000384001
27.	*Phaeodactylum tricornutum* (strain CCAP 1055/1)	B7FU48	PHATRDRAFT_11022
28.	*Ectocarpus siliculosus*	D8LET7	CYN
29.	*Chlamydomonas reinhardtii*	A8IRU6	CYN38
30.	*Volvox carteri*	D8UIB6	VOLCADRAFT_108119
31.	*Chlorella variabilis*	E1Z2A2	CHLNCDRAFT_33486
32.	*Ostreococcus lucimarinus* (strain CCE9901)	A4RWY9	OSTLU_38395
33.	*Micromonas commoda* (strain RCC299 / NOUM17 / CCMP2709)	C1FEJ6	TLP40
34.	*Klebsormidium flaccidum*	KFL_000140480	KFL_000140480
35.	*Marchantia polymorpha*	Mapoly0001s0360	
36.	*Oryza sativa subsp. indica*	B8BAB3	OsI_29072
37.	*Brachypodium distachyon*	I1I6Q4	100824311
38.	*Hordeum vulgare subsp. vulgare*	A0A287WPI4	n/a
39.	*Aegilops tauschii*	M8CD05	F775_28503
40.	*Triticum aestivum*	A0A1D6CYY9	-
41.	*Eragrostis tef*	maker-scaffold7841-a	-
42.	*Sorghum bicolor*	A0A1Z5RAB0	SORBI_3007G116950
43.	*Zea mays*	B6U5I1	100285487
44.	*Setaria italica*	K3YHT3	101781132
45.	*Musa acuminata*	GSMUA_Achr1G13990_00	-
46.	*Nicotiana attenuata*	A0A1J6IL97	TLP40
47.	*Solanum lycopersicum*	K4BBJ8	XP_004232290.1
48.	*Cucumis sativus*	A0A0A0LHY2	Csa_3G836450
49.	*Lotus japonicus*	Lj2g3v1014320	-
50.	*Manihot esculenta*	A0A2C9UIA1	MANES_15G183100
51.	*Populus trichocarpa*	U5GVV7	-
52.	*Prunus persica*	M5WTM9	PRUPE_3G283500
53.	*Arabidopsis thaliana*	CYP40_ARATH	CYP40
54.	*Theobroma cacao*	A0A061ED69	TCM_016868
55.	*Corchorus capsularis*	A0A1R3IDS8	CCACVL1_12798
56.	*Vitis vinifera*	D7TWR2	VIT_14s0066g01410

57.	*Amborella trichopoda*	W1PHN6	AMTR_s00058p00167600
58.	*Physcomitrella patens subsp. patens*	A9T157	PHYPA_030052
59.	*Chenopodium quinoa*	-	XP_021727682
60.	*Glycine max*	I1N5T2	GLYMA_19G009700
61.	*Brassica napus*	A0A078IGH2	BnaC05g48850D
62.	*Gossypium hirsutum*	A0A1U8HQH8	LOC107888646
63.	*Selaginella moellendorffii*	D8R8R1	SELMODRAFT_408578
64.	*Deinococcus radiodurans* R1	Q9RRF0	DR_2542
65.	*Streptococcus pneumoniae* R6	Q8DQG5	ppiA
66.	*Homo sapiens*	Q9Y3C6	PPIL1
67.	*Mus musculus*	Q9D0W5	Ppil1
68.	*Escherichia coli str.* K-12 substr. MG1655	P0AFL3	ppiA
69.	*Caenorhabditis elegans*	Q9U1Q3	cyn-15
70.	*Schizosaccharomyces pombe*	Q09928	cyp8
71.	*Saccharomyces cerevisiae* S288C	P23285	CPR2
72.	*Drosophila melanogaster*	Q9W0Q2	Cypl

SUPPLEMENTARY TABLES

Supplementary Table 2: Overview on transmembrane domain prediction. Transmembrane domain and signal sequence prediction was performed with TMHMM[15], TMpred[16], HMMTOP[17], Phobius[18], SPLIT[19] and PRED-TMR[20].

program	Start AA	End AA	Score
TMHMM	13	30	0.91
TMpred	12	30	2170
HMMTOP	13	30	17.0082
Phobius	13	24	0.87285
SPLIT	10	26	7.87
PRED-TMR	13	31	Not defined
**CONSENSE**	**13**	**30**	

SUPPLEMENTARY TABLES

Supplementary Table 3: Kinetic parameters of cyclophilins from different species

Species	Enzymatic activity	References
Anabaena PCC7120 (*ana*Cyp40)	0.07 s^{-1}	(This study)
Piriformospora indica (PiCyPA)	0.10 s^{-1}	(Trivedi et al. 2013)[21]
Arabidopsis thaliana (AtCyp19-3)	0.036 s^{-1}	(Kaur et al. 2015)[22]
Oryza sativa (TaCypA-1)	0.039 s^{-1}	(Sekhon et al. 2013)[23]
Zea mays (PPI)	0.08 s^{-1}	(Sheldon and Venis, 1996)[24]
Spinacia oleracea (TLP40)	0.04 s^{-1}	(Fulgosi et al. 1998)[25]

SUPPLEMENTARY TABLES

Supplementary Table 4: List of *Anabaena* strains used in this study

***Anabaena* strain**	**Resistance**	**Relevant properties**	**Source**
PCC 7120		Wild type	C. P. Wolk
AFS-*ana*cyp40-strep	Sp^r Sm^r	C-terminal Strep tag fusion	This study
AFS-I-*anacyp40*	Sp^r Sm^r	Gene interruption	This study

[a]AFS, Anabaena Frankfurt Schleiff; Sp, spectinomycin; Sm, streptomycin.

SUPPLEMENTARY TABLES

Supplementary Table 5: Proteins of the PBS. Given are the subcomplex annotation, the gene ID and the annotated name, the number of amino acids, the percentage of prolines in the sequence and the RPKM value extracted from Flaherty et al.[26].

Complex category	Gene ID	Name	aa	% proline	RPKM
APC	**Alr0020**	**ApcE**	**1132**	**6.0**	**96**
	**Alr0021**	**ApcA1**	**161**	**3.1**	**275**
	**Alr0022**	**ApcB**	**162**	**1.9**	**313**
	**Asr0023**	**ApcC**	**68**	**2.9**	**153**
	All0450	ApcA2	161	3.1	7
	All2327	ApcF	169	3.0	26
	All3653	ApcD	161	4.3	67
CPC	Alr0528	CpcB	173	2.3	337
	Alr0529	CpcA	163	3.7	879
	**Alr0530**	**CpcC**	**266**	**3.5**	**956**
	**Asr0531**	**CpcD**	**80**	**1.2**	**923**
	Alr0532	CpcE	276	5.1	168
	Alr0533	CpcF	200	5.0	29
	Alr0534	CpcG1	279	5.0	25
	**Alr0535**	**CpcG2**	**247**	**6.1**	**43**
	Alr0536	CpcG3	237	4.2	58
	**Alr0537**	**CpcG4**	**253**	**3.2**	**42**
PEC	Alr0523	PecB	172	1.2	1230
	Alr0524	PecA	162	3.7	152
	**Alr0525**	**PecC**	**278**	**2.5**	**166**
	Alr0526	PecE	253	4.3	30
	Alr0527	PecF	173	2.3	3

SUPPLEMENTARY TABLES

Supplementary Table 6: Photosynthetic parameter of wild-type and AFS-I-*anacyp40*.

	WT		AFS-I-*anacyp40*	
	40 µE	120 µE	40 µE	120 µE
Pm (µmol min^{-1} mg_{CHL}^{-1})*	4.5±0.3	6.0±0.3	5.1±0.3	3.3±0.2
Ec (µE m^{-2} s^{-1})**	110±10	150±20	130±20	190±30
F_{PSI}/F_{PSII} (590 nm)	0.17	0.17	0.16	0.18
F_{PSI}/F_{PSII} (440 nm)	2.3	0.9	2.0	0.9

* Value for 1000 µE m^{-2} s^{-1}, ** estimated according to Ec = - [100 µE m^{-2} s^{-1}]/ln (1-[OER] / Pm); [O] = Pm * (1 – exp (-[L]/Ec)) – Ek; Pm … maximal rate of net oxygen production at light saturation; Ec … compensation irradiance, at which oxygen production was balanced by oxygen consumption; Ek … the irradiance at the onset of photosynthesis saturation

SUPPLEMENTARY TABLES

Supplementary Table 7: Data collection and refinement statistics	
	*ana*Cyp40$_{\Delta TM}$
Data collection	
Space group	*P* 2$_1$ (4)
Cell dimensions	a = 41.2 Å, b = 103.2 Å, c = 44.2 Å<br>β = 114.7°
Matthews coeff. (Å^{3}Da^{-1})	2.3
Solvent content (%)	46.5
No. Molecules per AU	1
Resolution (Å)	52 – 1.14 (1.14 – 1.18)
Wavelength (Å)	λ = 1.0
X-ray source	ESRF ID29
No. observed reflections	1706153 (64337)
No. unique reflections	115733 (5453)
Completeness (%)	95 (91.3)
R_{meas} (%)	0.22 (0.754)
R_{pim} (%)	0.058 (0.788)
$I/I\sigma$	7.5 (1.6)
Refinement	
Resolution (Å)	50 - 1.14
No. unique reflections	115662
No. Reflections in test set	5617
R_{work}/R_{free} (%)	17.68/19.47
Wilson B-Factor (Å^2)	48.81
No. atoms in AU	3445
Protein	2664
Ligands	31
Water	579
r.m.s. deviations:	
Bond lengths (Å)	0.014
Bond angels (°)	1.371

SUPPLEMENTARY TABLES

Supplementary Table 8: Oligonucleotides used in this study.

Primer Name	Sequence
***Anabaena* sp. mutant generation**	
AFS-I-*anacyp40*-F1	GGATCCAATAAATAATAAGCCAGTACG
AFS-I-*anacyp40*-R1	GGATCCGTAAATTCTAACCCATTATAG
AFS-*anacyp40-strep*-F1	ATCGATGCTGCATTGAGCTATTCC
AFS-*anacyp40-strep*-R1	TTACTTTTCGAACTGCGGGTGGCTCCAGGCTGATTGGGGTTGTACTAAA
***Anabaena* sp. mutant confirmation by colony PCR**	
AFS-I-*anacyp40*-F2	CGGTAGAGCTTTATTGCGGTA
AFS-I-*anacyp40*-R2	GTTCTTTACCTGGTGGATCTCC
AFS-*anacyp40-Strep*-F2	GATTGATGGGTTACGCCTAGTC
AFS-*anacyp40-Strep*-R2	CTTTTCGAACTGCGGGTGGCTCCA
**Cloning for heterologous expression**	
*ana*cyp40$_{\Delta TM(1-35)}$-F3	ATATATGCTCTTCTAGTGCCGCCTTGCCATCTGGAAATGCGATT
*ana*cyp40$_{\Delta TM(1-35)}$-R3	TATATAGCTCTTCATGCGGCTGATTGGGGTTGTACTAAATTGTC
**q-RT PCR**	
*ana*cyp40-F4	TTGCGGTATGCACTCCCAAT
*ana*cyp40-R4	GCGGGATGCTTTACTCAGGT
RT-as5059	GGACTCCTTCCAGTAACGCC
RT-5059	GCTGTAACCATCCACCACAAC
5059-fw	CCTTGCCATCTGGAAATGCG
5059-rv	GCTTGCAGTTCGCGTACTGG
rnpB-rv	GGGTTTACCGAGCCAGTACC
rnpB-fw	AGGGAGAGAGTAGGCGTTGG
16SrRNA-R	CACACTGGGACTGAGACAC
16SrRNA-R	CTGCTGGCACGGAGTTAG
**Sequencing primers**	
T7	TAATACGACTCACTATAGGG
SP6	ATTTAGGTGACACTATAG

SUPPLEMENTARY TABLES

Supplementary Table 9: Plasmids used in this study.

Plasmid	Marker[a]	Properties	Source or reference
pGEM-T	Ap^r	TA cloning vector	Promega
pGEM-T-Easy	Ap^r	TA cloning vector	Promega
pCSV3	Sp^r Sm^r	pRL500 with substituted Ap^r gene	Olmedo-Verd et al.[27]
pRL623	Cm^r	Mobilization helper and methylases for AvaI-AvaII and AvaIII sites	Elhai et al.[28]
pRL443	Ap^r	Shuttle vector derived from RP4 suitable for mobilizing plasmids to *Anabaena* sp. PCC 7120	Elhai et al.[28]
pINITIAL	Cm^r	Initial sequencing vector for FX cloning	Geertsma and Dutzler [29]
pF7XC3H	Cm^rKan^r	Expression vector for FX subcloning	Geertsma and Dutzler [29]
pGEMT-I-*ana*cyp40	Ap^r	pGEMT with frag. (bp 168-665) of alr5059	This study
pGEMT-Easy-*ana*cyp40-strep	Ap^r	pGEMT Easy with full length alr5059 and a C-terminal *Strep*-tag	This study
pCSV3-I-*ana*cyp40	Sp^r Sm^r	pCSV3 with frag. (bp 168-665) of alr5059	This study
pCSV3-*ana*cyp40-strep	Sp^r Sm^r	pCSV3 with full length alr5059 and a C-terminal *Strep*-tag	This study
pINIT_cat-*ana*cyp40$_{\Delta TM(1\text{-}35)}$	Cm^r	pINIT_cat with alr5059 frag. (aa 36-368)	This study
F7XC3H-*ana*cyp40$_{\Delta TM(1\text{-}35)}$-HRV 3C- His_{10}	Cm^rKan^r	F7XC3H with alr5059 frag. (aa 36-368)	This study

REFERENCES IN SUPPLEMENTARY INFORMATION ONLY

1. Stevanovic M, Lehmann C, Schleiff E. The response of the TonB-dependent transport network in Anabaena sp. PCC 7120 to cell density and metal availability. *Biometals.* 26, 549-560 (2013).

2. Ho, E.C., Donaldson, M.E. and Saville, B.J., 2010. Detection of antisense RNA transcripts by strand-specific RT-PCR. In *RT-PCR Protocols* (pp. 125-138). Humana Press, Totowa, NJ.

3. Brenes-Álvarez, M. et al. Elements of the heterocyst-specific transcriptome unravelled by co-expression analysis in *Nostoc sp.* PCC 7120. *Environ. Microbiol.* **21,** 2544-2558 (2019).

4. Haddad, F., Qin, A.X., Giger, J.M. *et al.* Potential pitfalls in the accuracy of analysis of natural sense-antisense RNA pairs by reverse transcription-PCR. *BMC Biotechnol* **7,** 21 (2007).

5. Descloux, A., Grußmayer, K.S. & Radenovic, A. Parameter-free image resolution estimation based on decorrelation analysis. *Nat Methods* **16,** 918–924 (2019).

6. Paddock, S.W. Confocal laser scanning microscopy. *Biotechniques* **27**, 992–1004 (1999).

7. Holm, L. DALI and the persistence of protein shape. *Protein Sci.* **29,** 128-140 (2020).

8. Balsera, M., Arellano, J.B., Revuelta, J.L., de las Rivas, J., Hermoso, J.A. The 1.49 A resolution crystal structure of PsbQ from photosystem II of *Spinacia oleracea* reveals a PPII structure in the N-terminal region. *J. Mol. Biol*. **350,** 1051-1060 (2005).

9. Jackson, S.A., Fagerlund, R.D., Wilbanks, S.M., Eaton-Rye, J.J. Crystal structure of PsbQ from Synechocystis sp. PCC 6803 at 1.8 A: implications for binding and function in cyanobacterial photosystem II. *Biochem.* **49,** 2765-2767 (2010).

10. Calderone, V., Trabucco, M., Vujicić, A., *et al.* Crystal structure of the PsbQ protein of photosystem II from higher plants. *EMBO Rep.* **4,** 900-905 (2003).

11 Arnesano, F., Banci, L., Bertini, I., Ciofi-Baffoni, S., Woodyear, T.L., Johnson, C.M. & Barker, P.D. Structural consequences of b- to c-type heme conversion in oxidized Escherichia coli cytochrome b562. *Biochem.* **39,** 1499-1514 (2000).

12 Tahirov, T.H., Misaki, S., Meyer, T.E., Cusanovich, M.A., Higuchi, Y. & Yasuoka, N. Structure of cytochrome c' from Rhodobacter capsulatus strain St Louis: an unusual molecular association induced by bridging Zn ions. *Acta. Crystallogr. D* **53,** 658-664 (1997).

13 Xie, J., Zhi, H., Garrigues, R.J., Keightley, A., Garcia, B.L. & Skare, J.T. Structural determination of the complement inhibitory domain of Borrelia burgdorferi BBK32 provides insight into classical pathway complement evasion by Lyme disease spirochetes. *PLoS Pathog.* **15,** e1007659 (2019).

14. Vasudevan, D., Fu, A., Luan, S. & Swaminathan, K. Crystal structure of Arabidopsis cyclophilin38 reveals a previously uncharacterized immunophilin fold and a possible autoinhibitory mechanism. *Plant Cell,* **24,** 2666-2674 (2012).

15. Krogh, A., Larsson, B., von Heijne, G. & Sonnhammer, E. L. Predicting transmembrane protein topology with a hidden Markov model: application to complete genomes. *J. Mol. Biol.* **305,** 567-580 (2001).

16. Hofmann, K. & Stoffel W. TMbase - A database of membrane spanning proteins segments. *Biol. Chem. Hoppe-Seyler* **374,**166 (1993).

17. Tusnady, G. E. & Simon, I. The HMMTOP transmembrane topology prediction server. *Bioinformatics,* **17,** 849-850 (2001).

18. Kall, L., Krogh, A. & Sonnhammer, E.L. A combined transmembrane topology and signal peptide prediction method. *J. Mol. Biol.* **338,** 1027-1036 (2004).

19. Juretic, D., Zoranic, L. & Zucic, D. Basic charge clusters and predictions of membrane protein topology. *J. Chem. Inf. Comput. Sci.* **42,** 620-632 (2002).

20. Pasquier, C., Promponas, V.J., Palaios, G.A., Hamodrakas, J.S. & Hamodrakas, S.J. A novel method for predicting transmembrane segments in proteins based on a statistical analysis of the SwissProt database: the PRED-TMR algorithm. *Protein Eng*. **12,** 381-385 (1999).

21. Trivedi, D.K., Ansari, M.W., Dutta, T., Singh, P. and Tuteja, N., 2013. Molecular characterization of cyclophilin A-like protein from Piriformospora indica for its potential role to abiotic stress tolerance in E. coli. *BMC research notes*, *6*(1), pp.1-9.

22. Kaur, G., Singh, S., Singh, H., Chawla, M., Dutta, T., Kaur, H., Bender, K., Snedden, W.A., Kapoor, S., Pareek, A. and Singh, P., 2015. Characterization of peptidyl-prolyl cis-trans isomerase-and calmodulin-binding activity of a cytosolic Arabidopsis thaliana cyclophilin AtCyp19-3. *PloS one*, *10*(8), p.e0136692.

23. Sekhon, S.S., Kaur, H., Dutta, T., Singh, K., Kumari, S., Kang, S., Park, S.G., Park, B.C., Jeong, D.G., Pareek, A. and Woo, E.J., 2013. Structural and biochemical characterization of the cytosolic wheat cyclophilin TaCypA-1. *Acta Crystallographica Section D: Biological Crystallography*, *69*(4), pp.555-563.

24. Sheldon, P.S. and Venis, M.A., 1996. Purification and characterization of cytosolic and microsomal cyclophilins from maize (Zea mays). *Biochemical Journal*, *315*(3), pp.965-970.

25. Fulgosi, H., Vener, A. V., Altschmied, L., Herrmann, R. G. & Andersson, B. A novel multi-functional chloroplast protein: identification of a 40 kDa immunophilin-like protein located in the thylakoid lumen. *EMBO J*, **17,** 1577-1587 (1998).

26. Flaherty, B.L., Van Nieuwerburgh, F., Head, S.R. & Golden, J.W. Directional RNA deep sequencing sheds new light on the transcriptional response of Anabaena sp. strain PCC 7120 to combined-nitrogen deprivation. *BMC Genomics.* **12,** 332 (2011).

27. Olmedo-Verd, E., Muro-Pastor, A.M., Flores, E. & Herrero, A. Localized induction of the ntcA regulatory gene in developing heterocysts of Anabaena sp. strain PCC 7120. *J. Bacteriol.* **188,** 6694-6699 (2006).

28. Elhai, J., Vepritskiy, A., Muro-Pastor, A.M., Flores, E. & Wolk, C.P. Reduction of conjugal transfer efficiency by three restriction activities of Anabaena sp. strain PCC 7120. *J. Bacteriol.* **179,** 1998-2005 (1997).

29. Geertsma, E.R. & Dutzler, R. A Versatile and Efficient High-Throughput Cloning Tool for Structural Biology." *Biochem.* **50,** 3272-3278 (2011).
