# Supplementary material for: Cyclophilin anaCyp40 regulates photosystem assembly and phycobilisome association in a cyanobacterium
Source: Nat Commun. 2022 Mar 30;13:1690. doi: 10.1038/s41467-022-29211-w (PMC8967839; doi:10.1038/s41467-022-29211-w)
Supplement: Supplementary file 2 — Reporting Summary [file 41467_2022_29211_MOESM2_ESM.pdf]

nature portfolio

Corresponding author(s): Enrico Schleiff

Last updated by author(s): 20.02.2022

Reporting Summary

Nature Portfolio wishes to improve the reproducibility of the work that we publish. This form provides structure for consistency and transparency in reporting. For further information on Nature Portfolio policies, see our Editorial Policies and the Editorial Policy Checklist.

Statistics

For all statistical analyses, confirm that the following items are present in the figure legend, table legend, main text, or Methods section.

n/a	Confirmed	
☐	☒	The exact sample size (*n*) for each experimental group/condition, given as a discrete number and unit of measurement
☐	☒	A statement on whether measurements were taken from distinct samples or whether the same sample was measured repeatedly
☐	☒	The statistical test(s) used AND whether they are one- or two-sided<br>*Only common tests should be described solely by name; describe more complex techniques in the Methods section.*
☐	☒	A description of all covariates tested
☐	☒	A description of any assumptions or corrections, such as tests of normality and adjustment for multiple comparisons
☐	☒	A full description of the statistical parameters including central tendency (e.g. means) or other basic estimates (e.g. regression coefficient) AND variation (e.g. standard deviation) or associated estimates of uncertainty (e.g. confidence intervals)
☐	☒	For null hypothesis testing, the test statistic (e.g. *F*, *t*, *r*) with confidence intervals, effect sizes, degrees of freedom and *P* value noted<br>*Give P values as exact values whenever suitable.*
☒	☐	For Bayesian analysis, information on the choice of priors and Markov chain Monte Carlo settings
☒	☐	For hierarchical and complex designs, identification of the appropriate level for tests and full reporting of outcomes
☐	☒	Estimates of effect sizes (e.g. Cohen's *d*, Pearson's *r*), indicating how they were calculated

Our web collection on statistics for biologists contains articles on many of the points above.

Software and code

Policy information about availability of computer code

Data collection	MEGA (version 6), rapidSTORM 3.0, Unicorn 4.1, TMHMM 1.0, TMpred, HMMTOP (transmembrane topology prediction server), Phobius, SPLIT 4.0 SERVER, PRED-TMR version 1.0, CD serach (a NCBI interface) , SignalP V 5.0
Data analysis	Bio-Rad CFX Manager (version 3.1), FLUORCAM 7 (version 1.2.5.16), Sigma Plot (version 11.0), SPSS, ImageJ 2.0.0-rc-3, Fiji (an image processing package of ImageJ2), µManager 1.4.14, Origin 2019, LAMA software v16.10, XDS program package, PHASER, CCP4 program suite, COOT, phenix.refine, PDB2PQR, APBS, UCSF ChimeraX

For manuscripts utilizing custom algorithms or software that are central to the research but not yet described in published literature, software must be made available to editors and reviewers. We strongly encourage code deposition in a community repository (e.g. GitHub). See the Nature Portfolio guidelines for submitting code & software for further information.

Data

Policy information about availability of data

All manuscripts must include a data availability statement. This statement should provide the following information, where applicable:

- Accession codes, unique identifiers, or web links for publicly available datasets
- A description of any restrictions on data availability
- For clinical datasets or third party data, please ensure that the statement adheres to our policy

Plasmids and cyanobacterial strains are available upon request. The atomic coordinates for anaCyp40ΔTM have been deposited in the Protein Data Bank with the accession code 7A73. The source data underlying Figs. 1, 2, 3, 5 are provided as a Source Data file. Other data that support the findings of this study are available upon reasonable request to the corresponding author. Source data are provided with this paper.

Field-specific reporting

Please select the one below that is the best fit for your research. If you are not sure, read the appropriate sections before making your selection.

- [x] Life sciences
- [ ] Behavioural & social sciences
- [ ] Ecological, evolutionary & environmental sciences

For a reference copy of the document with all sections, see nature.com/documents/nr-reporting-summary-flat.pdf

Life sciences study design

All studies must disclose on these points even when the disclosure is negative.

Sample size	No statistical size determination was performed. For biological experiments sufficient independent experiments were performed (at least 3) which contained the appropriate amount of replicates to ensure sufficient power to achieve significant statistical differences.
Data exclusions	No data were excluded from the analysis.
Replication	At-least three replicates were taken to verify the reproducibility of the experimental findings. All attempts at replication were successful.
Randomization	The experiments were done under a completely randomized design
Blinding	Thre investigators were not blinded during data collection, but the experiments were all done in an unbiased manner and validity was ensured by independent repetitions.

Reporting for specific materials, systems and methods

We require information from authors about some types of materials, experimental systems and methods used in many studies. Here, indicate whether each material, system or method listed is relevant to your study. If you are not sure if a list item applies to your research, read the appropriate section before selecting a response.

Materials & experimental systems

n/a	Involved in the study	
☐	☒	Antibodies
☒	☐	Eukaryotic cell lines
☒	☐	Palaeontology and archaeology
☒	☐	Animals and other organisms
☒	☐	Human research participants
☒	☐	Clinical data
☒	☐	Dual use research of concern

Methods

n/a	Involved in the study	
☒	☐	ChIP-seq
☒	☐	Flow cytometry
☒	☐	MRI-based neuroimaging

Antibodies

Antibodies used	Strep-Tactin® HRP conjugate (IBA Life Sciences IBA #2-1502-001 1:5000), rabbit anti-strep primary antibody (US biological Biomol # S7973-93T 1:100), anti-PsbA (#AS05084 1:10000), rabbit anti-anaTic22 (home-made 1:5000). AffiniPure Goat Anti-Mouse IgG (H+L) from Jackson Immuno Research (AB_2338447) was conjugated with a DNA strand (P1) and diluted 1:200 for cell staining.
Validation	The commercially available primary antibodies in this study have been chosen based on recommendations on the manufacturer's website, which includes citations and applications (https://www.iba-lifesciences.com/Strep-Tactin-conjugate/2-1502-001, https://www.biomol.com/de/search?sSearch=S7973-93T, https://www.agrisera.com/en/artiklar/psba-d1-protein-of-psii-c-terminal-100-l.html, https://www.jacksonimmuno.com/catalog/products/115-005-003). The homemade antibodies have been reported previously (DOI: 10.1074/jbc.M112.341644, DOI: 10.1111/mmi.14222)
