# Supplementary material for: Cyclophilin anaCyp40 regulates photosystem assembly and phycobilisome association in a cyanobacterium
Source: Nat Commun. 2022 Mar 30;13:1690. doi: 10.1038/s41467-022-29211-w (PMC8967839; doi:10.1038/s41467-022-29211-w)
Supplement: Supplementary file 3 — Description of Additional Supplementary Files [file 41467_2022_29211_MOESM3_ESM.pdf]

Title: Supplementary data 1
Description: Sequences used for the phylogenetic analysis.
Provided are all sequences used for the phylogenetic analysis shown in Figure 1 in FASTA format.

Title: Supplementary data 2
Description: Additional information to table 1.
Proteomic discovery: Given are the functional assignment to a complex, the gene name, the accession number, the Uniprot number, the extracted description and the p-value calculated as described in the manuscript. For the three experiments the peptide count and the LFQ value for each of the experimental replicas is provided. Note, for experiment 1 in total one experimental replica for the AFS-ana*cyp38*-strep strain and twelve controls were performed and used for the calculation of the LFQ value. The maximal LFQ value and the maximal peptide discovery observed is presented (control *). For experiment 2 in total four different control experiment were performed with three experimental replicas each were performed and used for LFQ calculation. Here, only the direct control (wild-type strain vs- AFS-ana*cyp38*-strep) is shown. Note, only identified proteins with $p<0.05$ are presented.
