# Supplementary material for: Cyclophilin anaCyp40 regulates photosystem assembly and phycobilisome association in a cyanobacterium
Source: Nat Commun. 2022 Mar 30;13:1690. doi: 10.1038/s41467-022-29211-w (PMC8967839; doi:10.1038/s41467-022-29211-w)
Supplement: Supplementary file 4 — SUPPLEMENTARY DATA 1 [file 41467_2022_29211_MOESM4_ESM.zip › SUPPLEMENTARY DATA 1.docx]

**SUPPLEMENTARY DATA**

**Supplementary Data 1: Sequences used for phylogenetic calculation.**

>NOSS105007 | Q8YM80 | self | [Nostoc sp. (strain PCC 7120 / SAG 25.82 / UTEX 2576)]

MLNLLKSWLKNSLMAILLVTIFLGINTAGWTPSSNAALPSGNAITDGRALLRYALPINNKPVRELQASLEDISAQLRANR

RWGAVSKDLSKASRILDKPSQILTSVPEERQTQAETWINELKTGVVKVQELAQSKDKEQVLLERAKLLNLVSLIEESMVK

EFPFEVPEEYNNLPQLKGRATIAIKTNKGDLTVVVDGYSAPVTAGNFVDLVKRGFYNGLEFTRSEESYVLQTGDPPGKEQ

GFIDPKTGKYRAIPLEILAEGDKKPTYGITLEDAGRYLDMPVLPFSSFGALAMARPETEVDGASSQVFFFLFEPELTPAG

RNLLDGRYSVFGYLIEGRDILDTLKAGDKIESATVVQGLDNLVQPQSA

>GLOVI03287 | Q7NG65 | 1:1 ortholog | [Gloeobacter violaceus (strain PCC 7421)]

MSAVEQKINELEQLTQTAEFQALPPEERRRILDDYYRRELAPASVDRASFDKIFAAAWQAPPPVKAASERKQLNLTPVWL

TLGLVGVTAVALTGLVFWTNNLQDISTQDDATLIKSLRGGDPVREDEVALRRSLPDTGPEVWEIEKALEQIAEPLKDGGW

LEAKERTARVVDLLNRYEPQILARVPANARNRAVQAIAQVRANIRALEDKIQAKDITGSTLQARRTFWYLDLLELALLEE

FRPEVPAEYRNLPRLEGGWAMVRFTTDRGPFVALVDGFNAPVTAGNFLDLVQRGFYNGLKITRAERFNLVQTGDPSGKGT

GGFTDPATGKLRTIPMEVRPARKEAEITASQVIQRRVDYVQDRFELDPIKLREEMSNLHKRYIADDWKPVPYGKTIESYP

ALYYGPPGVFSMARYEKDPNSASSQFFISFSDPELNPTGKNLSDGKYANFGYITQGIRTLRQLKVGDTIQKAEILNCKAA

AFNEYRELPPAGSSMYATREGPATNLETSCLPLPAPRLVSGP

>ANACC00256 | K9Z9J9 | 1:1 ortholog | [Anabaena cylindrica (strain ATCC 27899 / PCC 7122)]

MFKFIKSWLKHSLKAILLVTILLGLSTAGWTPSSYAALPAGNAITDGKALLRYALPIDNKPVRKLQASLEDISNQLRANK

RWGAIANDLKQASRILDKPSQILASVPEERQPQAEAWITELKSGVTELQAVVKTKQKDPVLEGRAKLLNLVSLLEESMVK

EFPFEVPAEYSNLPQLKGRATIAFKTNKGNLTVVVDGYSAPVTAGNFVDLVQRGFYNGLEFTRSEESYVLQTGDPVGKEV

GFIDPKTGKYRAIPLEILVEGEKEPTYGITLEDAGRYLDMPVLPFSSFGALAMARPEGDVNGASSQIFFFLFEPELTPAG

RNLLDGRYTVFGYLIEGKEILDKLNADDKIESVTVVQGIENLVQPEAA

>NOSP700412 | B2J712 | 1:1 ortholog | [Nostoc punctiforme (strain ATCC 29133 / PCC 73102)]

MLHLSYPMLNLLKSWLKNSLMAILLVTIFLGITTAGWTPSSSAALPAGNAITDGKALLRYALPIDNKPVRQLQASLEDIS

AQLRANRRWGAISKDISKASRILDKPSQILTSVPTERQPQVEAWITELKSGVGKLEELANNKDKEQILQERGKLLNLVTQ

IEESMVKEFPFEVPAQYSNLPQLKGRATVEFKTNKGDLTVVVDGYSAPVTAGNFVDLVQRGFYNGLEFTRSEESYFLQTG

DPAGKDVGFIDPKTGEYRAIPLEVLVQGDKTPTYGITLEEAGRYVDMPVLPFSAFGAVVMARPESEVNGGSSQFFFFLFE

PELTPAGRNLLDGRYAVFGYLTEGKEVLDKLKAGDKIESAKVVQGIENLVEPQAA

>NOSS705020 | K9QZX2 | 1:1 ortholog | [Nostoc sp. (strain ATCC 29411 / PCC 7524)]

MIDGLRLVNNLMLHLSRPMFNLLKSWLKNSLIAILLVTIFLGINTAGWTPSSNAALPSGNAITDGKALLRYALPIDNKPV

RELQASLEDISTQLRANRRWGAVSKDLSKASRILDKPSQILASVPEERQPQAESWLTELKSGVEKVQELAQAKDKEQILQ

ERAKLLNLVSLIEESMVKEYPFEVPEEYSNLPQLKGRATIAIKTNKGDLTVVVDGYSAPVTAGNFVDLVQRGFYNGLEFT

RSEESYVLQTGDPPGKEQGFIDPKTGKYRAVPLEILVEGDEKPTYGITLEDAGRYLDMPVLPFSSFGALAMARPESQVNG

GSSQVFFFLFEPELTPAGRNLLDGRYSVFGYLTEGKDILDTLKAGDKIESATVVQGIENLIEPPAA

>ANAVT02273 | Q3MAQ2 | 1:1 ortholog | [Anabaena variabilis (strain ATCC 29413 / PCC 7937)]

MLNLFKSWLKNSLMAILLVTIFLGINTAGWTPSSNAALPSGNAITDGRALLRYALPINNKPVRELQASLEDISAQLRANR

RWGAVSKDLSKASRILDKPSQILTSVPEERQTQAETWINELKTGVAKVQEIAQSKDKEQVLLERAKLLNLVSLIEESMVK

EFPFEVPEEYINLPQLKGRATIAIKTNKGDLTVVVDGYSAPVTAGNFVDLVQRGFYNGLEFTRSEESYVLQTGDPPGKEQ

GFIDPKTGKYRAIPLEILVEGDKKPTYGITLEDAGRYLDMPVLPFSSFGALAMARPETEADGASSQVFFFLFEPELTPAG

RNLLDGRYSVFGYLIEGRDILDTLKAGDKIESATVVQGLDNLVQPQSA

>NOSA000565 | D7E1K7 | 1:1 ortholog | [Nostoc azollae (strain 0708)]

MFNLLKSWLKHSLRAILLVTILLGISTVGWTFSSYAALPSGNAITDGKALLRYALPIDNQPVRKLQASLEDISNQLRANK

RWGAVSRDLSQASRILDKPSQILASVPEERQPQASAWITELKSGIAELQEVVKNKQKDPIVEGRAKLLSLITLLEESMVK

KFPFEVPAEYSNLPQLKGRATIAFKTNKGDLTVVVDGYSAPVTAGNFVDLVQQGFYNGLEFTRSEESYVLQTGDPEGKEL

GFIDPKTGKYRAIPLEILVEGEKEPTYGITLEDAGRYLDMPVLPFSSFGALAMARPESDANGASSQVFFFLFEPELTPAG

RNLLDGRYTVFGYLTEGKEILDKLKAGDKIESANVVQGIENLVQPQPA

>CYASC00273 | K9YIG0 | 1:1 ortholog | [Cyanobacterium stanieri (strain ATCC 29140 / PCC 7202)]

MKSNWWQRLTKIIVATVLIFTGAIALTPTPWQNAHAILAQGDAITDPEAILRYALPIENKDIREVQSNIEKIEKDLRSKR

WKRVEKEVRNAAFALKLHGDDIAQDVPREFAERSEELVKDITGDVEALQELVAGQKRDEILALREEILDHITELEEGMVR

GFPFEVPQEYANLPQLKGRAKVKITTTQGDLVIEVDGYSAPVTGGNFVDLVQRKFYDNLPFIRAEDFYVIQTGDPEGKEV

GFIDPKTDKYRAIPLEVLVKGESEPIYGLTTEDVGLYLAQPVLPFNAYGAVALARPTTDPNGGSSQFFFFKFDTEVTPPG

YNLMDGRFAVFGYVTEGAEVLEKLSPEDKMLSAEVIEGAENLVSPQA

>MICAN01200 | B0JTU3 | 1:1 ortholog | [Microcystis aeruginosa (strain NIES-843)]

MKIKTRLRESGKFFLKSGASIALALFLILSLFTVKGTAPALAVLAQGDAVTDPTAILRNALPIDNKPIRQVQQSIEDIAK

HLRAKRWSPIKKDVKDANYALSTKSKAILDSVPEASKSQGEELIEKLKTGVADLDTAVEAKDKEAVWSTRRELLNNITAL

EELMVVGFPFNVPPEYANLPQLRGRATVEMQTTKGDLTIVVDGYSAPINGGNFVDLVQRGFYDGLPFIRSEDNFVVQTGD

PVGAEEGFIDPKTKQYRSIPLEILIKGEEEPVYGNTLEELGIYLPSLALPFNAFGALALARPDTNPNGGSSQFFFFKFDN

ELTPPGFNLMDGRYSVFGYLVQGKEVLEELTDQDKIITAKVLYGLNNLIQPS

>CYAA500498 | B1WP74 | 1:1 ortholog | [Cyanothece sp. (strain ATCC 51142)]

MVNKNSENDHLSSSLKLMKLKQSFWSDVRSRLEHWCKKTIVAMIVVTLSINLCGATWSQFGTDSVLISVLAQGNAITDPE

AILRYALPIENEPIRKVQDAIEDISNHLRGKRWPPIAKDVKTASFVLTLRSDEILEGVPGDRQFQAETILEDIKTGVREL

QEAVENKDKEQVWIKRRNVLDNIGEIETLMVEGFPFEIPEEYADLPQLKGRATVEIETTKGNLTIVVDGYNAPVNGGNFV

DLVQRGFYDGLSFIDLEDSFAIQTGDPPGKEEGFIDPETGEYRAIPLEVRVKEDEEPIYGATLEEMGIYLPDLVLPFNAY

GAVALARPSLDPNGGSSQFFFFKFDSELTPPGFNLMDGRYSVFGYLVDGKEVLENLTKKDKIISAQVIDGLDNLVKPKNS

>ARTPN02321 | D4ZPR7 | 1:1 ortholog | [Arthrospira platensis (strain NIES-39 / IAM M-135)]

MLNLSESIMNTCKRWLKTGLIALLLCVLSVGLSAAWWDGGSSTPKRESVLPAGNAITDGKALLRYALPIDNEPIRKFQSS

LEEIAGRIRGKRWSPIKGDITTAARILSINEGDILASIPDDRQTEAKAIIEELRAEIEVLRSALDIKDGETLLETRAQML

SQVTQLEEMMVGEFPFEVPAEYANIPQLKGRATVKMTTSEGDITLVVDGYSAPVTAGNFVDLVQRGFYDGLEFIRSEESY

VLQVGDPPGPEEGFIDPDTGEYRAVPLEILVRGDDEPTYGITLEAAGRYRDQPVLPFSAYGTVAMARPESNPDGGSSQFF

FFLFEPELTPAGLNLLDGRYSVFGYVTEGKEVLENLTQGDAIASAKVIQGLENLVQPKK

>TRIEI01066 | Q116D7 | 1:1 ortholog | [Trichodesmium erythraeum (strain IMS101)]

MVNLRNFVMNTCKRLLKTAILGLLLITLSIGISAAWWNGGMTTTRQSRLPQGDPITDGRALLRYALPIENEPVRKIQSSL

EDISNRLRGKRWTSVVSDIGTASRVLSINKPKLLASVPESKQPEAEEIIAILDQGIIDIKEVAEAKDGEQVLEQRAKLLT

LVGQLEQLMVVGFPFEVPSDFSNLPQLKGRATIDMETNKGSLTLVVDGYSAPVTAGNFVDLVQRGFYDGLEFIRSEQSYV

LQAGDPPGPEVGFIDPDSGKYRNIPLEILVQGDNEPIYSFTLEEIGRFRELPVLPFSAYGAVAMARPEAENDGASSQFFF

FLFQPELTPAGVNLLDGRYTVFGYVVAGKKVLEELNKGDKIISAKVVAGFENIVEPEA

>LYNSP02447 | A0YPV7 | 1:1 ortholog | [Lyngbya sp. (strain PCC 8106)]

METCKRWLKTGVVLLLLCSLSIGLSGAWWDFGGSSSQPKRESVLPQGNAITDGKALLRYALPIDNEPVRKMQRSLEEIAT

RIRGKRWSPIKSDIAIASRVLTTQESELLASIPSDRQPEAEAIISQLQGGIESLREALEVKDGEKLLETRADVLAKVSEL

EELMVEEFPYEVPDEYANLPQLKGRATVEIKTSRGEITLVADGYSAPVTAGNFVDLVERGFYDGLKFDRAKDAFYLQVGD

PPGKEEGFIDPKTGKERDIPLEILVRGETEPIYGYTLEEIGRYRDKPVLPFSAYGTVALARPEPLPNGGSSQIFFFLFEP

ELTPAGLNLLDGRFAAFGYVTKGKEVLENLQQGDQIESAKVIKGAENFIKPQDA

>STAC703975 | K9Y016 | 1:1 ortholog | [Stanieria cyanosphaera (strain ATCC 29371 / PCC 7437)]

MKTKTKSWYQLLLKTTTAAILALLMFLSSSLVNHQSDALAGSRKSMLAQGDAITDPLAILRYALPIDNDTVRKIQADLED

ISNQLRGKRWGTISRDVKDAKNLLNYRREQLLASIPEERQSQASALLEQINTGISKLESELEAKDKNAISETRAEILNQI

GQLEALMVTGFPFAIPEEYTNLPQLLGRATVEITTTKGDLTIVVDGYSAPINGGNFVDLVQRGFYDGLNFIRAEDFYVLQ

AGDPPGEEEGFIDPKTNQYRAIPLEVLVKNDPEPVYGVTLEELGRYLDQPVLPFNAYGAVALARPANDPNGGSSQFFFFK

FDTELTPPGYNLMDGRYSVFGYVVKGKEVLEELTADDKIISAKVIDGLDNLVQPKAG

>ACAM100532 | B0CD62 | 1:1 ortholog | [Acaryochloris marina (strain MBIC 11017)]

MYSLHSWLKRTALLLLTLVFSLGLSGPAWSAPFLPLSVLPTANAVKDGSSILRFALPIDEPYIREIQNAVEGTTPQIRGK

RWPEIRKGLGKAKRTLERHRSDILAAVAPDQQAVASEQLDFIAQALLDLEDAVAAKDIDQFNTIRKPLADRVGIIEEAML

TEFPFEVPEEYQDLPQLKGRATIAITTNKGDMTVVVDGYSAPVTAGNFVDLVQRKFYDGLKFTRAEESYVVQTGDPDGPD

AGFIDPDTGEYRAIPLEIMVKGDSEPIYGSTLEDLGIYLDEPVLTFSSYGTMAMARPGDDANGGSSQFFFLLFEPELTPA

GSNLLDGRYAVFGYVVDGQDVLGTIKPGDVIESARVIKGAENLV

>CHAP603372 | K9UJP7 | 1:1 ortholog | [Chamaesiphon minutus (strain ATCC 27169 / PCC 6605)]

MRTGKISILSRWRNWLQTGIVAMLSISLSMGLSAATLLPSGNAITDGQSILRYALPIDNKPVRELQNSLEDIANHLRGKQ

WSTISNDVKKAAAILKNKQADILTSVPAPKQAEAQTLLDDIQTDVEALAIATAAKDKPQVQAKRTEALGKIGSIEASMVT

DFPYTVPAEYSNLPQLKGRATISIKTNKGEIGLIVDGYNAPVTAGNFVDLVQRGFYNNLAFTRAENDYVLQIGDPPGDEV

GFIDPDTKKYRAVPLEVMVKGDKEPMYGITMEDAGRFREEPVLPFSSYGALAMARPEGDPNGGSSQVFFFLFEPELTPAG

RNLLDGRYTVFGYAISGQEVLGQLKPNDKIESAKVIEGADLLKT

>SYNY302251 | PPI3_SYNY3 | 1:1 ortholog | [Synechocystis sp. (strain PCC 6803 / Kazusa)]

MQIIKTPLGIITRRGLQLSLLSLLLTMLSLTWAMPGWSLPLNQPMLLGALAQGNAITDPNAILRYALPIDNPEVRRLQDS

LEDISNHIRAKRWPAIKKDVRAANLTITLKEDKILAGVPADRQPEAETLLGSIKTDLTALTEAVEAKDKEQVISFRKSAL

TAIGDLEALMVTDFPFAIPEEFANLPQLKGRATVEMTTNKGPLTIVVDGYSAPINAGNFVDLVQRKFYDGLPFIRSEDFF

VTQAGDPPGPEAGFIDPQTKEYRAIPLEILVKGEEGPIYGMTLEDAGMYLPELALPFNAYGAIALARPETEPNGGSSQFF

FFKFDTELTPPGFNLMDGRYSVFGYVVDGKETLEQLSEGDKIVSAKVISGADNLVNGNS

>PROM400026 | A9B9I1 | 1:1 ortholog | [Prochlorococcus marinus (strain MIT 9211)]

MTKKILLLAILTLFIPFALVWDKPAIAALPNGNRIKDPYAILRNALPINQKDLREIQNGLEETSDLVRGGRWPAISKATS

RSKSLLNNRKKKIIDSIPNTSKKQGEQIIISLKEDLENLNEQATAKNKAKFIDIRRKALQEIGDLESLFLPGEFPYKIPN

EFDNLPRLLGRATVSIETTKGKMNAVIDGYNAPLTAGAFVDLVEKGFYDGLPMNRAEEFFVLQTGDPKGPEIGYIDPETK

QERNVPLEIRVDGQSETIYNETFEELGLYTSTPTLPFATLGTLGWAHSEQALDDGSSQFFFFLYEAELNPAGRNLIDGRN

AAFGYVIEGNEILNELGVNDQIISIKVLNGSEKLKSHA

>CYAGP02198 | K9P908 | 1:1 ortholog | [Cyanobium gracile (strain ATCC 27147 / PCC 6307)]

MDRRRRRIGFTTTLAARLGAALTLVLTLLLAVPAAWAYLPQGNAVSDPTALLRNALPMEQGDLQGLQHRLEDTSDDLRAK

RWSSLTGSVRRAESQLGGSRERILASFDPADRPTAEALLADTAVHLQELAAAGEAQDRDRFLAARREALADIGQAEALLV

GPFPFTIPAEFDGLPRLLGRATVRLTTTKGDLTTVVDGYNAPLTAGAFVDLVQRGFYDGLPFNRAEDFYVLQTGDPAGPA

TGYVDPISKQERTVPLEIMVPGQEAPFYNQTFEDLGMFKAEPVLPFASKGTLGWAHSDEALGDGSSQFFLFLFEPELTPA

GLNLIDGRYAAFGYVVDGFDVLEELTADDGIVKATVVEGADNLRPHA

>SYNP601544 | A0A0H3K3E3 | 1:1 ortholog | [Synechococcus sp. (strain ATCC 27144 / PCC 6301 / SAUG 1402/1)]

MRELDAFAGSIMLSLARRVAAWLPGLLALSLALLLPMLAVAPAAIAGLPPGNAITDGRALLRYSLPIDSPDIREIQKDIE

GLSDNLRAKRWAPIERNLKHVSKVLNLRPKNILAAVPEERRSQAEELLAELKTDLSKLEEATAAKNKPDVQAARNHFLAV

VGEIEELMVERFPFEVPEEYRNLPRLEGRATIAVETTQGDLTLVVDGYSAPITAGNFVDLVQRGFYNGLPFTRAEDFYVL

QIGDPVGPETGFIDPKTKQERQIPLEILVEGDREPVYGSTLEELGRYTDNPVLPFSAFGTLGWARPSDNLNGGSSQFFFF

LFEPELTPAGLNLIDGRYAAFGYLVDGKDVLEKLRPEDKILKATVVSGAENLIQPS

>THEEB00798 | Q8DKN8 | 1:1 ortholog | [Thermosynechococcus elongatus (strain BP-1)]

MKSSPLEGDRLVRYTEGRQSWEETVKRRKRWLAVLVILLCCWGTFLLPPLAIALPVAPPLIAALPQGNAITDPKALLRWA

LPIDNPTVRELQKDLEQISFWVRGKQWSKIVSNISKAKTIIRDRTADLLQSIPAEKQEAAKTLLAELETSLDTLQEAAKA

KDRSQLLPAKAAALDKVGELEAMMVQNFSYTPPKGYEHLPRLLGRATVEMETTKGKLTIVVDGYSAPLTAGNFVDLVQRH

FYDGLPFTRAEESYVLQAGDPPGPEVGFIDPKTNQYRAIPLEILVEGDKYPLYGITLEDAGRYLEHPVLPFSAYGTVALA

RPNDDPNGGSSQFFFLLFEPELTPAGLNLLDGRYAVFGYVVEGEETLRQLRQGDKILSAKVVDGLENLVQPA

>VITBC11537 | A0A0G4EJ74 | 1:1 ortholog | [Vitrella brassicaformis (strain CCMP3155)]

MGVRRSSVAICAAALLGLSEVAWTFRVQHSFSDATRLTRTPSLRRPSLPEQQSTGPAASENAECTSRRGYAEEAARAAVG

IAALSPLLPSFPSNAAKSWQGGLNAPSAAGSRVNKDADSLLRYGLPIQDKDVTALQVELEAVKGDIPVKRFDSAVNHLNK

ARVLIRDKEASLLKAIRPKSEDKAKELLASIGASVDPLIEAIREPASRGSPQEAAKLDKCLELQGGIADKMTALMALMVP

ENYKVPVPQEFASLPQLQGRAKVEMVVKKADGSKFDLEGRLYDEAKLVMVVDGWNAPVTAGNFVDLVSKGFYDSMKISRS

DGFVVQTGKPDSGPEGYVPTGAKEPRRVPLEIFVKGDNAPMYGETTEEDGRGFAATTLPFQAYGALGMARSEFDNNSGSS

QFFWLLFDSDLTPAGKNFMDGRYACFGYTVSGADFLRDIRVGDTIVSARVVEGIDKLQKTGKSESPSYAV

>EMIHU17963 | XP_005788109 | 1:1 ortholog | [Emiliania huxleyi]

MRLTPPPSTFAGAAARSLSAAALAALLSVSSITPSVPPAFGPAAAQAKELASGSGSRVNKDPISLLRLALPRVSKEAREL

QAGLEECQDNLARLNGQVASSALQKAKVAAGKSGAMLKSVPPAKAAEAQAALDTINAAMDEVGAKIAASRQGEALAATER

ALGAVTQLQEAMASGYAQPSPPKEFASLPYLKGRATVDLVLQPGKEHGQFDVEGKLYKQLEVKMVVDGYTSPITAGNFVD

LVRRGFYNGMHIQRSDGFVVQTGDPSNELEGSGADAKKNGYVEGGKVRKIPLEVMVRGDKDPIYSATFDDDGRGGYAAAM

PFNSYGALGMAREEYDPDSASSQWFWLLFDSDLTPAGKNLLDGRYACFGYTVDGARLLSDVKEGDIIASAKVSSGESNLV

LPTAPALAVAAE

>CYAME00775 | CMK307C | 1:1 ortholog | [Cyanidioschyzon merolae]

MITALRRYAFEHRNEESVFLLGCRAMWSQLAPVHSLQRLERKFRARCRSRKPLLRLHHGSGDGEFSVSRRELLKHSAAAL

TAAALSQLGVAALTERHVVDEAAAANRTLSGASGVVNKDPESLLRWALPIDNEHLRQLQTELEATLRELRQKKWSQISSH

VRKASQMTSRNANDLLADVSEAKTAEAQRALSTLQSMLGSLQASADAKDAENFESMAKDALRQVGTLEELSVKGFPFQVP

EEYSHLPQLLGRATVEFVLRKSGEEGSPKQFDIDGNLYDRARLVMVVDGYSAPVTGGNFVDLVSRGFYNGLRIIRSDGFV

IQTGDPEPEGKIHGFIDSKTNQERTIPLEIFAKGDPMPLYGTTLEDDGRGGQATVLPLTAFGSLCFARNEFEPNSGSSQF

FWFLFEPDLTPAGRNLLDGRFAVFGYTTEGAFFLRDVKVGDIVESAKVVQGLENLRQPTA

>GALSU02599 | M2XYR0 | 1:1 ortholog | [Galdieria sulphuraria]

MIAYQFCFVASRDWGCTRLYSSKCFRYGYKNGARSGRKQSNAHCSSTRINCIARNFQTSLQNLVKLLENSFCQERNRQSR

ATVILVLASCLVLSPIQFNSVDGSFGSVPALAAKNRFAPGTNEPVVKDPEAILRNALPIENKTIRKIQHSLESIPKDIRT

RSWSKIEQEFKQANSLLDKQKDDILSNILESRRTEARNLLDNLISHKLPSLRQSISQRDSEKITLFNVDILRDIAVLEED

MVKQFPYEVPKEFDHLPQLKGRAVVEMTIKKANNQKFDLDGQLFDKGQVTLVIDGYSAPVTGGCFVDLVNRRFYDGMKVI

RSDGFVIQTGDPEGPDDGFVDPQTKRKRLIPLEVFAKGDEAPTYGITLEDDGRGAAATVLPFTSYGTLAMAREEFDANSA

SSQFFWLLFDPELTPAGRNLLDGRYAVFGYTINGQEYLQDFKVGDYVEQAKVTKGIENLKQPAE

>CHOCR00630 | R7QPR7 | 1:1 ortholog | [Chondrus crispus]

MASPPPPAFALAALPTSSAPSSAFFAPPLTTSRPTSRPHSATRHTIRAVHPPNKAPPPASSTASKAIAAFAAASILLSAP

SLPPAAHAPFVAPPFVAPAHAARGAKGGGASFLSASGDVNKDPESLLRWSLPISNKPVRELQTELEAAVTDLRGLKWAKV

DNHVKAASRLLNNQTNKILVAVPAAARDDASAVLASAADRLHDVEAAVTDKSTDKLTKACKDVLGDIGRVEEMMVAKFPF

QVPDEFSNLPQLKGRATVEMLVKKDGDEPFDIEGTIFKEGKMTLVLDGYSAPISAGSFVDLVNKGFYNDNPVFRSDGFII

QAGKPKNGEGFTDATGKVRSIPLEIFARGDKEPTYGITLEEDGRGAQGTVLPFTSYGTLAMARAETEANTASSQFFWFLF

EPDLTPAGRNLMDGNWAVFGYTTKGENFLKGLQKGDRIVEAKVVDGLQNLVTAK

>PHATC08487 | EEC50201 | 1:1 ortholog | [Phaeodactylum tricornutum (strain CCAP 1055/1)]

MKLTSAAVLGWLALTASTAAFAPRQPTMRTTASTTSLEASTPWQDWGKMTVATALVTAALWSTPVNPETQSAFFATPPAV

AKEMASGSGSRVNKDPESLLRLGLPIKNKEVRKLQASLEGIKLDIGSKRKLAAADAVKKSKAFLVGKDADKVKAACRDPS

ICGAIIKDMVVKLDPLADALKESQEAFQGSEQERTALDKAYAAQQVVVDQLTALEEQLIPAGYKRPVPEEYSDLPQLHGR

ATVEMLIDKADKSTFDYNGKIVPQAKLVMVIDGYTAPVTGGNFIDLVQKGFYNKMEIQRSDGFVVQTGDPAGEADGYVAK

GSKTVGNGKHGERLIPLEILVRGDSTPVYESTIEDEGRGGEATVLPFSAYGAMGWAREEYDPNSGSSQFFWLLFDSDLTP

AGKNVLDGRYPCFGYVTEGADFLSSILEGDVIVSAKVVDGGENLVQPQS

>ECTSI04780 | D8LET7 | 1:1 ortholog | [Ectocarpus siliculosus]

MSPCKSTACVALAAVSTLIAGVSDAFVVPSASVLGASRSNSRAPGSSSSSSLSMGADGGTTRGDFGKQTIATLLAGVTAA

ANFESANAANKLQVSSTASGSRVNKDPISLLQWGLPIDCKPARQLQEVVEAAKLDLFQKNWSRAVSNCSKAKSAMSGNKA

AILKAVGSSNEAGATKLMADIDMGLGALQRIVQDDGAGVVLEKAKLDEAYPVHATIVSNVGNLEAMMVPPDYTKKLEANI

PGDFDSLPRLLGRASVEFVMEKPKGEKFDVAGVLYDKVRLTMVLDGFTAPVTAGTIIDLVQRGYYKNMPITRSDGFVVQT

GDPDPEGNVHDFFPPGSDTARKIPLEIAVTEDKLPMYSVTTEDDGRGYAATKLPFQAYGALGMARSEFEADSASCQFFWL

LFDSDLTPAGKNLLDGRYAEFGYTVDNQELLADVKEGDIIKSAKVISGMENWKQSA

>CHLRE13497 | A8IRU6 | 1:1 ortholog | [Chlamydomonas reinhardtii]

MALANLSRGQASVGAIGTRKAVPAVRRVACQAYKGDEEARNPLQKAAIGLATAAAVVLGTFAPLVTPAPADAILVASDPV

KNAAAILRNALPIDNKPIRQIQKDLEGISEALRIPGSKSLGPVARAVRASQDVLTRQKDAIIKDFAPEKKAFGLANIDKL

TEALTEFQAIVEAKDKQAVPLKQREALSYVGNIEEAMVKGFPFQVPKEYADRPLLLGRATLEMKVKCAQTPEGPQTFTQT

VVLDGYNAPVSAGQFADLVARGFYDGMEVQRADGFVVQTGDPDGPADGYVDPKTNEIRRVPFEVRVQGDKEPIYDFTLED

LGRVNEQPVLPFNAYGTLAWARNEFENNSASSQVFFLLKESELTPTGSNLLDGRFAVFGYITQGQDALADFRVGDKIEYI

KFVDGKENLKNSP

>VOLCA13980 | VOLCADRAFT_108119 | 1:1 ortholog | [Volvox carteri]

MPALMNLTSHSRAARVASACSVSRTQRASSRICRAVNQISSPSDEPSTRSPLERAAIGLATAAAVVMGTFSPLVLEPPPA

HAILVASDPVKNAQAILRNALPINNKPIRQIQKDLESISEALRIPGSKSLGPVSRAIRASQDVLTRQRDAIVKDFAPEKK

DFGLENIEKLQVALNEFQATVEAKDKQAVPIKQREALTYVGNIEEAMVKGFPFQVPPEYADRPLLLGRATLEMKISCRET

PEGPQTVVQTVVLDGYNAPVSAGQFLDLVLRKFYDNMEIQRADGFVVQFGDPEGPADGFVDPKTGDIRRVPFEVKVIGDK

EPIYDFTLEDLGRVNEQPVLPFNAYGTLAWARNEFENNSASSQVFFLLKESELTPTGSNLLDGRFAVFGYITQGQDALAD

FKVGDKIEYVKVIDGAQNLKNGPSA

>CHLVA00162 | E1Z2A2 | 1:1 ortholog | [Chlorella variabilis]

MPVQLEQAGSLLRNALLGAAAAALVGGGAAPDALAPPAWAGIGGGAVTNAKALLRYSLPVDNKPIRQIQRDLEAISDDLR

VPGSKSLGPIGRRVRAASSVLEREGSAITAAFAADKKAAGLAAVEGLKQSLSDFQGVLDAQDKQAVPLVQQQCLDYVGAV

EEAMVGAFPFEVPKEYADLPQLKGRATVEMKFKFTDARENNATGGTMTMVLDGYNAPVSAGDFVDLVRRGFYTGMEIQRA

DGFVVQTGKPDGSSQGFEVDGKVRTIPLEVMVKGDKVPVYEETLEDLGRFRESPVLPFNAFGTMAIAREEFDPNSGSSQF

FWLLKESELTPTGANLLDGRYGVFGYVTEGSQLLKEMQVGDKIVSAKVVDGEENLVLPK

>OSTLU02326 | A4RWY9 | 1:1 ortholog | [Ostreococcus lucimarinus (strain CCE9901)]

MAIAATACVSAPTLEAFASPVTPMEIGQVAGLEANPITNAKALLRNALPVSNKPIRAIQKKLESISDDLRVPGVKFSGVE

SSVNGSLKIVKEQRGKIVEALASGKKADGEQALKELESVLTDFQVIVAQKDKQEVPLQQQEALSLVGRIEEDMIDGFPFD

VPAQYADRPLLKGRATVDMLVKIKDNANTDGGVLKIVLDGYNAPVSAGNFADLINLGFYDKMPIQRSDGFVVQSGKPKGG

DGFKLGGVERTVPLEIMVQGDKVPEYELTLEELGRYRDQPVLPFNAFGTLAMARREAEPNSASSQFFFLLRESELTPSGT

NILDGRYSIFGYVVENQELLRDLKVDDEIISMKIVDGAENLVNETKPAVDQAPIEAPSE

>MICCC08253 | C1FEJ6 | 1:1 ortholog | [Micromonas commoda (strain RCC299 / NOUM17 / CCMP2709)]

MFALIYSNVAAKTTSSNPTSSVSSHHPLSRRIAIVRATFSDSDRSRAAATFPEVAKQFGAALFASVVMTGPSSASMLDHP

SVIGETAGLEANPVTNARALLRNALPIDNKQIREVQRKLESISEDLRVPGVRFSGVESSVNGAYKIVTTDYQKILASVAP

DKLRDGSAILAELRRELEDFKVIVSNRDKQEVPYAQQRALDLVGRVEEEMIDSFPFEVPAPYDTAPLLKGRAKVEMEVAV

KDNPNIDEGTMTIVLDGFNAPVTAGNFVDLVQRGFYDGMDIQRSDGFVVQSGKPSAKSQDGFIDPASRKERTIPLEIMPK

TRGKNEAPVYEFTFEDVGKYRDEPALPFNAFGTLAMARREAEPNSASSQFFFLLKESELTPSGTNILDGRYSVFGYVIDG

QEILRELKAGDLIKSIKIVDGAEYLVNGTRDNTATPDSITTTAETN

>KLEFL13170 | KFL_000140480 | 1:1 ortholog | [Klebsormidium flaccidum]

MAGVARAASLGLPTACGLGSFRKQGDAAGRRAATSKLHCQRDAFVNSPLLLPPETGKHAGLSRKEARAALKRDEGHEGAS

AKEGLFSALKRSAAAAALALTLASPSQSLATTLSPPPLNPVLGDVSVLISGPPIKDARTLLRNALPIQGKAVKEVQAALE

GITESLRLPGTKGFDQVSGAVRKASGALNQNLDGILNEVPEAKRADASQLVDQLRAGLSDFQQVLQGSSKEAVATKQKEL

LSIVGNIEEAMVQRFPFEIPAEFAGKPWLKGRATVEIKLKVKDNANLSKVTMTAVVDGYNAPITAGNFVDLVERRFYDGM

DIQRADGFVVQTGDPDGPSEGFVDPNTGKLRTIPLEIKVIGDKLPIYGATLEDEGRYKAQTRLPFNAFGTMAMAREEFDA

DSASSQIFFLLKESELTPSQANILDGRYSVFGYIVDNEDFLADLKVGDSIESMRVVQGIENLQNPSYK

>MARPO00660 | Mapoly0001s0360 | 1:1 ortholog | [Marchantia polymorpha]

MYASMGMQACAPSALSSSCAASLRTQCANSSTTTAASSSSAKVLGVDVSSSKSPWKGSSVQDSRSSSSSSRRSRGCCACQ

ASSGSEKASLLQKIGDSVKNCVLCAALAAAVALPQFPVQPSVATESLPGPVYSELGVLIAGPPIKDANALLRYALPISNK

PIKEVQRTLEGITDALKLPGVKAIDPVERDVRQASRLINQNKDQILADVAESKKEEARAAMSRLSDGLLEFQSLLEQKDR

AGILPKQKECLEYVGQVEEAMVEKFPFEVPAEYAKMPLLKGRAKVEMKVRVKDNPNLKNAVFEMTVDGYNAPVTAGNFID

LVERRFYDNMEIQRADGFVVQTGDPDGPAEGFVDPGTGKLRTIPLEIMVEGEKEPIYGGTLEELGRFREQTKLPFNAFGT

LAMAREEFEPDSASSQIFWLLKESELTPSNANILDGTYSVFGYVTDNPDFLADLKVGDVIESIRVTSGLDNLVNPSYKVG

GA

>ORYSI34241 | B8BAB3 | 1:1 ortholog | [Oryza sativa subsp. indica]

MAAALAFPTCCCCRRPSLRPSAGRRGRRPVARCALPSSEKNSFSWKEYAISVALSAGLITGAPTLGWPAHASPLEPVIPD

VSVLISGPPIKDPGALLRYALPIDNKAVREVQKPLEDITDSLKIAGVRALDSVERNVRQASRALSNGRNLILGGLAESKR

ANGEELLDKLAVGLDELQRIVEDRNRDAVAPKQKELLQYVGTVEEDMVDGFPYEVPEEYSSMPLLKGRATVDMKVKIKDN

PNLEDCVFRIVLDGYNAPVTAGNFLDLVERKFYDGMEIQRADGFVVQTGDPEGPAEGFIDPSTGKVRTIPLELMVDGDKA

PVYGETLEELGRYKAQTKLPFNAFGTMAMARDEFDDNSASSQIFWLLKESELTPSNANILDGRYAVFGYVTENEDYLADL

KVGDVIESIQVVSGLDNLANPSYKIVG

>BRADI16984 | I1I6Q4 | 1:1 ortholog | [Brachypodium distachyon]

MAAALASSRCCCRPSLLPTGRGRRSVARCALSGEKRNSFSWKECALSVALSVGLITSPPTFGWSAHASPLEPVLPDISVL

ISGPPIKDPGALLRYALPIDNKAIREVQKPLEDITDSLKVAGVRALDSVERNARQASRALSNGRSLILDGLAESKRANGE

ELLDKLAVGLEELQRIVEDRNRNAVAPKQKELLNYVGTVEEDMVDGFPYEVPEEYSSMPLLKGRATVDMKVKIKDNPNVE

DCVFRIVLDGYNAPVTSGNFIDLVERKFYDGMEIQRADGFVVQTGDPEGPAEGFIDPSTGKSRTIPLEVMVDGDKAPTYG

ETLEELGRYKAQTKLPFNAFGTMAMAREEFDDNSASSQVFWLLKESELTPSNANILDGRYAVFGYVTENEDYLADLKVGD

VIESIQVVSGLDNLVNPSYKIVG

>HORVV174363 | A0A287WPI4 | 1:1 ortholog | [Hordeum vulgare subsp. vulgare]

YMTLNFLSWIYFEECMLWKKGNSFSWNKCAISIALSVGLITCPPTFGWSAHAFPLEPVIPDISVLISGPPIKDPGALLRY

ALPIDNKAIREVQKPLEDITDSLKVSGVRALDSVERNVRQASRALTNGRSLILTGLAESKRENGEKILDKLAVGLEELQR

IIEDRNRNAVAPKQKELLNYVGIVEEDMVDGFPYEVPEEYNNMPLLKGRATVDMTVKIKDNPNVEDCVFRIVLDGYNAPV

TSGNFVDLVERKFYDGMEIQRADGFVVQTGDPEGPADGFIDPSTGKSRTIPLEIMVDGDKAPIYGETLEELGLYKAQTKL

PFNAFGTMAMAREEFDDNSASSQVFWLLKESELTPSNSNILDGRYSVFGYVTENEDFLADLKVGDVIESIQVVSGLDNLV

NPSYKIVG

>AEGTA27857 | M8CD05 | 1:1 ortholog | [Aegilops tauschii]

MGIFAKHESGGTRALQKGNSFSWKECAISVALSVGLITVPPTFGWSAHAYPLEPVIPDISVLISGPPIKDPGALLRYALP

IDNKAIREVQKPLEDITDSLKVSGVRALDSVERNVRQASRALTNGRSLILSGLAESKRANGEKTLDKLAVGLEELQRIIE

DRNRNAVAPKQKELLNYVGTVEEDMVDGFPYEVPEEYNNMPLLKGRATVDMTVKIKDNPNVEDCVFRIVLDGYNAPVTSG

NFVDLVERKFYDGMEIQRADGFVVQTGDPEGPAEGFIDPSTGKSRTIPLEIMVDGDKAPIYGETLEELGLYKAQTKLPFN

AFGTMAMAREEFDDNSASSQVFWLLKESELTPSNSNILDGRYSVFGYVTENEDFLADLKVGDVIESIQVVSGLDNLVNPS

YKIVG

>WHEAT56841 | A0A1D6CYY9 | 1:1 ortholog | [Triticum aestivum]

MAAALASSRCCRRPSLLTIDRRRSSVARCALSGGKGNSFSWKECAISVALSVGLITVPPTFGWSAHAYPLEPVIPDISVL

ISGPPIKDPGALLRYALPIDNKAIREVQKPLEDITDSLKVSGVRALDSVERNVRQASRALTNGRSLILSGLAESKRANGE

KTLDKLAVGLEELQRIIEDRNRNAVAPKQKELLNYVGTVEEDMVDGFPYEVPEEYNNMPLLKGRATVDMTVKIKDNPNVE

DCVFRIVLDGYNAPVTSGNFVDLVERKFYDGMEIQRADGFVVQTGDPEGPAEGFIDPSTGKSRTIPLEIMVDGDKAPIYG

ETLESLYGHAFQELGLYKAQTKLPFNAFGTMAMAREEFDDNSASSQVFWLLKESELTPSNSNILDGRYSVFGYVTENEDF

LADLKVGDVIESIQVVSGLDNLVNPSYKIVG

>ERATE17318 | maker-scaffold7841-a | 1:1 ortholog | [Eragrostis tef]

MQKRNSFSWKECAVSVALSVGLITGAPTLGWEAHASPLQPVVPDVSVLISGPPIKDPGALLRYALPIDNKAIREVQKPLE

DITDSLKVAGVRALDSVERNVRQASRALSNGRNLILDGLAESKRANGEELLDKLAVGLEELQRIVEDRNRDAVAPKQKEL

LQYVGTVEEDMVGGFPFDIPEEYSNMPLLKGRATVDMKVKIKDNPNLEDCVFRIVLDGYNAPVTAGNFIDLVERKFYDGM

EIQRADGFVVQTGDPEGPAEGFIDPGTGKPRTVPLEIMVDGDKAPVYGETLEVRNGYFPPSFIHFIAARNFGSYI

>SORBI02398 | A0A1Z5RAB0 | 1:1 ortholog | [Sorghum bicolor]

MAALLASSRCCCSRPSLPPLPTRGRRSVARCALSGGEKRNSFSWKECAVSVALSVGLINGAPTLGSPAYASPLEPVLPDV

SVLISGPPIKDPGALLRYALPIDNKAIREVQKPLEDITDSLKVAGVRALDSVERNIKQASRALNNGRSLILAGLAEPKRA

NGEELLNKLAVGLEELQRIVEDRNRDAVAPKQKELLQYVGTVEEDMVDGFPYEIPEEYSTMPLLKGRATVDMKVKIKDNP

NVEDCVFRIVLDGYNAPVTAGNFIDLVERKFYDGMEIQRADGFVVQTGDPEGPAEGFIDPSTGKIRTVPLEIMVDGDKAP

VYGETLEELGRYKAQTKLPFNAFGTMAMAREEFDDNSASSQIFWLLKESELTPSNANILDGRYAVFGYVTENEDYLADVK

VGDVIESIQVVSGLDNLVNPSYKIVG

>MAIZE04295 | B6U5I1 | 1:1 ortholog | [Zea mays]

MAAALASSRYCWSRPSLPPQPTRGRRSVTSCALSGREKRNSFSWRECAISVALSVGLITGAPTFGPPAYASSLEPVLPDV

SVLISGPPIKDPGALLRYALPIDNKAIREVQKPLEDITDSLKVAGVRALDSVERNVRQASKALNNGRSLILAGLAEPKRA

NGEELLNKLAVGFEELQRIVEDRNRDAVAPKQKELLQYVGTVEEDMVDGFPFEIPEEYSNMPLLKGRATVDMKVKIKDNP

NMEDCVFRIVLDGYNAPVTAGNFVDLVKRKFYDGMEIQRADGFVVQTGDPEGPAEGFIDPSTGKIRTVPLEIMVDGDKAP

VYGETLEELGRYKAQTKLPFNAFGTMAMAREEFDDNSASSQVFWLLKESELTPSNANILDGRYAVFGYVTENEDYLADVK

VGDVIESIQVVSGLDNLVNPSYKIVG

>SETIT21970 | K3YHT3 | 1:1 ortholog | [Setaria italica]

MAAALASSRCCCSRPSLPPLPTRGRRAVARCALGGGEKRNNFSWKECAISVALSVGLITSAPAIGSPAYASPLEPVLPDV

SVLISGPPIKDPGALLRYALPIDNKAIREVQKPLEDITDSLKVAGVRALDSVERNVRQASRALNNGRDLILAGLAESKRA

NGEDLLNKLAVGLDELQRIVEDKNRDAVAPKQKELLQYVGTVEEDMVDGFPFEIPEEYSNMPLLKGRAAVDMKVKIKDNP

NIEDCVFRIVLDGYNAPVTAGNFIDLVERKFYDGMEIQRADGFVVQTGDPEGPAEGFIDPSTGKIRTVPLEIMVDGDKAP

VYGETLEELGRYKAQTKLPFNAFGTMAMAREEFDDNSASSQVFWLLKESELTPSNANILDGRYAVFGYVTENEDYLADVK

VGDVIESIQVISGLDNLVNPSYKIAG

>MUSAC06390 | GSMUA_Achr1G13990_00 | 1:1 ortholog | [Musa acuminata]

MQKDKSFGLKESAISLALAIGLFTGVPALTSPAHASPARPVLPDLSVLISGPPIKDPGALLRNALPIDNKAIREVQRPLE

DITESLKVSGVRALDLVERNIRQASRALEQGKTLILNGVAESKKEHGKELIEKLAVGMQEFQQIVEQRDRDAVAPKQKEL

LQYVGGVEEDMVDGFPYEVPEEYSNMPLLKGRATVDMKVKVKDNPNVQDCVFRIVLDGYNAPVTAGNFLDLVERHFYDGM

AIQRADGFVVQTGDPEGPAEGFIDPSTEKVRTIPLEIMVIGDKTPIYGETLEELGRYKAQTCLPFNAFGTMAMARDEFDN

NSGSSQVFWLLKESELTPSNANILDGRYAVFGYVTENEDYLADLKVGDVIESIQVVSGLENLVNPSYKIVG

>NICAT04955 | A0A1J6IL97 | 1:1 ortholog | [Nicotiana attenuata]

MAASVSCHYFSSPLTTSTKPIKHSLNYTHFAPSLNKVPIRHFAPLCSSQRSRPSEIQEKEKDWFLSLKKCAVSVALSVSL

ISGLPGLEWLSPAHASTPALPDVSVLISGPPIKDPGALLRYALPIDNKAIREVQKPLEDITDSLKVAGVKALDSVERNTR

QASRALKQGKTLIVSGLAKSKADHGVELLNKLEVGLDELQKIVEDKNRDAVVSKQKELLNYVGGVEEDMVDGFPYEVPEE

YQNMPLLKGRATVDMKVKVKDNPNVEECVFRIVLDGYNAPVTAGNFIDLVERHFYDGMEIQRADGFVVQTGDPEGPAEGF

IDPSTEKIRTVPLEIMVDGEKVPFYGETLEDLGLYKAQTRLPFNAFGTMAMAREEFENNSASSQVFWLLKESELTPSNAN

ILDGRYAVFGYVTENEDFLADLKVGDVIDSIQVVSGLDNLVNPSYKIAG

>SOLLC14209 | K4BBJ8 | 1:1 ortholog | [Solanum lycopersicum]

MAASVSCHYFSSPLTTSTKSIKPKLNYSHFAPALSKGSIRHFVPLCSSQRSLPSESECQDKEKDWFLSLKKCAAAVALSV

SLISGLPGSQWLGPAHASTPALPDVSVLISGPPIKDPGALLRYALPIDNKAIREVQKPLEDITDSLKIAGVKALDSVERN

TRQASRALKEGKTLIISGVAKSKTDHAIELLNKLEAGLGELQQIVEDRTRDAVAPKQKELLNYVGGVEEDMVDGFPYEVP

EEYQNMPLLKGRATVDMKVKVKDNPNLEECVFRIVLDGYNAPVTAGNFIDLVERHFYDGMDIQRADGFVVQTGDPEGPAE

GFIDPSTEKTRTVPLEIMVVGEKAPFYGETLEDLGLYKAQTRLPFNAFGTMAMAREEFENNSASSQVFWLLKESELTPSN

ANILDGRYAVFGYVTENEDFLADLKVGDVIESIQVVSGLDNLVNPSYKIAG

>CUCSA10516 | A0A0A0LHY2 | 1:1 ortholog | [Cucumis sativus]

MAAIVSCKFCSSLTTSKSSIPTSHYRSSKLWNGACSNPVGPRCSSFRRIKCGLQNVKKGRPFCLKEFAISSALAFSLITG

VPGLGPSADAYAVADPVIPELSVLISGPPIKDPGALLRYALPIDNKAIREVQKPLEDISESLKIAGVKALDSVERNVRQA

SRTLKQGKNLIISGLAESKKEHGVEVLGKLEVGLDELQQIVEDRNRDAVAPKQKELLNYVGSVEEDMVDGFPYEVPEEYK

NMPLLKGRAAVDMKVKVKDNPNIDECVFHIVLDGYNAPVSAGNFVDLVERHFYDGMEIQRADGFVVQTGDPEGPAEGFID

PSTEKTRTVPLEIMVEGEKAPFYGETLEELGLYKAQTKLPFNAFGTMAMAREEFDNNSASSQVFWLLKESELTPSNSNIL

DGRYTVFGYITENEDFLADLKVGDVIESMQVVSGLDNLVNPSYKIAG

>LOTJA20828 | Lj2g3v1014320 | 1:1 ortholog | [Lotus japonicus]

MDEDVLLILLQVSLLLHNTIFSKPYLCNLSSTSISTVSFSVSHELSSLISMAAITPCHYFNTHSTRLTYPLPHTNARLRG

FTARCSYQPPQHSQFQNKHEGRSFSLKHCAVSIALAVGLVTGVPTLGWPTDANAASPVLSDLSVLISGPPIKDPGALLRY

ALPIDNKAVREVQKPLEDITDSLKIAGVKALDSVERNTRQASRALTQGKTLIISGLAESKKEHGVELLNKLEAGMDELEL

IIQDRNRDAVGPKQKELLQYVGGVEEDMVDGFPYEVPEEYQNMPLLKGRAEVDMKVKVKDNPNLDECVFHIVLDGYNAPL

TAGNFVDLVERHFYDGMEIQRADGFVVQTGDPEGPAEGFIDPSTEKTRTIPLEIMADGEKAPFYGATLEELGLYKAQTKL

PFNAFGTMAMAREEFEDNSGSSQVFWLLKESELTPSNANILDGRYAVFGYVTKNEDYLADLKVGDVIESIQVVSGLDNLV

NPSYKIAG

>MANES16064 | A0A2C9UIA1 | 1:1 ortholog | [Manihot esculenta]

MAAIIPCHFCSSLSNSTLTISPKLPRNHFGFANAFRINAAWWARRPAPRCSLRNSHQPQFSDKQKGKPFSLKECAISLAL

AVGLITGVPSLDWSTIAYAASPALPDLSVLISGPPIKDPGALLRYALPIDNKAIREVQKPLEDITDSLKVSGVKALDSVE

RNVRQASRALKQGKSLIISGLAESKKDHGVELLDKLEAGMDELQQIVEDRNRDGVAPKQKELLNYVGGVEEDMVNGFPYE

VPEEYQSMPLLKGRAAVDMKVKVKDNPNVDECVFHIVLDGYNAPVTAGNFVDLVERHFYDGMEIQRADGFVVQTGDPEGP

AEGFIDPSTEKTRTIPLEIMVNGEKAPFYGATLEELGLYKAQTKLPFNAFGTMAMARDEFENNSASSQVFWLLKESELTP

SNANILDGRYAVFGYITENEDYLADLKVGDVIESMQVVSGLDNLVNPSYKIAG

>POPTR39977 | U5GVV7 | 1:1 ortholog | [Populus trichocarpa]

MAAIIPCHYCSSMAISKLPRSHIPLGNINHVNAAWWGRQLSLRCSLKASQKAQLRNKLKVKLFSLKECAISIALAVGLLT

GMPFVDWSPNAYAANPAMPDLSVLISGPPIKDPGALLRYALPIDNKAIREVQKPLEDITDSLKVAGVKALDSVERNLRQS

SQALKQGKSLIISGLAESKKDHGVELLDKLETGMDELQQIVVDRNRDAVAPKQKELLSYVGSVEEDMVDGFPYEVPEEYQ

SMPLLKGRATVDMKVKVKDNPNADEFMFRIVLDGYSAPVTAGNFLDLVQRHFYDGMEIQRADGFVVQTGDPEGPAEGFID

PSTEKTRTIPLEIMVNGEKSPFYGATLEELGLYKAQTRLPFNAFGTMAMARDEFENNSASSQVFWLLKESELTPSNANIL

DGRYAVFGYVTENEDYLADLKVGDVIESIQVVSGLDNLVNPSYKIAR

>PRUPE08377 | M5WTM9 | 1:1 ortholog | [Prunus persica]

MAAILSCHHSASPATSSKFISPSIPTNRTRFSISHKQFGTRSSSPLPIKCQIQNRQQKGKSFSIKECAISIALAVGLVTG

VPALEWSSNAYAANPVLPDLSVLISGPPIKDPGALLRYALPINNKAIREVQKPLEDITESLKVAGVKALDSVERNLRQAS

RALKQGKALIVSGLAESKKDHGVELLGKLEAGMDELQQIVEDRNRDGVAEKQKELLQYVGGVEEDMVDGFPYEVPEEYQS

MPLLKGRATVDMKVKVKDNPSLTDCVFRIVLDGYNAPVTAGNFVDLVERHFYDGMEIQRADGFVVQTGDPEGPAEGFIDP

STEKTRTIPLEITVEGEKLPFYGSTLEELGLYKAQTKLPFNAFGTMAMARDEFENNSASSQIFWLLKESELTPSNSNILD

GRYAVFGYVTQNEDFLADLKVGDVIESIQVVSGLENLANPSYKIAG

>ARATH17271 | CYP38_ARATH | 1:1 ortholog | [Arabidopsis thaliana]

MAAAFASLPTFSVVNSSRFPRRRIGFSCSKKPLEVRCSSGNTRYTKQRGAFTSLKECAISLALSVGLMVSVPSIALPPNA

HAVANPVIPDVSVLISGPPIKDPEALLRYALPIDNKAIREVQKPLEDITDSLKIAGVKALDSVERNVRQASRTLQQGKSI

IVAGFAESKKDHGNEMIEKLEAGMQDMLKIVEDRKRDAVAPKQKEILKYVGGIEEDMVDGFPYEVPEEYRNMPLLKGRAS

VDMKVKIKDNPNIEDCVFRIVLDGYNAPVTAGNFVDLVERHFYDGMEIQRSDGFVVQTGDPEGPAEGFIDPSTEKTRTVP

LEIMVTGEKTPFYGSTLEELGLYKAQVVIPFNAFGTMAMAREEFENDSGSSQVFWLLKESELTPSNSNILDGRYAVFGYV

TDNEDFLADLKVGDVIESIQVVSGLENLANPSYKIAG

>THECC13019 | A0A061ED69 | 1:1 ortholog | [Theobroma cacao]

MAAIIFCNCYSSLAASKWVNPRIPTKGTSLLRTQKSSSWLNRQLLPKCTSQKHVQCHLQDGQKGRSFSLKECAVSIVLAA

GLITGMPSLDWSPNAYAASPALPDLSVLISGPPIKDPGVLLRNALPINNKAVREVQKPLEDITESLKIAGVKALDSVERN

VRQASRALKQGKTLIISGLAETKKDHGVELLDKLEVGMDELQQIVEDRDRDAVAPKQKELLQYVGDVEEDMVDGFPYEVP

EEYRSLPLLKGRAAVDMKVKVKDNPNLDECVFHIVLDGYNAPVTAGNFVDLVQRHFYDGMEIQRADGFVVQTGDPEGPAQ

GFIDPSTEKTRTIPLEIMVNGEKAPFYGATLEELGLYKAQTKLPFNAFGTMAMARDEFENDSASSQVFWLLKESELTPSN

ANILDGRYAVFGYVTENQDFLADLKVGDVIESIQVVSGLDKLVNPSYKIAG

>COCAP22832 | A0A1R3IDS8 | 1:1 ortholog | [Corchorus capsularis]

MAAFISCNSYSSLAVSKWTNPRLPTKGTSFLRTQRSSSWFTRPFLSKCTPQKLVQCQLQNGQKGKSFSLKECAISILLAA

GLITGMPSLDWSPNAYATNPALSDLSVLISGPPIKDPGALLRYALPINNKAIREVQKPLEDITESLKVAGVKALDSVERN

ARQASRSLKQGKSLIISGLAESKKDHGVELLNKLEVGMEELQQIVEDRNRDAVAPKQKELLQYVGDVEEDMVDGFPYEVP

EEYRSMPLLKGRAAVDMKVKVKDNPNLEECVFHIVLDGYNAPVTAGNFVDLVQRHFYDGMEIQRADGFVVQTGDPEGPAE

GFIDPSTEKTRTIPLEIMVDGEKEPFYGATLEELGLYKAQTKLPFNAFGTMAMAREEFENNSASSQVFWLLKESELTPSN

ANILDGRYAVFGYVTENEDFLADLKVGDVIESIQVVSGLDNLVNPSYKIAG

>VITVI25978 | D7TWR2 | 1:1 ortholog | [Vitis vinifera]

MAAFISCHACSFLSTSKPVTPRIPTNHFRFPVSQRSAWTRKLGPRCFSQYPPPFQNQKKAKSFSLKECAISIALAVGLVT

GVPAMDWCADAYAATPALPDLSVLISGPPIKDPGALLRNALPIDNKAIREVQKPLEDISESLKLAGVKALDSVERNVRQA

SRALKQGKSLIIAGLAESKKENGVELLGKLEVGMDELEQIVVDRNRDAIAPKQKELLQYVGDVEEDMVDGFPYEVPEEYQ

NMPLLKGRATVDMKVKVKDNPNLDECVLRIVLDGYNAPVTSGNFLDLVERHFYDGMEIQRADGFVVQTGDPEGPAEGFID

PSTEKPRTVPLEIMVEGEKIPFYGATLEELGLYKAQTKLPFNAFGTMAMARDEFDDNSASSQIFWLLKESELTPSNANIL

DGRYAVFGYVTENEILLADLKVGDVIESIQVVSGLDNLVNPSYKIVAG

>AMBTC12159 | W1PHN6 | 1:1 ortholog | [Amborella trichopoda]

MAAAILCCYCTPSSASPKQGTFDNSKARFCYGGKPGRFRGITMSQCKSSNNYSRFSPIEHKRVEMKDEGFVCLRKCAFAV

ALSLGLFSGGSFGGIASAFEPKPVLPDLAVLISGPPIKDPGALLRYALPINNKAIIEVQKPLEDITESLKIAGTKALDSV

ERNVRQASRTLKQGKDMIISGIAPSKKEHGEELLGKLEVGLEELQNIVEERDRDRVALKQKELLNYVGGVEEDMVDGFPY

EVPEEYSGMPLLKGRATVDMKVKVKDNPNLEDCVFRLVLDGYNAPVTAGNFLDLVERHFYDGMEIQRADGFVVQTGDPEG

PAEGFIDPSTEKIRTIPLELMVSGDKAPVYGATLEELGRYKAQTKLPFNAFGTMAMARDEFEDNSASSQIFWLLKESELT

PSNANILDGRYTVFGYVTENQDFLADLKVGDVIESMEVVSGLDNLVNPSYKIAG

>PHYPA12958 | A9T157 | 1:n ortholog | [Physcomitrella patens subsp. patens]

MAVAAVLQAASLATTTTWLDAGAFRKEGLIGAQCVHKIVSARGPCAIRASLSKDDVAAPNAFAKVLHNCPTCVAVAAALL

VPFSSTAADVDVDTERVSAISEPSVLISGPPTKDANALLRDALPLDNKPIKEVQKFLEDITEEMKVPDEKVLEPVERDVR

QATLVLNQSKEEILSYIAESKRQEGDHILTTLNEELEGFQKQLEEKDSSIVLPRQKELLRLIGDLEETMIAKFPYEIPEE

YAKRPLLMGRATLEMKVLVKNNPNVKDAVLHIVLDGYNAPVTAGNFVDLVERRFYDGMEIQRSDGFVLQTGDPDGPAEGF

VDPATGQVRTVPLEIMVEGQKQPIYGTTLEELGLPKAKIKLPFNAFGTMAMARQESDNNSGSSQVFWLLKESELTPNGDS

IHDGRYAVFGYVTENEGLLANLKAGDVIESIRVLHGLDNLVNPSYVVRLL

>CHEQI28693 | XP_021727682 | 1:n ortholog | [Chenopodium quinoa]

MSSFICHHFYPSLCTSKHALPNNPIAPIYLGIPNFRDKARFSRLSTRCYSEVPRHIDSHDKQKKSSFSMKQCAISISLAI

GLITGAPDLNWQSHAQAITTPALPDLAVLISGPPIKDPEALLRYALPIDNKAIREVQKPLEDITDSLKVAGLKALDSVER

NLKQASRALKNGKSLIISGLAESKKDRGMELLDKLEIGMDELQQIVENRNRDGVAPKQKELLQYVGSVEEEMVDGFPYEV

PEEYRSMPLLKGRAVVQMKVKVKDNPNLEDCVFRIVLDGYNAPVTAGNFVDLVERHFYDGMEIQRADGFVVQTGDPEGPA

EGFIDPSTEQPRTVPLEIMVEGEKVPFYGATLEELGLYKAQTKLPFNAFGTMAMAREEFENNSASSQIFWLLKESELTPS

NANILDGRYAVFGYVTENEDYLADLKVGDVIESVQVVSGGENLVNPTYKIAG

>SOYBN34874 | I1N5T2 | 1:n ortholog | [Glycine max]

MAAIIPCHYCASLSPKWFNSNNTHSRRLSYPLRGLRGFNARCSYQPPHHSESQNNHNGRSFSLKQCAISIALAVGLITGV

PTLDGPTIAQAANPVLSDLSVLISGPPIKDPGALLRYALPIDNKAIREVQKPLEDITESLKIAGVKALDSVERNVRQASR

TLKQGKTLIVSGLAESKKEHGVELLSKLEAGIDELELIIQDRNRDAVAPKQKELLQYVGGVEEDIVDGFPFEVPEEYQNM

PLLKGRAAVDMKVKVKDNPNLDECVFHIVLDGYNAPVTAGNFVDLVERHFYDGMEIQRADGFVVQTGDPEGPAEGFIDPS

TEKIRTIPLEITVNGEKAPVYGSTLEELGLYKAQTKLPFNAFGTMAMARDEFEDNSASSQIFWLLKESELTPSNANILDG

RYAVFGYVTENEDNLADLKVGDVIESIKVVSGLDNLVNPTYKIAG

>BRANA69847 | A0A078IGH2 | 1:n ortholog | [Brassica napus]

MAASFATLPTVSLVNTSRRRIDSYSSKKRVGVVRCCSGGDIPEYKQVQRGGGGGGTLKECAITLALSLGLIAGGGAPSIA

YAANPAIPQVSVLISGPPIKDPGALLRYALPIDNKAIREVQKPLEDITDSLKIAGVKALDSVERNVRQASRSLQQGKSMI

VAGFAESKKDHGHELIGKLEAGMQDMLQIVEDRKRDAVAPKQKEILQYVGGIEEDMVDGFPYDVPEEYRNMPLLKGRATV

DMKVKIKDNPNLEDCVFRIVLDGYNAPVTAGNFVDLVERHFYDGMEIQRSDGFVVQTGDPEGPAEGFIDPSTEKVRTVPL

EIMVEGKKTPFYGSTLEELGLYKAQVMLPFNAFGTMAMAREEFENDSGSSQVFWLLKESELTPSNSNILDGRYAVFGYVT

QNEDFLADLKVGDVIESIQVVSGLDNLVNPSYKIAG

>GOSHI16559 | A0A1U8HQH8 | 1:n ortholog | [Gossypium hirsutum]

MAAVISCNSYSPLAASKWINPTLPTKASTFLATPKYNPHKHVQCKLQNEQKGRPFSLKECAISIILAAELITGIPSLDAY

AANPPLPDLSVLISGPPIKDPGALLRYALPINNKAVREVQKPLEDITESLKIAGVKALDSVERNVRQASRALKQGKMLII

SGLAEAKKDRGAELLDKLEVGMEELQQIVEDRNRDAVAPKQKELLQYVGDVEEDMVDGFPYEVPEEYRNMPLLKGRAAVD

MKVKVKDNPNLEECVFRIVLDGYNAPVTAGNFVDLVQRHFYDGMEIQRADGFVVQTGDPDGPAEGFIDPSTEKTRTIPLE

IMVDGEKAPVYEATLEELGLYKAQTKLPFNAFGTMAMAREEFENNSASSQIFWLLKESELTPSNANILDGRYAVFGYVTE

NEDFLADVKVGDVIESIKLVSGQDNLVNPSYKIAG

>SELML26057 | D8R8R1 | 1:n ortholog | [Selaginella moellendorffii]

MQVAAPFSDWNLPVANAILYSPDTKVPRTAEVALRRAIPVITPTMKAIQEPLENIFYLLRIPQRKPYGSMESDVKKALKI

VMDGKDTIISSIPEEKRATGQELYDTLVDEKAGLPALLGSIGKQDADKVSIRLASSLDVIAQIELLQAPALPYLLPAQYQ

QLPRLTGRALVEMVVKKGDNTAFTVAAGQGPQPQGTIQVVLDGFSAPITAGNFADKVLKGFYNGVKLRTTEQAILSDSAQ

QDALPMEILPSGEFQPLYKTTLNIQDGELPVLPLSVYGAVAMAHDPSAEDLSSASQFFFYLYDRRSAGLGGLSFEEGQFA

VFGYATKGRELLSQLKTGDVIESATLVSGRDRLVYPQAPS

>NP_296262.1 cyclophilin-type peptidyl-prolyl cis-trans isomerase [Deinococcus radiodurans R1]

MSLQDGQDYYALLDTNRGQVLIDLYEQETPVTVNNFVTLARNHFYDGLRFHRVIDGFMAQTGDPKSADDAQKDAWGSGGPGYQFPDEVRSKLTFDTGGQLAMANSGPNTNGSQFFVTFGPAAFLDGHYSLFGKVVTGDDVLAKLTRTAETSGGQETPIAGAVPDKLLSVRILTKNK

>NP_358273.1 peptidyl-prolyl cis-trans isomerase [Streptococcus pneumoniae R6]

MKKLATLLLLSTVALAGCSSVQRSLRGDDYVDSSLAAEESSKVAAQSAKELNDALTNENANFPQLSKEVAEDEAEVILHTSQGDIRIKLFPKLAPLAVENFLTHAKEGYYNGITFHRVIDGFMVQTGDPKGDGTGGQSIWHDKDKTKDKGTGFKNEITPYLYNIRGALAMANTGQPNTNGSQFFINQNSTDTSSKLPTSKYPQKIIEAYKEGGNPSLDGKHPVFGQVIGGMDVVDKIAKAEKDEKDKPTTAITIDSIEVVKDYDFKS

>NP_057143.1 peptidyl-prolyl cis-trans isomerase-like 1 [Homo sapiens]

MAAIPPDSWQPPNVYLETSMGIIVLELYWKHAPKTCKNFAELARRGYYNGTKFHRIIKDFMIQGGDPTGTGRGGASIYGKQFEDELHPDLKFTGAGILAMANAGPDTNGSQFFVTLAPTQWLDGKHTIFGRVCQGIGMVNRVGMVETNSQDRPVDDVKIIKAYPSG

>NP_081121.1 peptidyl-prolyl cis-trans isomerase-like 1 [Mus musculus]

MAAIPPDTWQPPNVYLETSMGVIVLELYWKHAPKTCKNFAELARRGYYNGTKFHRIIKDFMIQGGDPTGTGRGGASIYGKQFEDELHPDLKFTGAGILAMANAGPDTNGSQFFVTLAPTQWLDGKHTIFGRVCQGIGMVNRVGMVETNSQDRPVDDVKILKAYPSG

>NP_417822.1 peptidyl-prolyl cis-trans isomerase A (rotamase A) [Escherichia coli str. K-12 substr. MG1655]

MFKSTLAAMAAVFALSALSPAAMAAKGDPHVLLTTSAGNIELELDKQKAPVSVQNFVDYVNSGFYNNTTFHRVIPGFMIQGGGFTEQMQQKKPNPPIKNEADNGLRNTRGTIAMARTADKDSATSQFFINVADNAFLDHGQRDFGYAVFGKVVKGMDVADKISQVPTHDVGPYQNVPSKPVVILSAKVLP

>NP_493378.1 CYclophyliN [Caenorhabditis elegans]

MEPVFKEPSPKKKRVENPPENAPDAPKKPQLKHESEFLRSIPSSSQYEKSFMHRDTISHVIATKTDFIITASVDGHLKFWKKKHSEGVEFVKHFRCHLSEFSHICANIDGTLLATVCEADKSVKVFDIENFDMINMIKLDFPPKTANWVHQSNDPIAHLAIGAADSGKIIVVDGKATAAPICIKDKLHSTPVKIIEYSQSLDIIVSIDESGMIECWNGERGDFQFPETKLTWEYKLETDLYDFVKAKTIPVCATFDPSGLKLATFAEDRKIRIFNVKTGKLAQLIDETTQKYHCEAKENKNYGLQHMEWSRRLASEKEMDKDKKNSLKYTKICFDQSGNFLLYPTPIGVKVFNLVTNEVSRTIGRDEAIRFVAVSLCNALPDIRQKLQGAAITLETAAADNPTLNRKTDPDPLMVCCALRKNRFYLFTNTEPYNVEDDEGNPSVSGRDVFNERPKKEDLLTALDTEGGEKVLNKEAIIHTSFGDITIRLFGDECPKTVENFCTHSRRGYYNGLTFHRVIKSFMIQTGDPSGKGTGGESIWGEDFEDEFHPRLRHDKPFKVSMANAGGGNTNGSQFFITVCPADWLDGKNTLFGEVTAGMSVVQRINQVSTFERSGRPRESIQIMSISLK

>NP_594502.2 cyclophilin family peptidyl-prolyl cis-trans isomerase Cyp8 [Schizosaccharomyces pombe]

MGKNTDKLYITQTEHSGVHGWHGGMSGIAQKNSTTSYKQLPFNYCSLSLQPFNHPCCLVDETKQAIIFDFRFIVPWLRKHGTNPINGQKASMSDLIKLKFAKNSAEEYCDPVTMKSFTRFSHIVAIRSTGNCFSWDTIERLNIKPKHWRDLVNEEQFTRDDIITIQDPHNVENRDFSAIQKQKETARDEKITKAKIALQASRAKSTESTSSPELSHSLDSSKSIASDMPIHRASHTTGYAAASLTSTSFTPVTKNERAIIAEEDYMLNHTRIKHKGYARIVTNHGEINIELHTDYAPHAVYNFVQLAKQGYYRNTIFHRNIARFMIQGGDPSGTGRGGQSIWGKPFKDEFCNPLKHDDRGIISMANRGKNTNGSQFFILYGPAKHLDNKHTIFGRVVGGLNVLDALEKVPTNSNDHPKLPIKLEDIIIFVDPFEEWKKDEREKEKRKRQEEEEENNLDRTSWTGRDLSASSTDHSLNASVGKYLKKEVSLEEKTFTSTVNPKKKKARTGFGNFDAW

>NP_011924.1 peptidylprolyl isomerase CPR2 [Saccharomyces cerevisiae S288C]

MKFSGLWCWLLLFLSVNVIASDVGELIDQDDEVITQKVFFDIEHGEEKVGRIVIGLYGKVCPKTAKNFYKLSTTTNSKKGFIGSTFHRVIPNFMVQGGDFTDGTGVGGKSIYGDTFPDENFTLKHDRKGRLSMANRGKDTNGSQFFITTTEEASWLDGKHVVFGQVVDGMDVVNYIQHVSRDANDKPLEAVKIAKCGEWTPELSS

>NP_523874.1 Cyclophilin-like [Drosophila melanogaster]

MLSLSDPNNAGGIPDKAWQPHFVTLETSMGEITVELYWKHAPNTCRNFAELSRRGYYNNVVFHRIIRDFMIQGGDPTGTGRGGASIYGSEFADELHGDLRHTGAGILSMANSGPDTNGSQFFITLAPTQWLDGKHTIFGRVYTGMEVVKRIGMVETDKNDRPVDPLRIIKAKVEKL
